# Transcriptome and DNA Methylome Analysis in a Mouse Model of Diet-Induced Obesity Predicts Increased Risk of Colorectal Cancer

**DOI:** 10.1016/j.celrep.2017.12.071

**Published:** 2018-01-16

**Authors:** Ruifang Li, Sara A. Grimm, Deepak Mav, Haiwei Gu, Danijel Djukovic, Ruchir Shah, B. Alex Merrick, Daniel Raftery, Paul A. Wade

**Affiliations:** 1 Epigenetics and Stem Cell Biology Laboratory, National Institute of Environmental Health Sciences, Research Triangle Park, NC 27709, USA; 2 Integrative Bioinformatics, National Institute of Environmental Health Sciences, Research Triangle Park, NC 27709, USA; 3 Sciome, LLC, 2 Davis Drive, Research Triangle Park, NC 27709, USA; 4 Biomolecular Screening Branch, National Toxicology Program, National Institute of Environmental Health Sciences, Research Triangle Park, NC 27709, USA; 5 Northwest Metabolomics Research Center, Department of Anesthesiology and Pain Medicine, University of Washington, 850 Republican St., Seattle, WA 98109, USA; 6 Public Health Sciences Division, Fred Hutchinson Cancer Research Center, 1100 Fairview Ave. N., Seattle, WA 98109, USA

## Abstract

Colorectal cancer (CRC) tends to occur at older age; however, CRC incidence rates have been rising sharply among young age groups. The increasing prevalence of obesity is recognized as a major risk, yet the mechanistic underpinnings remain poorly understood. Using a diet-induced obesity mouse model, we identified obesity-associated molecular changes in the colonic epithelium of young and aged mice, and we further investigated whether the changes were reversed after weight loss. Transcriptome analysis indicated that obesity-related colonic cellular metabolic switch favoring long-chain fatty acid oxidation happened in young mice, while obesity-associated downregulation of negative feedback regulators of pro-proliferative signaling pathways occurred in older mice. Strikingly, colonic DNA methylome was pre-programmed by obesity at young age, priming for a tumor-prone gene signature after aging. Furthermore, obesity-related changes were substantially preserved after short-term weight loss, but they were largely reversed after long-term weight loss. We provided mechanistic insights into increased CRC risk in obesity.

## In Brief

Li et al. find that obesity-induced DNA methylation changes reprogram the colonic transcriptome, leading to a metabolic switch favoring long-chain fatty acid oxidation in young mice and a more tumor-prone gene signature after aging. Obesity-related changes are substantially preserved after short-term weight loss, but they are largely reversed after long-term weight loss.

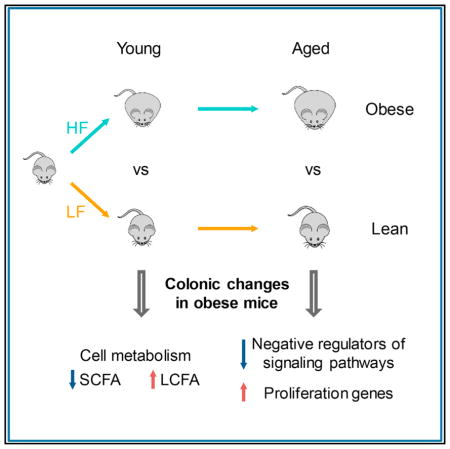

## INTRODUCTION

Colorectal cancer (CRC) is the third most common cancer worldwide (Ferlay et al., 2015). CRC tends to occur at a later age (>50 years old), however, CRC incidence rates have been increasing dramatically among young age groups (Siegel et al., 2017). The increasing prevalence of obesity is recognized as a major risk for CRC (Renehan et al., 2008), yet the molecular underpinnings of the link remain incompletely resolved, and even less is known about the molecular events that initiate the process. Mechanistic insights are urgently needed to pave the way for effective prevention of CRC development in obese humans. Weight loss seems to be a logical strategy to reduce CRC risk, but it is unclear whether obesity-associated changes in the colonic epithelium can be reversed after weight loss.

Epigenetic mechanisms are fundamental to phenotypic changes induced by environmental and lifestyle factors. Regarding epigenetic mechanisms underlying the link between obesity and CRC, DNA methylation is a prime candidate. First, aberrant DNA methylation is observed in virtually all CRCs (Lao and Grady, 2011); second, DNA methylation responds to obesity and weight loss leading to gene expression changes (Barres et al., 2013); and third, DNA methylation changes can persist even after the original stress/stimulus is gone (Chen et al., 2016).

To mimic human obesity, we employed a diet-induced obesity mouse model, which exhibits metabolic dysfunctions (Collins et al., 2004) and increased incidence of colon cancer (Tuominen et al., 2013) as in obese humans. To understand the mechanisms of how obesity and weight loss shape the predisposition to CRC in mice at physiologically relevant settings as in humans (Jackson et al., 2017), we examined molecular pathophysiologic changes in the colonic epithelium at two time points that are equivalent to human young (~30 years old) and older age (~50 years old). Six-week-old male C57BL/6J mice were fed a low-fat diet for 20 (or 43) weeks (LF: control mice), a high-fat diet for 20 (or 43) weeks (HF: obese mice), or a high-fat diet for 15 weeks and then switched to a low-fat diet for another 5 (or 28) weeks (HF-LF: formerly obese mice). To globally map obesity-associated changes in DNA methylation and gene expression, we performed whole-genome bisulfite sequencing (WGBS) and RNA sequencing (RNA-seq) analyses in the colonic epithelium from those mice (Table S1). We first compared young and aged obese mice with their age-matched control mice to gain insights into obesity-induced molecular pathophysiological changes in the colon at different stages of life, and then we explored data from formerly obese mice to investigate whether those changes can be reversed after weight loss.

## RESULTS

### Dysregulation of Metabolic and Cancer-Related Genes in Colonic Epithelium of Young Obese Mice

**Figure 1:**
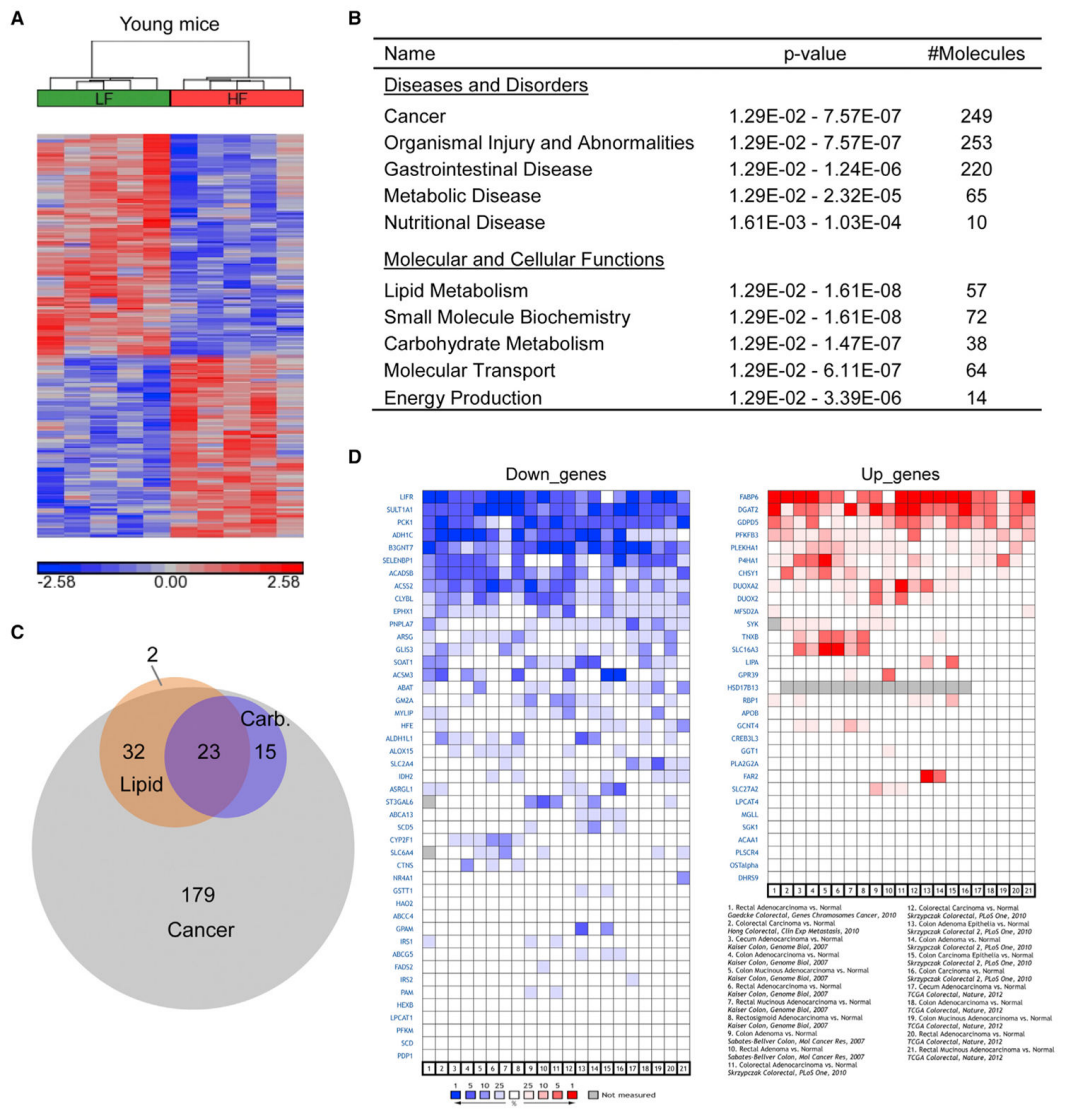
Obesity-Related DEGs in Young Mice Are Significantly Enriched with Metabolic and Cancer-Related Genes (A) The heatmap depicts the standardized expression levels of DEGs in young obese mice (HF) relative to control mice (LF). Red indicates overexpression and blue indicates underexpression. (B) IPA of obesity-related DEGs in young mice. The top five scoring hits in each functional category are shown, together with p values and the numbers of dysregulated genes in the enriched terms. (C) Overlap of dysregulated lipid and carbohydrate metabolism genes with dysregulated cancer-related genes in young obese mice. (D) The expression changes (blue, underexpression; red, overexpression) of lipid, carbohydrate, and amino acid metabolism genes that were dysregulated in young obese mice, in human CRC compared with normal colon/rectum. Left and right panels exhibit obesity-related down- and upregulated genes, respectively. See also Figure S1 and Tables S2 and S3.

Comparing young obese mice with age-matched control mice, 287 differentially expressed genes (DEGs) were identified using DESeq2 (Figure 1A; Table S2A). Gene expression changes were validated using real-time RT-PCR (Figure S1A). To understand the biological functions of obesity-associated DEGs, we performed Ingenuity Pathway Analysis (IPA) and gene ontology (GO) analysis. Obesity-associated DEGs were significantly enriched with genes involved in metabolic processes, such as lipid metabolism, carbohydrate metabolism, and energy production, and they were predominantly associated with cancer (249 of 287 DEGs) and gastrointestinal disease (220 of 287 DEGs) (Figure 1B; Table S2C). Since metabolic reprogramming is a hallmark of cancer (Hanahan and Weinberg, 2011), we asked whether dysregulated metabolic genes in young obese mice were related to cancer. Indeed, they largely overlapped with cancer-related DEGs (Figures 1C and S1B). We further examined whether those dysregulated metabolic genes were also differentially expressed in human CRC. The majority showed the same direction of change as in young obese mice in at least one dataset comparing human CRC with normal colon/rectum (Figure 1D). Taken together, we propose that obesity may predispose individuals to CRC via reprogramming cellular metabolism in the colonic epithelium.

### A Metabolic Switch Favoring Long-Chain Fatty Acid Oxidation in Colonic Epithelium of Young Obese Mice

Colonocytes normally use butyrate, a short-chain fatty acid derived from colonic fermentation of dietary fiber, as a major energy source (Roediger, 1982). Since fecal butyrate production was reduced in obese mice (data not shown), colonocytes in obese mice may switch to other energy sources. In concordance with the decrease in butyrate, we observed obesity-related downregulation of Acyl-CoA synthetases (*Acss1*, *Acss2*, and *Acsm3*) and Acyl-CoA dehydrogenase (*Acadsb*) that are specifically responsible for short- and medium-chain fatty acid activation and dehydrogenation during fatty acid oxidation (Table S2A). In contrast, we noticed obesity-related upregulation of *Slc27a2* and *Acaa1b* (Table S2A), which are engaged in long-chain fatty acid activation and thiolysis, respectively, implying an increase in long-chain fatty acid oxidation; consequently, the level of C12 acylcarnitine, an intermediate metabolite of long-chain fatty acid oxidation, increased in the colonic epithelium of young obese mice (Figure S1C). Consistent with the notion that long-chain fatty acid oxidation inhibits glucose utilization (Hue and Taegtmeyer, 2009), we saw concurrent downregulation of glucose metabolism genes, such as *Slc2a4* (glucose transporter), *Pfkm* (phosphofructokinase), and *Pdp1* (PDH phosphatase) (Table S2A). Collectively, our data indicate that obesity triggered a metabolic switch favoring long-chain fatty acid oxidation in the colonic epithelium. This likely reflects an adaptation to nutrient availability; nevertheless, it can boost colonic stem cell functions and promote colon tumorigenesis (Beyaz et al., 2016), as normal and cancer stem cells benefit from active fatty acid oxidation for their maintenance and function (Carracedo et al., 2013). Moreover, diminished utilization of butyrate and glucose in colonic epithelium of obese mice resembled the changes of cellular energy metabolism during colon tumorigenesis (Zhang et al., 1998).

### Altered Colonic Cellular Metabolic Profile in Young Obese Mice Resembles that of CRC

As a reflection of gene expression changes, the metabolome of colonic epithelium was also altered in young obese mice. In line with obesity-associated upregulation of *Slc7a9*, an amino acid transporter (Table S2A), targeted metabolomics analysis revealed significant increases in the levels of most amino acids in the colonic epithelium of young obese mice (Table S3A), reminiscent of a metabolic trait observed in human colon cancer (Hirayama et al., 2009). In addition, we detected a significant elevation in the level of acetylcholine (Figure S1D), which promotes colon cancer cell proliferation, migration, and invasion (Belo et al., 2011; Cheng et al., 2008). On the other hand, untargeted lipidomics analysis showed that phospholipids of specific molecular species were significantly reduced in the colonic epithelium from young obese mice (Table S3B), in keeping with obesity-associated dysregulation of phospholipid metabolism genes, such as *Lpcat1* and *Lpcat4* (Table S2A). Collectively, our data demonstrate that obesity-associated gene expression changes resulted in a cellular metabolic profile reminiscent of that of colon cancer in many aspects.

### Obesity-Related DNA Methylation Changes Occur at Distal Regulatory Regions

To explore the epigenetic basis of obesity-associated gene expression changes, we performed WGBS in the colonic epithelium (average coverage >25× and ~82.5% of CpGs with coverage ≥10× per group; Table S1A). Additionally, we carried out deep sequencing (>2,500×coverage) of bisulfite PCR amplicons (BSP-seq) containing 233 randomly selected CpG sites. Methylation levels of those CpG sites showed a strong correlation between WGBS and BSP-seq (Figure S2A), suggesting that the WGBS data coverage is sufficient. Since non-CpG methylation is rare in mouse colonic epithelium (data not shown), we studied only CpG methylation in detail. In all three experimental groups, the distribution of methylation level of single CpG sites was largely bimodal (Figure S2B), and promoters and CpG islands were lowly methylated while repetitive elements (LINE, SINE, and LTR) were highly methylated (Figure S2C), indicating that no global differences in DNA methylation were observed comparing the three groups.

**Figure 2:**
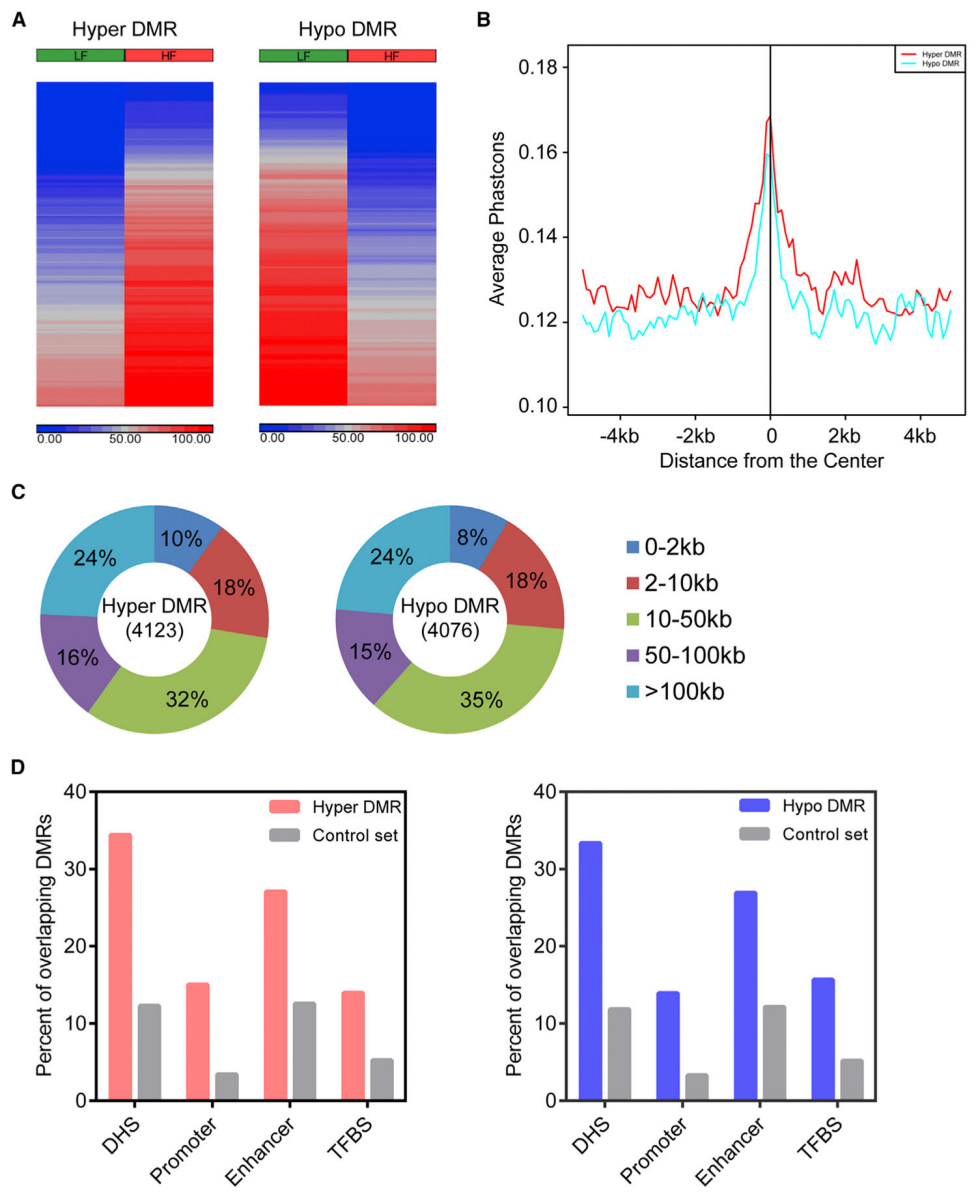
Obesity-Related DNA Methylation Changes Occur at Distal Regulatory Regions (A) The heatmaps depict DNA methylation levels of DMRs in young obese (HF) and control (LF) mice. Blue indicates unmethylated and red indicates fully methylated. (B) The underlying DNA sequences of DMRs are conserved across placental mammals. The average placental PhastCons score was plotted for a 10-kb window centered at the midpoint of DMRs. (C) Distance of DMRs to the nearest RefSeq gene transcription start site (TSS). (D) The percentages of DMRs or matched control regions overlapping with *cis*-regulatory regions defined using mouse ENCODE data. See also Figure S2 and Tables S1 and S4.

Next, we used the Rao Scott Likelihood Ratio Test to identify differentially methylated regions (DMRs); 4,123 regions gained DNA methylation (hyper-DMRs) and 4,076 regions lost DNA methylation (hypo-DMRs) in obese mice compared with control mice (Figure 2A; Table S4A). Although most DMRs are outside of known gene promoters (Figure 2C), the underlying DNA sequences of obesity-associated DMRs are conserved across placental mammals (Figure 2B). We next examined whether obesity-associated DMRs overlapped with annotated genomic features and experimentally defined functional genomic elements. Higher percentages of DMRs than control regions coincided with regions marked by H3K4me1, H3K4me3, and DNase-hypersensitive sites (DHSs), whereas DMRs rarely occurred at CpG islands (Figure S2D). Furthermore, obesity-associated DMRs significantly overlapped with *cis*-regulatory regions (DHSs, promoters, enhancers, and TFBS) defined using mouse Encyclopedia of DNA Elements (ENCODE) data from over 100 mouse cell types and tissues (Figure 2D) (Yue et al., 2014). Notably, a larger number of DMRs overlapped with enhancers and H3K4me1-marked regions than with promoters and H3K4me3-marked regions (Figures 2D and S2D). Taken together, obesity-associated DNA methylation changes in the colonic epithelium mainly occurred at distal regulatory regions.

### Obesity-Related DMRs Are Significantly Associated with DEGs

**Figure 3:**
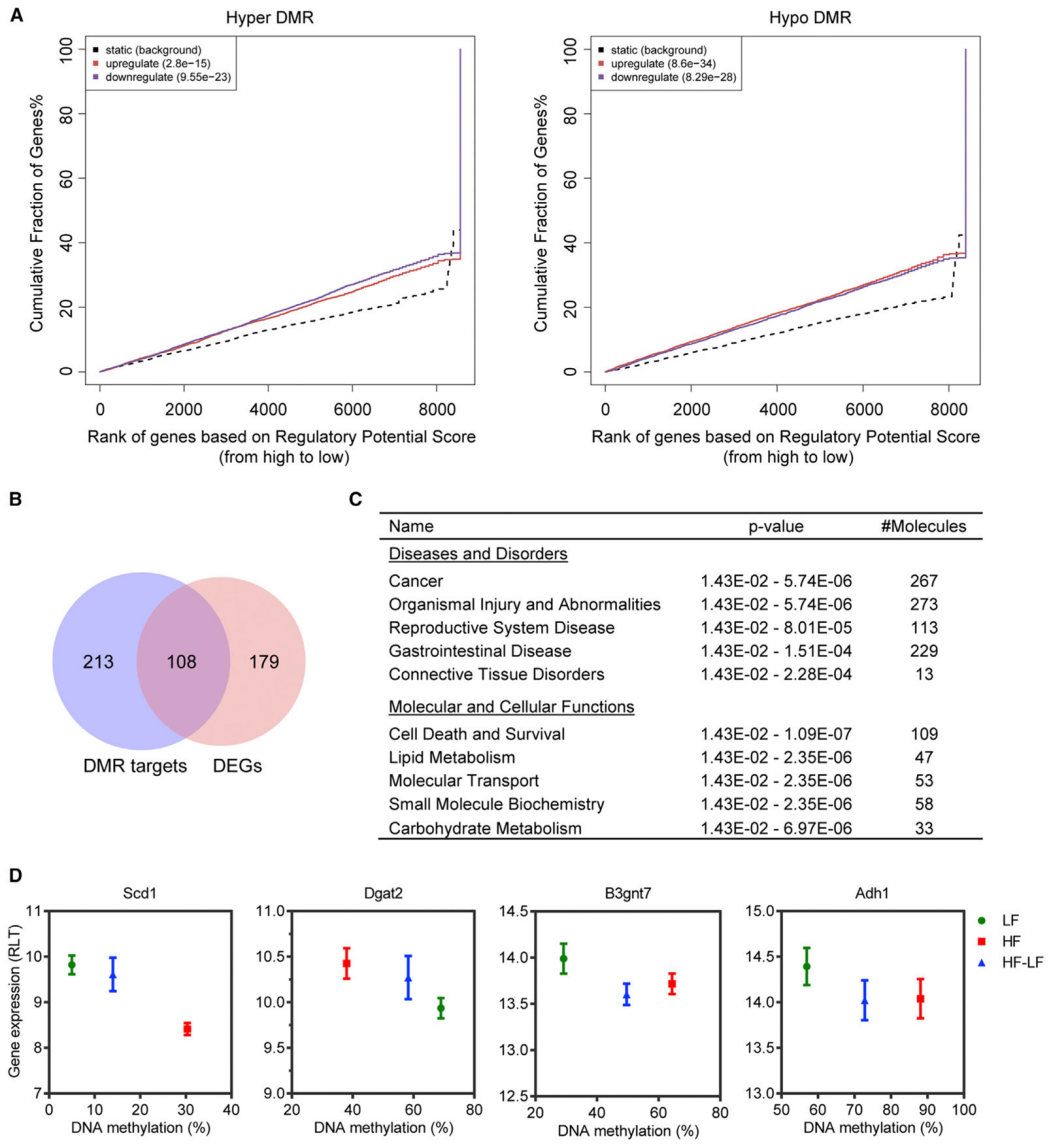
Obesity-Related DMRs Are Significantly Associated with DEGs (A) BETA with hyper-DMRs (left panel) or hypo-DMRs (right panel) and differential gene expression data (obese versus control) from young mice. The dotted line represents background genes not differentially expressed, whereas the red and the blue lines represent up- and downregulated genes in obese mice, respectively. The y axis indicates the proportion of genes in a category that are ranked at or better than the x axis value, which represents the rank on the basis of the regulatory potential score from high to low. The p values listed on the top left represent the significance of the UP or DOWN group relative to the NON group, as determined by Kolmogorov-Smirnov test. (B) Overlap of DMR target genes with obesity-related DEGs from young mice. (C) IPA of DMR target genes. The top five scoring hits in each functional category are shown, together with p values and the number of DMR target genes in the enriched terms. (D) Correlation between DNA methylation and gene expression at several metabolic genes. Error bars indicate SD. See also Figure S3.

Given that DMRs largely overlapped with regulatory regions in the genome, we hypothesized that obesity-related DNA methylation changes may affect gene expression. To test this hypothesis, we employed binding and expression target analysis (BETA) (Wang et al., 2013). BETA integrates differentially bound/modified regions (user-defined regions) with differential gene expression data to determine whether user-defined regions have overall activating and/or repressing functions and to detect their target genes. We ran BETA with hyper-DMRs and hypo-DMRs separately. Both types of DMRs were significantly enriched near both up- and downregulated genes, indicating that DNA methylation at distal regulatory regions may have both activating and repressing functions (Spruijt and Vermeulen, 2014); however, hyper-DMRs and hypo-DMRs were more significantly associated with downregulated and upregulated genes, respectively (Figure 3A). With a rank product (RP, equivalent to p value) cutoff of less than 0.001, BETA found 321 DMR target genes. A subset of them (108) were significantly differentially expressed (Figure 3B), while the rest exhibited a clear trend toward differential expression, although they did not reach statistical significance (Figures S3A and S3B), suggesting that obesity-associated DNA methylation changes are complementary to, but not exclusively overlapping with, changes in gene expression. Other than directly regulating transcription, altered DNA methylation may reflect historical changes or prime for future changes in gene expression.

To infer the biological impacts of obesity-associated DNA methylation changes, we performed IPA of DMR target genes. They were predominantly involved in cancer (267 of 321) and gastrointestinal disease (229 of 321), and they were significantly enriched with lipid and carbohydrate metabolism genes (Figure 3C). Correlations between DNA methylation and gene expression were shown at several metabolic genes (Figures 3D and S3C). Overall, obesity-associated DNA methylation changes were significantly associated with gene expression changes in the colonic epithelium, especially at metabolic genes related to cancer, in keeping with the notion that aberrant DNA methylation at distal regulatory regions regulates gene expression and modulates the predisposition to cancer (Aran and Hellman, 2013; Aran et al., 2013).

### Obesity-Related DNA Methylation Changes Also Prime for Future Changes in Gene Expression

**Figure 4:**
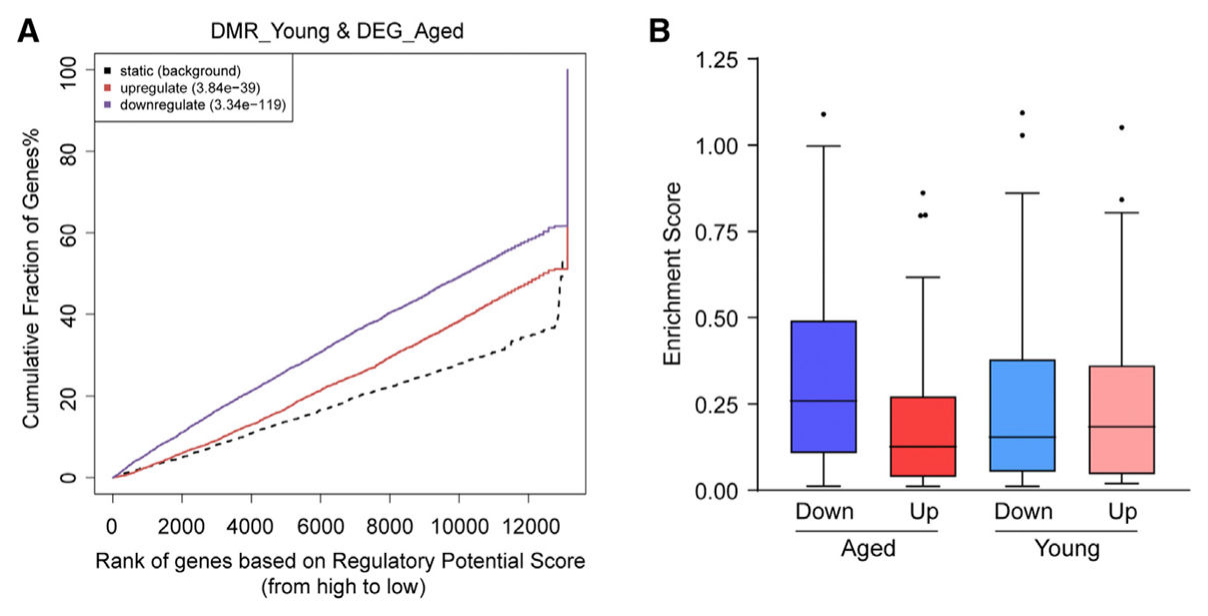
Obesity-Related DNA Methylation Changes at Young Age Prime for Future Gene Expression Changes after Aging (A) BETA with obesity-related DMRs from young mice and differential gene expression data from aged mice. (B) The enrichment scores of DMRs within 100 kb of obesity-related up- or downregulated genes from young and aged mice. In the box and whisker plot, the box indicates the 25th to 75th percentile, whiskers indicate 1.5 times the inter-quartile distance. See also Figure S4 and Table S4.

To test whether obesity-related DNA methylation changes prime for future gene expression changes at a later stage of life, we ran BETA with obesity-related DMRs from young mice and differential gene expression data from aged mice. We reasoned that, if that is true, those DMRs should also show significant association with obesity-related DEGs from aged mice. In fact, those DMRs exhibited even more significant associations with DEGs from aged mice, especially with downregulated genes, than with DEGs from young mice (Figure 4).

Next, we explored the mechanistic explanation for that. Since a substantial number of DMRs overlapped with regulatory regions (Figures 2D and S2D) and DNA methylation changes may affect transcription factor binding at those sites, we thus searched for transcription factor motifs enriched at obesity-associated DMRs using Hypergeometric Optimization of Motif Enrichment (HOMER) motif analysis (Heinz et al., 2010) (Table S4C). We then determined upstream regulators of obesity-related DEGs from aged mice using Ingenuity upstream regulator analysis (Table S5B). We found that obesity-associated DMRs from young mice are enriched with motifs of transcription factors that are upstream regulators of obesity-related DEGs from aged mice (Tables S4C and S5B). For example, DMRs are enriched with binding sites of ELK1, ELK4, and E2F transcription factors (Figure S4A). E2F family members play important roles in cell cycle progression, while ELK1 and ELK4 are cofactors of serum response factor (SRF) (Buchwalter et al., 2004), which regulates many immediate early genes (Schratt et al., 2001). Concordantly, we observed differential expression of their target genes, such as immediate early genes and genes related to cell cycle, in aged obese mice compared with age-matched control mice (Figures S4B and S4C). Altogether, our data suggest that obesity may imprint regulatory regions at young age, priming for future changes in gene expression at a later stage of life.

### A Tumor-Prone Gene Signature in the Colonic Epithelium of Aged Obese Mice

**Figure 5:**
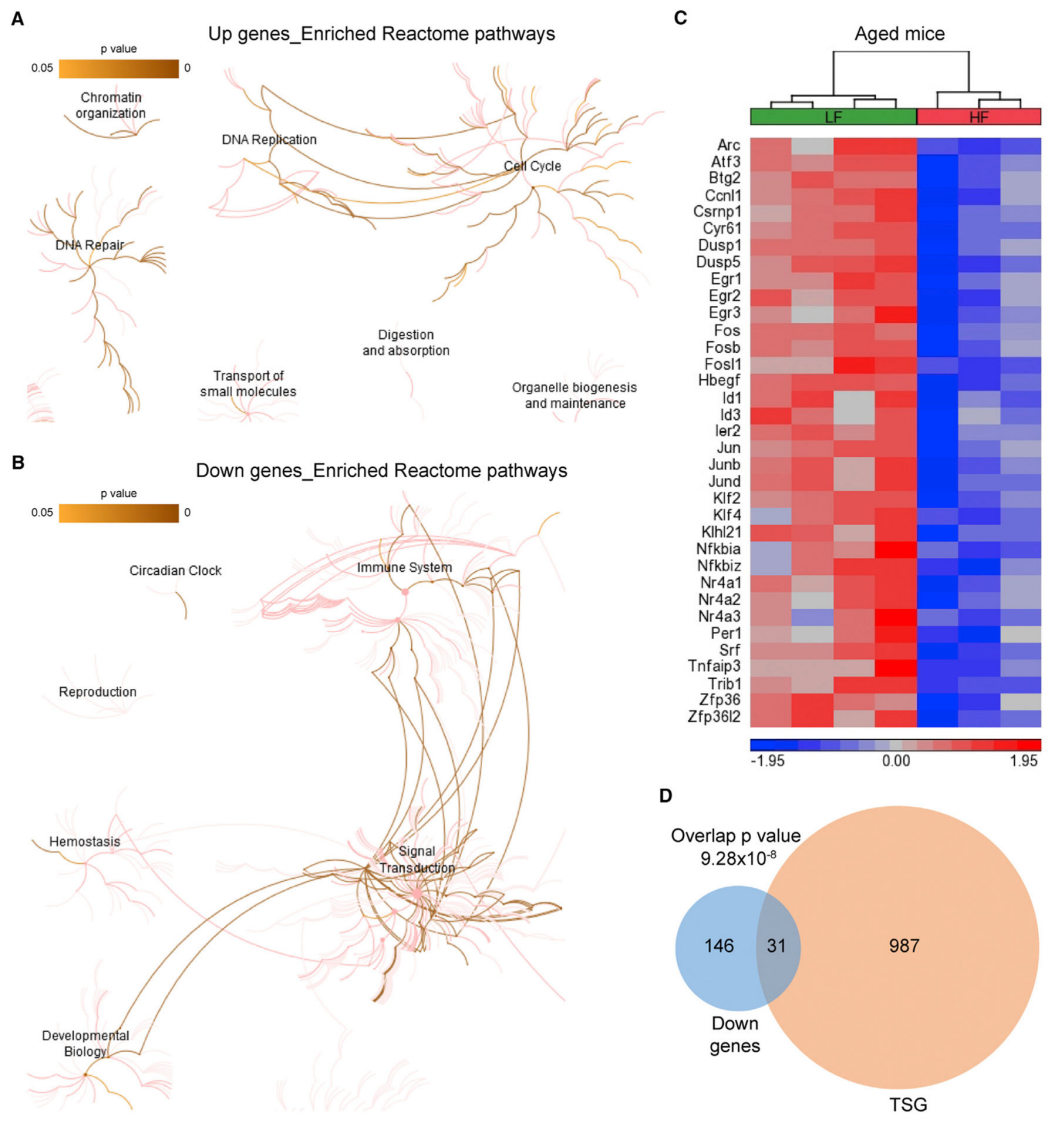
A Tumor-Prone Gene Signature in the Colonic Epithelium of Aged Obese Mice (A and B) The results of Reactome pathway enrichment analysis are shown for up- (A) and downregulated genes (B) in aged obese mice. The Reactome hierarchical pathway structure is shown with color corresponding to the significance of p values. The darker color is more significant. (C) The heatmap depicts the standardized expression levels of the 35 primary response genes in aged obese and control mice. (D) Overlap of tumor suppressor genes (TSGs) with downregulated genes in aged obese mice. Overlap p value was calculated using hypergeometric test. See also Figure S5 and Table S5.

Next, we explored the biological functions of dysregulated genes in obese mice at a later stage of life, which is equivalent to the age of a human when CRC incidence rate dramatically increases. Obesity-associated downregulated genes in aged mice are mainly related to signal transduction and transcriptional regulation (Figure 5B; Tables S5A, S5D, S5E, and S5G). We observed decreased expression of negative regulators of several signaling pathways (Table S5A), including the EGFR/RTK-RAS-ERK/MAPK cascade (*Errfi1*, *Spry1*, *Rasa4*, *Dusp1*, and *Dusp5*) (Ferby et al., 2006; Hanafusa et al., 2002; Lockyer et al., 2001; Mandl et al., 2005; Slack et al., 2001), nuclear factor κB (NF-κB) signaling (*Nfkbia*, *Nfkbiz*, and *Tnfaip3*) (Renner and Schmitz, 2009; Shem-bade et al., 2010), transforming growth factor β (TGF-β) signaling (*Smad6*, *Smad7*, and *Spsb1*) (Imamura et al., 1997; Liu et al., 2015; Nakao et al., 1997), JAK/STAT signaling (*Cish*) (Yoshimura, 1998), and mTORC1 (*Mark4*) (Li and Guan, 2013). These signaling pathways control cell proliferation, differentiation, migration, and survival; hence their dysregulations play important roles in tumor development and progression.

Normally, intracellular signal transduction is kept under tight control via negative feedback loops to ensure physiologically appropriate signaling outcomes. With downregulation of negative feedback regulators (Table S5A), the braking system of those signaling pathways were dampened in aged obese mice, potentially leading to unrestrained signaling. In parallel with this, the Jun N-terminal kinase (JNK) and p38 MAPK pathways, which are also called stress-activated protein kinase (SAPK) pathways, were likely attenuated in aged obese mice due to reduced expression of key mediators of SAPK pathways (*Map4k2*, *Map4k5*, and *Mapk12*) (Table S5A) (Chin et al., 1999; Pombo et al., 1995; Shi et al., 1999). SAPK pathways are activated by various types of environmental and pathophysiological stresses with anti-proliferative and pro-apoptotic effects. Given that the colonic epithelium is under more cell-intrinsic and -extrinsic stresses in obese mice than in control mice (Gulhane et al., 2016), the attenuation of SAPK pathways in aged obese mice might be a result of cell-autonomous adaptation to sustained stresses. Nevertheless, the insensitivity to anti-growth signals likely provides an advantage for cells to survive and proliferate under stressful conditions. In addition, downregulated genes in aged obese mice were significantly enriched with transcription factors (Table S5E), some of which are encoded by primary response genes. A total of 35 primary response genes were downregulated in aged obese mice (Figure 5C), including both immediate early genes and delayed early genes (Amit et al., 2007; Tullai et al., 2007); the majority were also downregulated in human CRC compared with normal colorectal tissue or colorectal adenoma (Figures S5A and S5B), presumably reflecting independence of mitogenic signals—a hallmark of cancer. Since delayed early genes are potential tumor suppressors (Amit et al., 2007), we investigated whether downregulated genes are enriched for tumor suppressor genes (Zhao et al., 2016). A substantial number of tumor suppressor genes were repressed in aged obese mice (Figure 5D). Collectively, downregulation of those genes in aged obese mice likely weakens proliferation barriers, thus favoring cell-autonomous growth.

Consistently, obesity-associated upregulated genes in aged mice are significantly associated with cell cycle, DNA replication, DNA repair, and chromatin organization (Figure 5A; Tables S5A, S5C, and S5F), including genes with functions in nucleosome assembly, centromeres, kinetochore organization, spindle assembly checkpoint, and chromosome condensation and segregation (Table S5C). In addition, we observed increased expression of genes involved in amino acid biosynthetic process, aminoacyl-tRNA biosynthesis, and deoxyribonucleotide biosynthetic process in aged obese mice (Tables S5A and S5C), presumably ful-filling the enhanced requirements for protein and DNA synthesis during cell proliferation. Accordingly, subunits of complex I of the respiratory chain (*Ndufa3*, *Ndufa4*, *Ndufa8*, and *Ndufa12*) and a subunit of mitochondrial ATP synthase (*Atp5g1*) were also upregulated (Table S5A), likely generating more energy to fuel the enhanced biosynthetic reactions and cell division. Collectively, upregulation of those genes may reflect increased cell proliferation of the colonic epithelium in aged obese mice compared with age-matched controls (Beyaz et al., 2016), even though no colon tumors were present in those mice.

### Reversibility of Obesity-Related Colonic Changes after Short- and Long-Term Weight Loss

**Figure 6:**
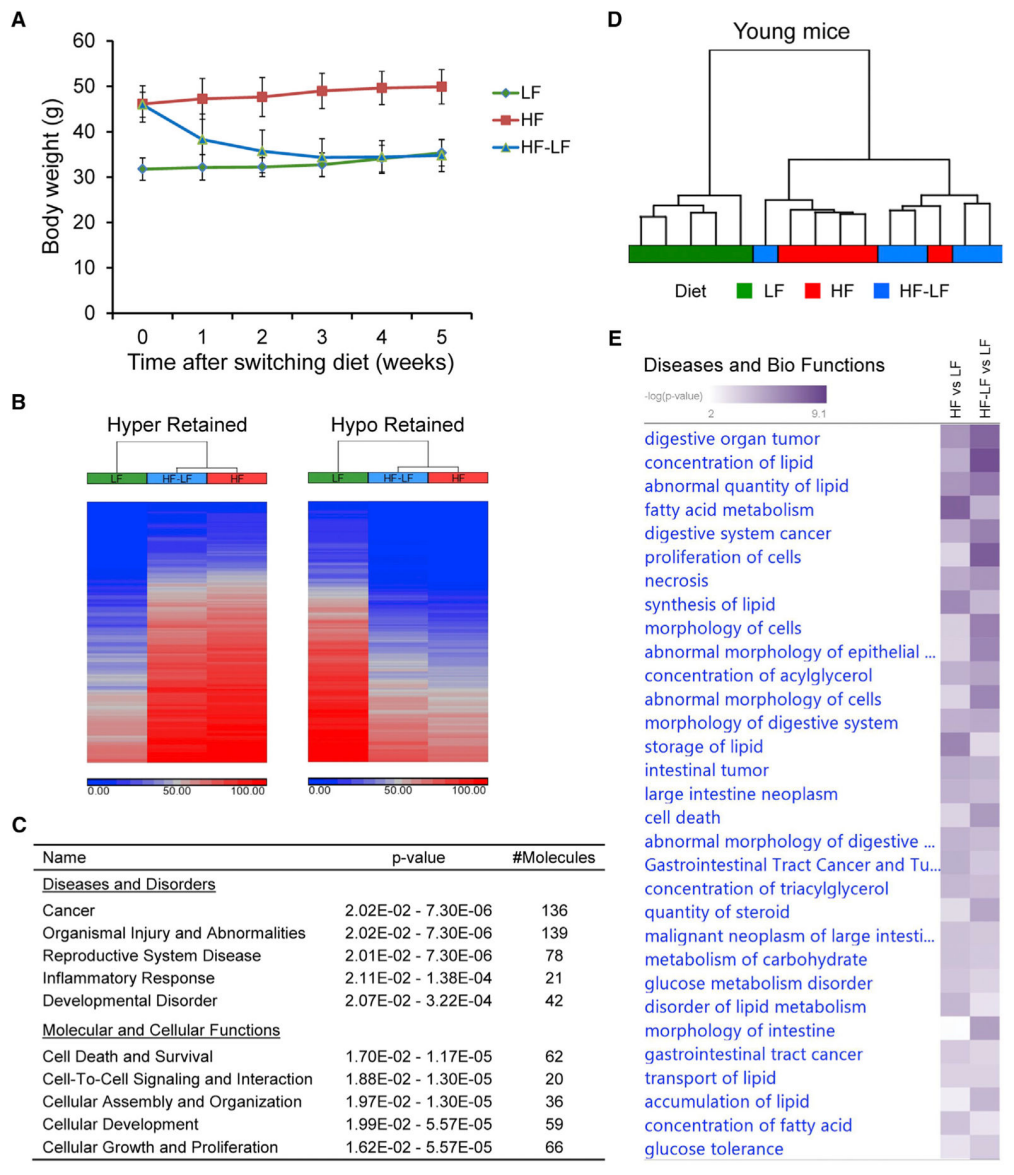
Persistent Changes in DNA Methylation and Gene Expression after Short-Term Weight Loss (A) Weekly body weight of control mice (LF), obese mice (HF), and formerly obese mice (HF-LF) after diet-switching. Data are represented as mean ± SEM (n = 5). (B) The heatmaps depict DNA methylation levels of retained DMRs in each group. Blue and red indicate unmethylated and fully methylated, respectively. (C) IPA of genes associated with retained DMRs. The top five scoring hits in each functional category are shown, together with p values and the number of retained DMR-associated genes in the enriched terms. (D) Hierarchical clustering of samples at obesity-associated dysregulated metabolic genes. Ward’s method was used with Euclidean distance calculated using standardized gene expression levels. (E) IPA comparison analysis showing the similarity between formerly obese mice and obese mice regarding the enriched diseases and biological functions. See also Figure S6 and Table S2.

To investigate whether obesity-related changes in the colonic epithelium can be reversed after weight loss, we put mice on a high-fat diet for 15 weeks to induce obesity, and then we switched them to a low-fat diet for 5 weeks to trigger weight loss. Body weights of these mice returned to normal by the end of the study (Figure 6A). We then analyzed DNA methylation and gene expression data from these formerly obese mice. We clustered obesity-related DMRs into 6 groups (C1–C6) based on their methylation levels in formerly obese mice; C1 retained methylation changes, C2 lost methylation changes, while C3 and C4 exhibited intermediate methylation levels after weight loss (Figure S6A). Among obesity-related DMRs, 1,215 hyper-DMRs and 1,040 hypo-DMRs retained methylation changes after weight loss (Figure 6B; Table S4B). Retained DMRs were associated with genes primarily involved in cancer and cellular functions pertinent to cancer, such as cell death and survival, cell-to-cell signaling and interaction, cellular development, and cellular growth and proliferation (Figure 6C). Two examples (*Hoxb13* and *Spry1*) with persistent changes after weight loss were shown (Figure S3C). Altogether, our data revealed an epigenetic memory of the previous obese state in the colonic epithelium after short-term weight loss.

We used multiple approaches to assess the similarity of gene expression changes in formerly obese mice and obese mice. First, using overlap analysis, we found that 107 obesity-associated DEGs (51 upregulated and 56 downregulated) were also significantly dysregulated in formerly obese mice in the same direction as in obese mice (Figure S6B). These commonly dysregulated genes were predominantly associated with cancer (102 of 107) and gastrointestinal disease (96 of 107), and they were significantly enriched for lipid and carbohydrate metabolism genes (Figure S6C; Table S2E). Second, we performed hierarchical clustering of samples on obesity-associated dysregulated metabolic genes. Formerly obese mice were clustered together with obese mice instead of control mice (Figure 6D), indicating that the expression levels of those genes in formerly obese mice were more close to those in obese mice than in control mice. Lastly, comparing enriched diseases and biological functions in formerly obese mice with those in obese mice, we noted that the two groups were significantly enriched with the same terms, mainly related to intestinal tumor and lipid and carbohydrate metabolism (Figures 6E and S6D; Tables S2C and S2D). Taken together, persistent changes in gene expression were observed after short-term weight loss, primarily at genes closely related to colon cancer.

Considering that those formerly obese mice just returned to normal weight after 5 weeks of diet-switching, to investigate whether obesity-related dysregulation of gene expression can be completely reversed after long-term weight loss, we performed RNA-seq in the colonic epithelium of mice with diet-switching for 28 weeks. Body weights of these mice returned to normal after 5 weeks of diet-switching, and they stayed at the same level as control mice since then (Figure S6E). Using hierarchical cluster analysis based on expression levels of obesity-related DEGs, we found that, after long-term weight loss, formerly obese mice were clustered together with control mice instead of obese mice (Figure S6F), indicating that obesity-related gene expression changes were also largely reversed.

Collectively, obesity-related changes in DNA methylation and gene expression were substantially preserved after short-term weight loss, but gene expression changes mostly went back to normal after long-term weight loss.

### Fatty Acid Metabolism Is Correlated with Clinical Outcomes of CRC Patients

The genes altered in obese mice at both life stages are involved in fatty acid metabolism (Figures S5C and S5D). The best-known metabolic perturbation in cancer cells is the Warburg effect, increased glycolysis even in the presence of oxygen, while alterations in fatty acid metabolism in cancer cells are less well studied. Therefore, we examined the changes in fatty acid metabolism in CRC. Most genes involved in short- and long-chain fatty acid oxidation exhibited significantly lower expression in CRCs than in adjacent normal tissues (Figure S7A). The heterogeneity of fatty acid metabolism among CRCs (Figure S7A) prompted us to investigate whether metabolic features of CRCs are related to clinical outcomes of CRC patients.

**Figure 7:**
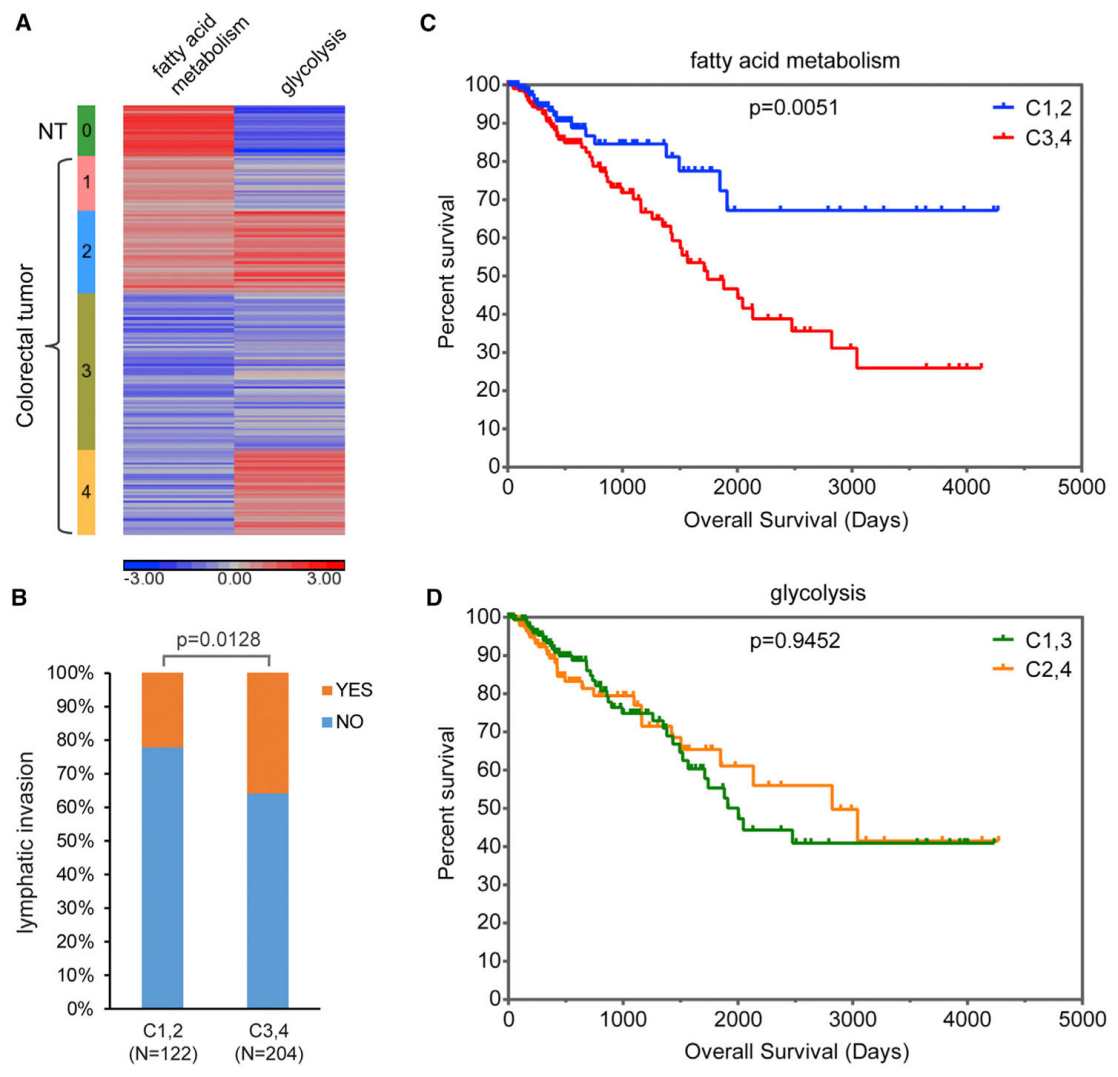
Fatty Acid Metabolism Is Associated with the Clinical Outcomes of CRC Patients (A) Stratification of CRC samples based on ssGSEA enrichment scores of two hallmark gene sets, FATTY_ACID_METABOLISM and GLYCOLYSIS. (B) The percentages of CRC patients with lymphatic invasion in the two groups defined by the fatty acid metabolism signature of their tumor samples. (C and D) Kaplan-Meier plots depict the overall survival of CRC patients stratified by either fatty acid metabolism (C) or glycolysis (D) in their tumor samples. See also Figure S7.

We characterized metabolic features of CRCs based on their changes in fatty acid metabolism and glycolysis. Single-sample gene set enrichment analysis (ssGSEA) (Barbie et al., 2009) was used to assess gene set enrichment score, which represents activation level of the corresponding biological process, for 380 primary CRCs along with 50 adjacent normal tissues from The Cancer Genome Atlas (TCGA) database. CRC samples were then stratified into four groups based on the degrees of downregulation of fatty acid metabolism and upregulation of glycolysis (Figures 7A and S7D). Decreased fatty acid metabolism was significantly associated with poor overall survival of CRC patients; however, increased glycolysis had no significant impact on patient overall survival (Figures 7C, 7D, and S7B). Moreover, CRC samples with decreased fatty acid metabolism showed more lymphatic invasion (Figures 7B and S7C). Although it is the first and most common metabolic abnormality observed in cancer, the Warburg effect might just reflect a metabolic adaptation associated with rapid cell proliferation (Figure S7E) (Shyh-Chang et al., 2013). In contrast, since tumor cells generally retain substantially the metabolic feature of corresponding normal tissue (Hu et al., 2013), the degree of fatty acid metabolism decline in CRC likely represents the extent of deviation from normal colon, thus showing better clinical relevance.

## DISCUSSION

Cellular metabolism was once thought to be a mere consequence of cellular state; it is now recognized as a key player in cell fate determination (Shyh-Chang et al., 2013). Colonic gene expression changes in young obese mice suggest a metabolic switch favoring long-chain fatty acid oxidation in the colonic epithelium (Table S2A; Figure S1C). Active fatty acid oxidation is important for the maintenance and function of normal and cancer stem cells (Carracedo et al., 2013). As butyrate, the major energy source of differentiated colonocytes, inhibits intestinal stem/progenitor cell proliferation (Kaiko et al., 2016), we reasoned that long-chain fatty acid oxidation may be essential to intestinal stem cells. Indeed, Beyaz et al. (2016) recently reported that long-chain fatty acid treatment led to the increased number and enhanced function of intestinal stem cells through the induction of PPAR-δ target genes that are involved in long-chain fatty acid oxidation. Since enforced PPAR-δ activation also augmented stemness of intestinal progenitor cells and boosted their capacity to initiate tumors (Beyaz et al., 2016), we postulate that long-chain fatty acid oxidation may play important roles in intestinal cell fate determination and intestinal tumor initiation. Therefore, our data suggest that obesity-associated colonic cellular metabolic reprogramming at young age can be an initiating event, which promotes colon tumor development by increasing the number of cells with tumorigenic potential. In addition, fatty acid metabolism was associated with clinical outcomes of CRC patients (Figures 7B and 7C). Future research is needed to elucidate the relationship between obesity and cellular metabolism in CRC.

The epigenetic machinery is highly responsive to metabolic cues, because it relies on intermediate metabolites as substrates or cofactors (Sharma and Rando, 2017). Obesity-related dysregulation of colonic cellular metabolism was accompanied by comprehensive DNA methylation changes, which were enriched at regulatory regions (Figure 2). Strikingly, these DNA methylation changes were significantly associated with future gene expression changes (Figure 4). Epigenetic priming at enhancers was observed during developmental processes (Wang et al., 2015). Presumably, it also plays roles in obesity-related pathologic processes. Hence, our data imply that, in addition to immediate effects, obesity may have latent deleterious effects, which are pre-programmed in the DNA methylome and would manifest over time, if the individual is continuously obese. Persistent obesity led to the attenuation of proliferation barriers after aging.

A striking feature of colonic gene expression changes, in aged obese mice compared with age-matched controls, is the downregulation of integral components of SAPK pathways and the downregulation of negative feedback regulators of pro-survival and pro-proliferative signaling pathways, including the EGFR/RTK-RAS-ERK/MAPK cascade, NF-κB signaling, TGF-β signaling, JAK/STAT signaling, and mTORC1 (Table S5A). These comprehensive dysregulations of signaling networks could lead to extensive changes in vital cellular processes, especially in cell proliferation and survival. In supporting this hypothesis, intestinal crypts from aged obese mice (~1 year old) exhibited niche-independent growth and better survival and regeneration after irradiation (Beyaz et al., 2016). The attenuation of feedback inhibition at multiple tiers of pro-proliferative cascades and the diminishment of anti-proliferative signaling break the balance for homeostatic regulation, rendering the cells hypersensitive to otherwise limiting amounts of growth factors and probably even capable of cell-autonomous proliferation independent of exogenous mitogenic signals, and meanwhile resistant to stress signals. Hence, we propose that cell-intrinsic rewiring of signal transduction networks in response to long-term obesity may facilitate malignant transformation of colonic epithelial cells.

Remarkably, obesity-related changes in DNA methylation and gene expression were substantially preserved after short-term weight loss, but gene expression changes were largely reversed after long-term weight loss (Figures 6 and S6), suggesting that obesity-related pathophysiological process in the colonic epithelium can be prevented with long-term weight loss. Thus, it is important for obese individuals to lose weight early.

In summary, we profiled colonic DNA methylome, transcriptome, and metabolome to identify obesity-related molecular pathophysiological changes in the colon. Those changes were not due to alterations of cell composition in the colonic epithelium, since no significant expression changes of cell-type-specific marker genes were observed in obese mice (Table S6). We provided novel mechanistic insights into how obesity increases CRC risk at different stages of life. At young age, obesity was associated with a colonic cellular metabolic switch favoring long-chain fatty acids, which were previously demonstrated to boost the numbers of intestinal stem/stem-like cells (Beyaz et al., 2016). Consequently, the chance is increased for an obese individual to gain oncogenic mutations in colonic stem cells. After aging, obesity was associated with decreased expression of tumor suppressor genes and negative feedback regulators of pro-survival and pro-proliferation signaling pathways. These changes prime for unrestrained signaling to accelerate the initiation and progression of colon tumorigenesis once oncogenic events occur. It should be noted that, without additional oncogenic driving force, obesity-related colonic molecular changes unlikely lead to colon cancer. Since the mice in our study were raised in a specific pathogen-free environment, no colon tumors were observed at the end of the study. However, obesity-related colonic molecular changes increase the odds of colon cancer development and progression, if they co-occur with oncogenic insults. Therefore, the lifetime risk of CRC increases in the obese.

Although colon tumors occur less frequently than small intestine tumors in mice unlike humans (Newmark et al., 2009), we used mouse colon as a proxy of human colon due to their similarities in tissue structure, cell composition, and physiological functions. One limitation of our study is that only male mice were used. Since female obese mice exhibited similar patterns of changes as male obese mice in colonic enhancer landscape and intestinal stem/progenitor cell functions (Beyaz et al., 2016; Li et al., 2014), we predict that it is also the case for DNA methylation and gene expression changes.

## EXPERIMENTAL PROCEDURES

### Mice

Six-week-old male C57BL/6J mice were fed either a low-fat diet (10% fat diet, D12450B; Research Diets) or a high-fat diet (60% fat diet, D12492; Research Diets). The source of fat in the diets is lard. Specifically, five mice were put on each dietary regimen as follows: (1) low-fat diet for 20 (or 43) weeks; (2) high-fat diet for 20 (or 43) weeks; and (3) high-fat diet for an initial 15 weeks and then switched to a low-fat diet for another 5 (or 28) weeks. The mice were housed in a specific pathogen-free facility. Body weight was measured weekly after diet-switching. At the end of the study, the mice were humanely euthanized and the colons were collected for further experiments. All animal experiments were approved by the NIEHS Animal Care and Use Committee, and they were performed according to NIH guidelines for care and use of laboratory animals.

### Isolation of the Colonic Epithelium and DNA/RNA Preparation

The colonic epithelium was isolated as previously described (Li et al., 2014). Genomic DNA and total RNA were extracted from the cells using QIAGEN AllPrep DNA/RNA/miRNA Universal Kit. See also the Supplemental Experimental Procedures.

### mRNA Sequencing and Data Analysis

mRNA sequencing libraries were prepared using the TruSeq Stranded mRNA Sample Prep Kit (Illumina) and sequenced on HiSeq 2000 (paired-end 50 bp). Sequencing reads were mapped against mm10 reference genome using TopHat (Trapnell et al., 2009). Mapped read counts per annotated gene were collected with HTSeq-Count (Anders et al., 2015). DESeq2 (Love et al., 2014) was then used to identify DEGs (|fold change| > 1.2, p < 0.01, and adjusted p value < 0.25). See also the Supplemental Experimental Procedures.

### WGBS and Data Processing

Genomic DNA was sonicated to an average size of 200 bp using a Covaris S220 instrument. DNA fragments were end-repaired, adenylated, and ligated to Illumina-compatible adaptors using BIOO NEXTflex Bisulfite-Seq Kit. Bisulfite conversion was performed using EZ DNA Methylation-Lightning Kit (Zymo Research). PCR was then carried out to enrich bisulfite-converted and adaptor-ligated fragments. The libraries were sequenced on Nextseq 500 (paired-end 75 bp). According to the coverage recommendations for WGBS (Ziller et al., 2015), we sequenced five biological replicates per group and achieved an average coverage >5× per sample.

Sequencing reads were mapped to mm10 reference genome via Bismark (Krueger and Andrews, 2011) with Bowtie as the underlying alignment tool. Duplicates were removed, and any redundant mapped bases due to overlapping mates from the same read pair were trimmed. Furthermore, read cycles showing methylation bias on M-bias plot were trimmed from each mapped hit. The observed bisulfite conversion rate was >99%. See also the Supplemental Experimental Procedures.

### DMR Detection

Rao Scott Likelihood Ratio Test (Rao and Scott, 1981, 1987) was used to identify differentially methylated CpG sites. CpGs were considered differentially methylated only if they had an RSLRT p value < 0.05, absolute methylation difference >10%, and total weighted coverage ≥10. Next, differentially methylated CpGs within 5,000 bp were merged into discrete regions. Finally, differential methylation analysis was repeated on the region level using RSLRT. Differentially methylated regions were defined as having a Benjamini-Hochberg adjusted DMR level RSLRT p value < 0.01, absolute methylation difference >30% or fold change >5, and containing at least 2 differentially methylated CpGs. See also the Supplemental Experimental Procedures.

### Ribo-Zero RNA-Seq and Data Analysis

RNA-seq libraries were prepared using TruSeq Stranded Total RNA Library Prep Kit with Ribo-Zero H/M/R Gold (Illumina) and sequenced on Illumina NextSeq 500 (paired-end 76 bp). Read pairs were filtered by a mean base quality score >20, followed by adaptor-trimming with CutAdapt, and then they were mapped to mm10 reference genome with spliced transcripts alignment to a reference (STAR) (Dobin et al., 2013). Mapped hits per gene was calculated with Subread featureCounts. DEGs were identified by DESeq2 (Love et al., 2014) with a cutoff of padj < 0.05 and |FC| > 1.2. See also the Supplemental Experimental Procedures.

### Statistics

Data analysis was performed using Partek and GraphPad Prism. The heat-maps were prepared using Partek. Overlap significance was calculated using hypergeometric test. Body weights and gene expression data were represented as mean ± SEM.

## Supplementary Material

1

2

3

4

5

6

7

8

## Highlights

- Obesity reprograms mouse colonic DNA methylome, leading to gene expression changes
- Obesity triggers a metabolic switch favoring long-chain fatty acids at young age
- Obesity induces a tumor-prone gene signature in the colonic epithelium after aging
- Obesity-related changes are reversed after long-term, but not short-term, weight loss
