# Supplementary material for: Transcriptome and DNA Methylome Analysis in a Mouse Model of Diet-Induced Obesity Predicts Increased Risk of Colorectal Cancer
Source: Cell Rep. Author manuscript; Available in PMC 2018 Feb 1. (PMC5793878; doi:10.1016/j.celrep.2017.12.071)
Supplement: 1 [file NIHMS937389-supplement-1.pdf]

Cell Reports, Volume *22*

Supplemental Information

Transcriptome and DNA Methylome Analysis in a Mouse Model of Diet-Induced Obesity Predicts Increased Risk of Colorectal Cancer

Ruifang Li, Sara A. Grimm, Deepak Mav, Haiwei Gu, Danijel Djukovic, Ruchir Shah, B. Alex Merrick, Daniel Raftery, and Paul A. Wade

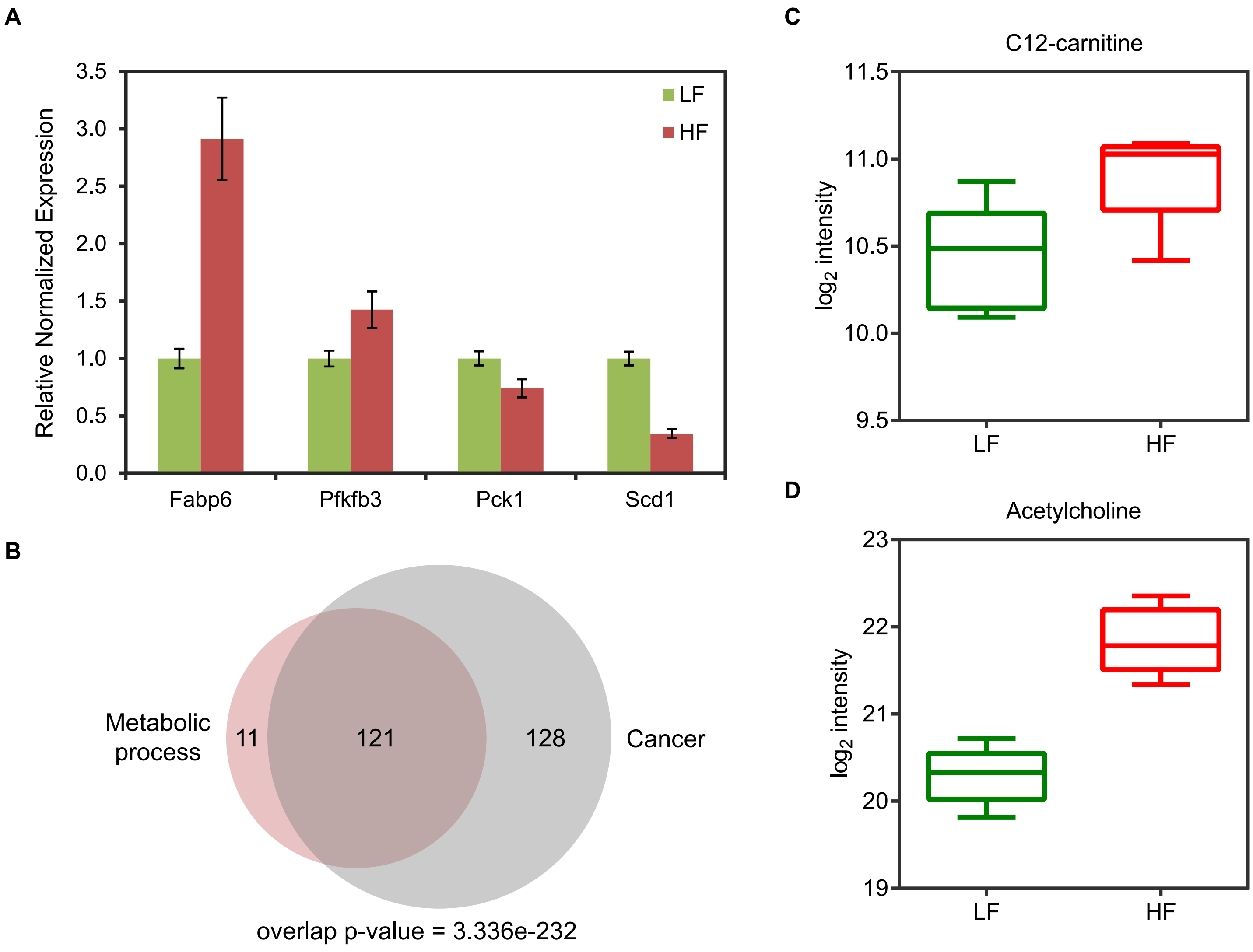

Figure S1. Differentially expressed metabolic genes and altered metabolites in colonic epithelium of young obese mice, related to Figure 1. (A) Validation of RNA-Seq data using real-time RT-PCR. Gene expression levels were normalized to that of Gapdh. Data are represented as mean ± SEM (n=5). (B) Overlap of dysregulated genes involved in metabolic processes with dysregulated cancer-related genes in young obese mice. Overlap p-value was calculated using hypergeometric test. (C and D) Levels of C12-carnitine and acetylcholine in colonic epithelium from young obese and control mice. Y-axis indicates the area of peak from targeted metabolome analysis.

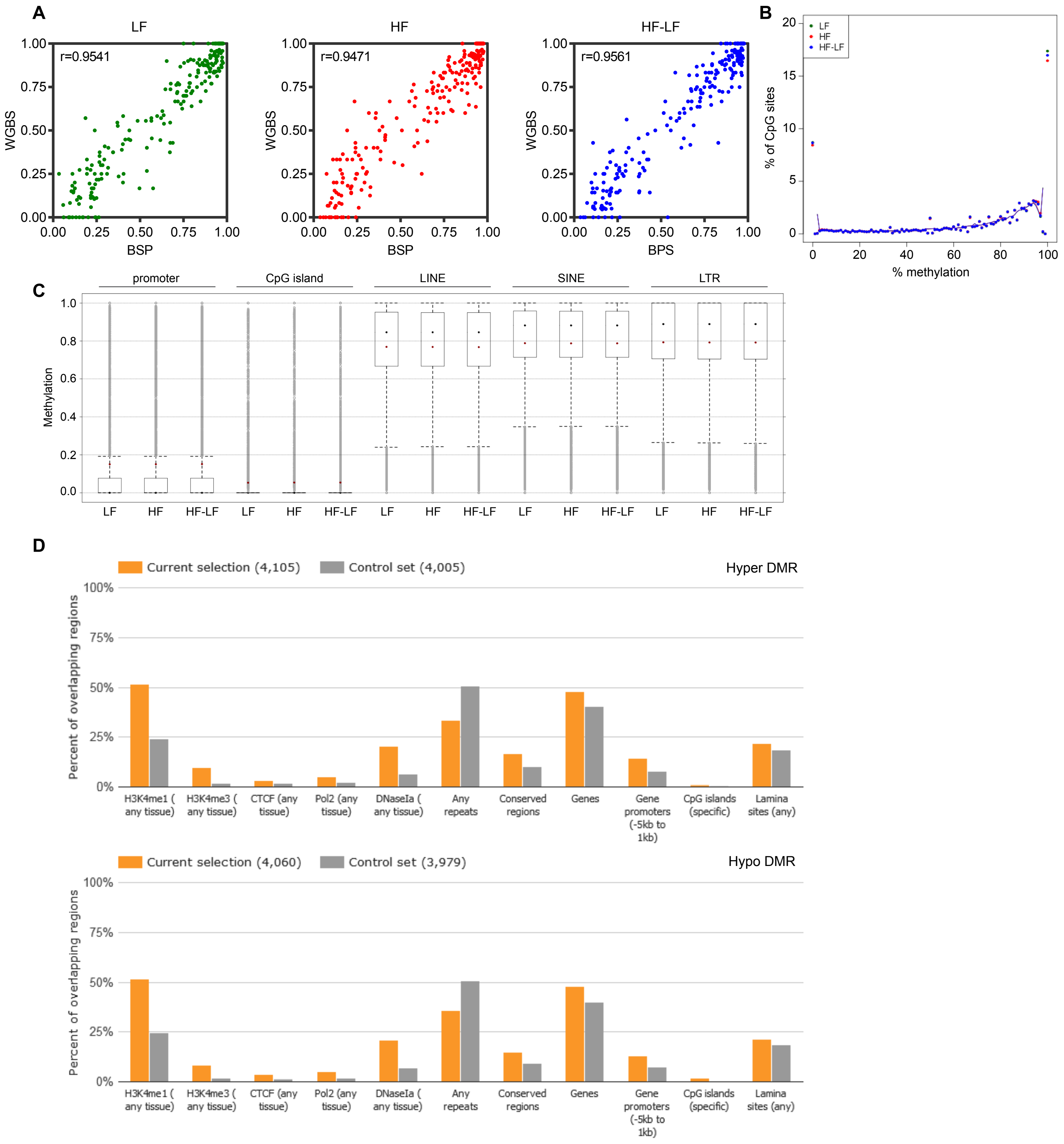

Figure S2, related to Figure 2. (A) WGBS and BSP-Seq showed strong correlation of the methylation levels of 233 randomly selected CpG sites. (B) Bimodal distribution of DNA methylation at individual CpG sites. (C) Global methylation levels of promoters, CpG islands, and repetitive elements (LINE, SINE, and LTR). The blue and red dots represent the median and the mean, respectively. (D) Percentage of DMRs or matched control regions overlapping with annotated genomic features and experimentally defined functional genomic elements. Upper panel: hyper DMRs; lower panel: hypo DMRs.

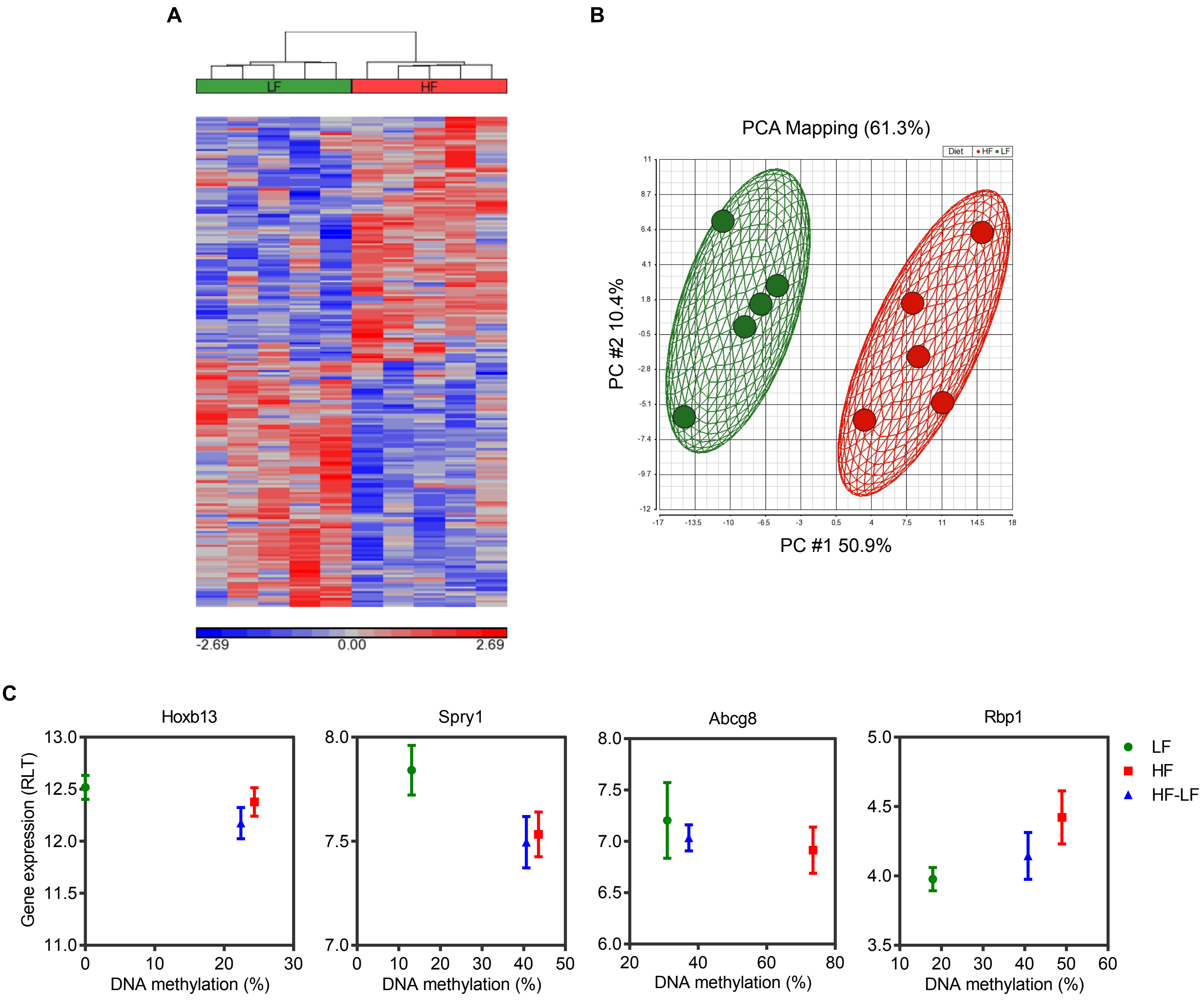

Figure S3. DMR-target genes without significant differential expression, related to Figure 3. (A) Heatmap depicts the standardized expression levels of 213 non-DEG-overlapping DMR-target genes in control mice (LF) and obese mice (HF). (B) Obese mice and control mice were well separated by Principal Components Analysis (PCA), based on expression levels of 213 non-DEG-overlapping DMR-target genes. (C) Examples of correlation between DNA methylation and gene expression.

Figure S4

A

	Consensus binding sites	p-value (DMR motif enrichment)	p-value (DEG upstream regulator)
E2F4	GGCGGGAAAH	1.00E-08	2.14E-19
E2f	TTSGCGCGAAAA	1.00E-03	2.23E-08
ELK4	NRYTTCCGGY	1.00E-13	2.62E-10
ELK1	HACTTCCGGY	1.00E-06	1.62E-05

B

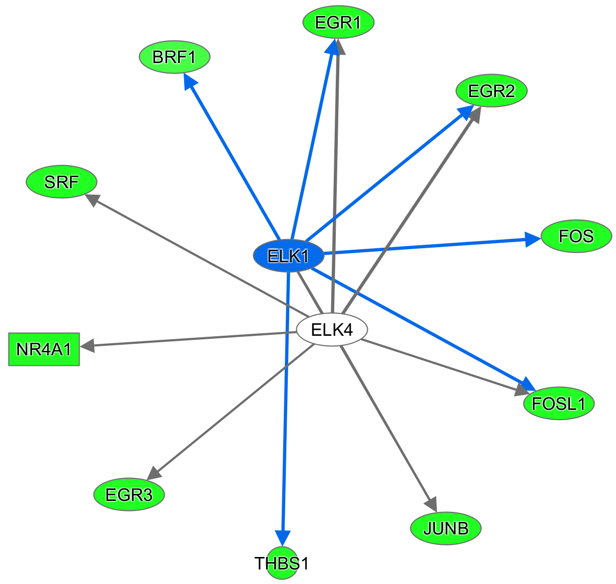

C

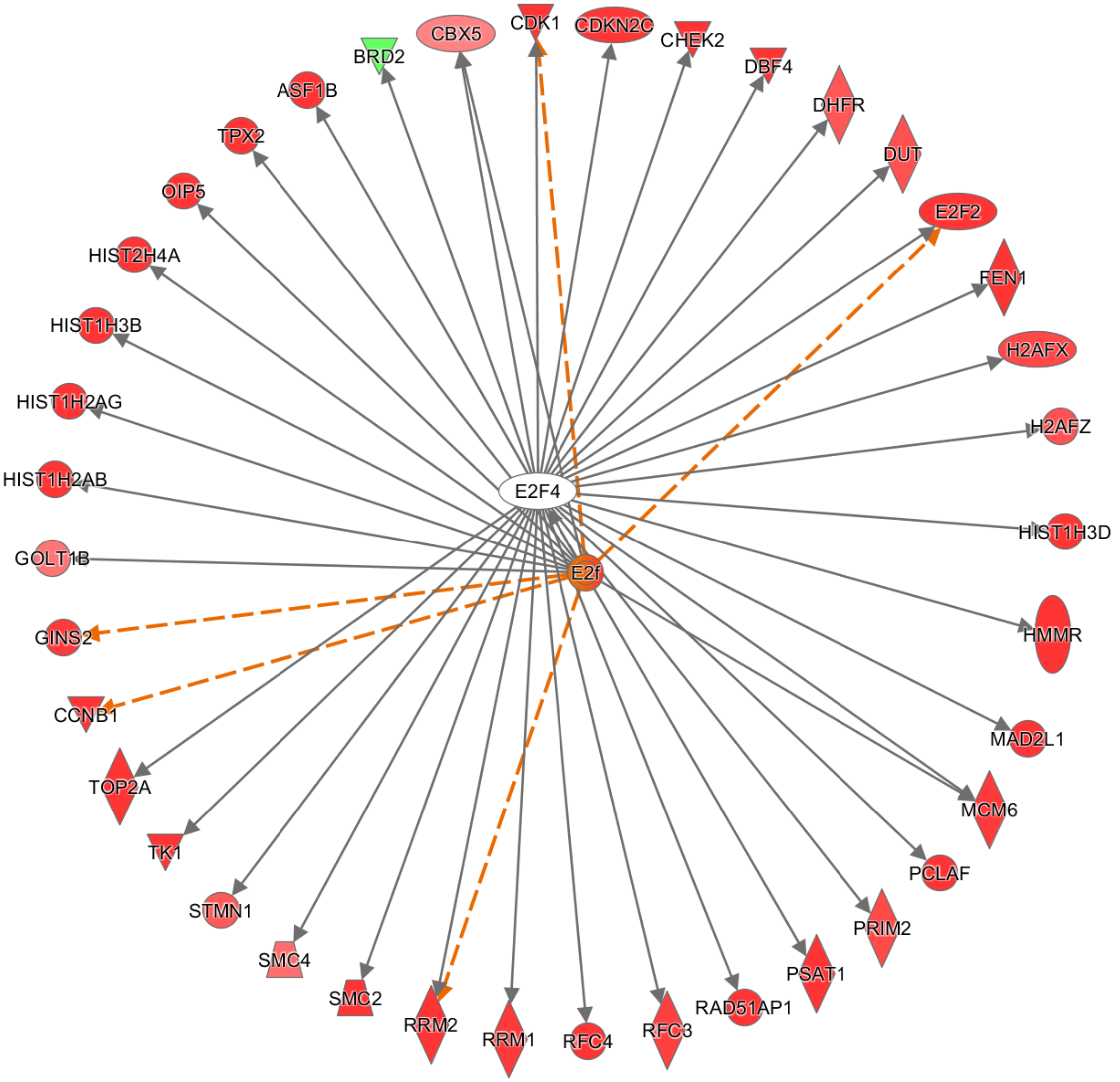

Figure S4. Examples of transcription factors with binding motifs enriched at DMRs and also serving as upstream regulators of DEGs in aged obese mice, related to Figure 4. (A) The p-values from HOMER motif enrichment analysis and IPA upstream regulator analysis were shown for the following transcription factors: E2F, ELK1, and ELK4. (B and C) Differential expression of the target genes of E2F, ELK1 and ELK4. Red indicates up-regulation while Green indicates down-regulation of genes in aged obese mice relative to age-matched controls.

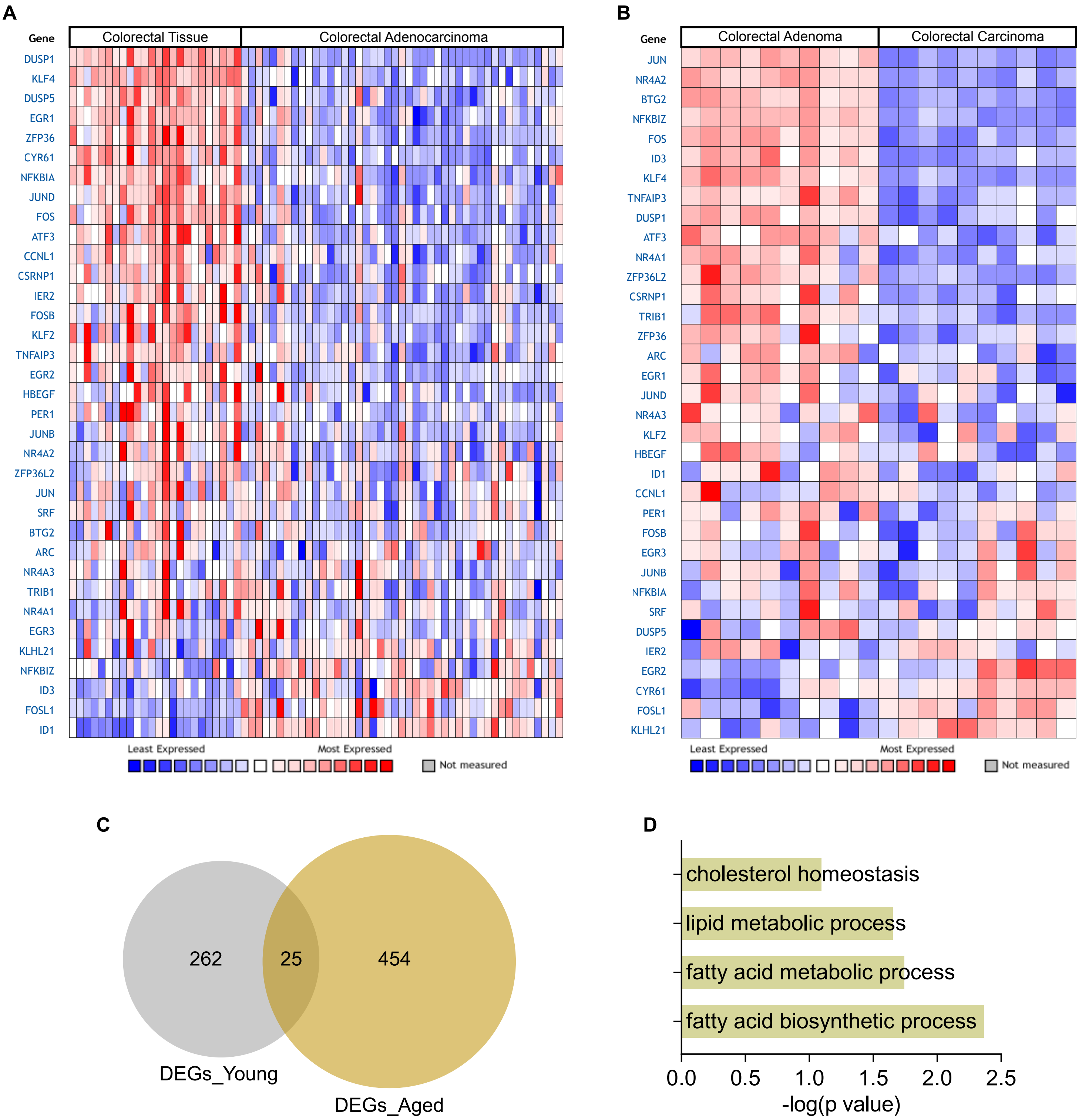

Figure S5, related to Figure 5. (A and B) Heatmaps display relative expression of the 35 primary response genes in human colorectal cancer compared with normal colorectal tissue (A) and benign colorectal tumor (B). (C) Overlap of obesity-related DEGs in young and aged mice. (D) GO_BP terms enriched with consistently altered genes in both young and aged obese mice.

Figure S6

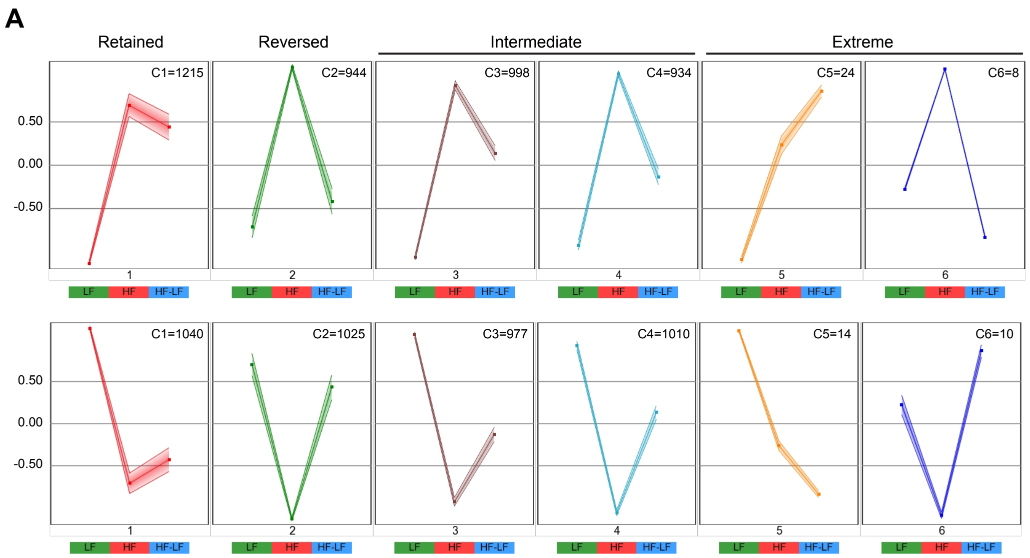

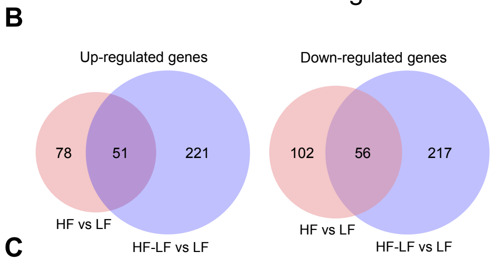

C

Name	p-value	#Molecules
Diseases and Disorders		
Cancer	1.45E-02 - 2.76E-09	102
Gastrointestinal Disease	1.50E-02 - 2.76E-09	96
Organismal Injury and Abnormalities	1.50E-02 - 2.76E-09	102
Developmental Disorder	1.50E-02 - 2.51E-05	15
Hereditary Disorder	1.50E-02 - 2.51E-05	27
Molecular and Cellular Functions		
Lipid Metabolism	1.50E-02 - 1.01E-06	31
Small Molecule Biochemistry	1.50E-02 - 1.01E-06	38
Molecular Transport	1.50E-02 - 1.72E-04	29
Carbohydrate Metabolism	1.50E-02 - 2.33E-04	17
Cell Morphology	1.50E-02 - 4.97E-04	29

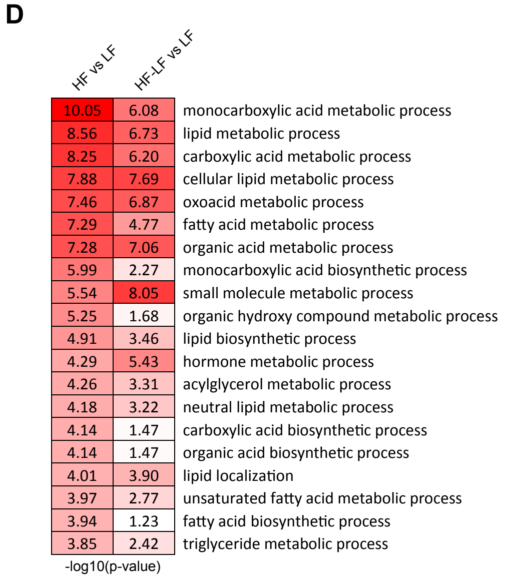

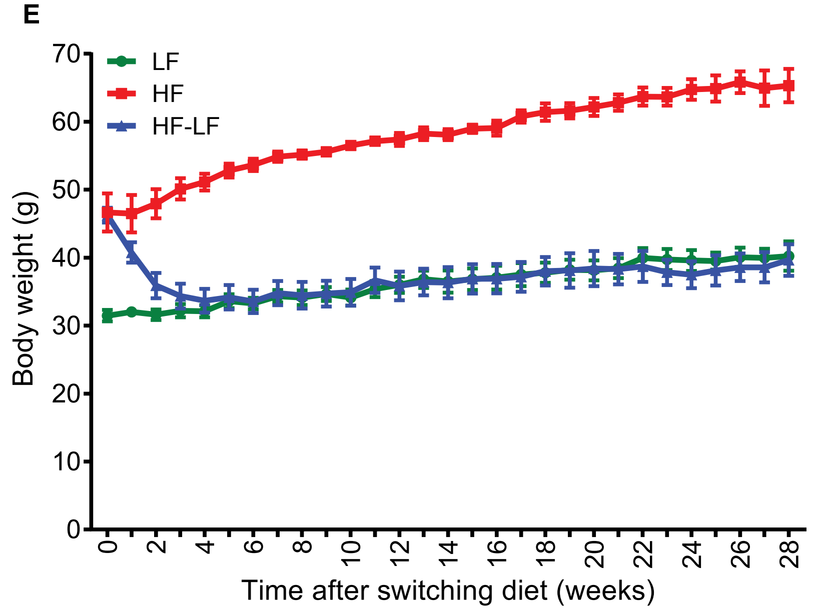

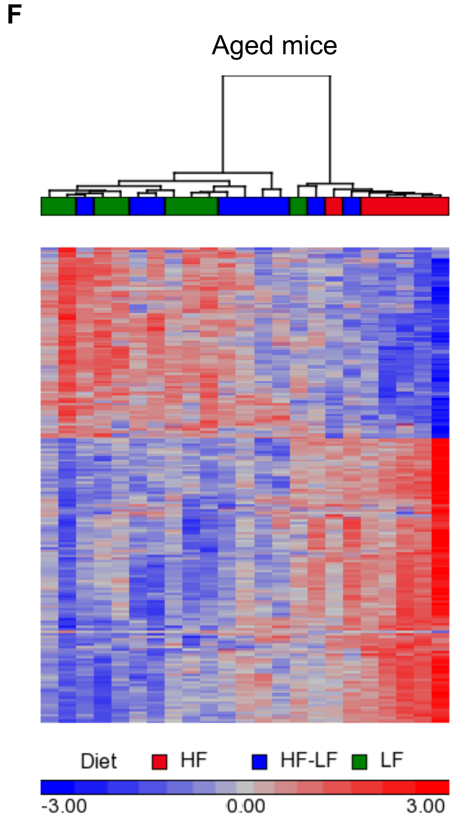

Figure S6. Obesity-related changes in DNA methylation and gene expression were substantially preserved after short-term weight loss but were largely reversed after long-term weight loss, related to Figure 6. (A) Obesity-associated DMRs were clustered into 6 groups based on their methylation levels in formerly obese mice (short-term). The number of DMRs in each group was shown in the upper-right corner. (B) Overlap of DEGs in formerly obese mice (short-term) with DEGs in obese mice. (C) Ingenuity Pathway Analysis of commonly dysregulated genes. The top five scoring hits in each functional category are shown, together with p-values and the number of commonly dysregulated genes in enriched terms. (D) Comparison of enriched GO terms in formerly obese mice (short-term) with that in obese mice. Colors indicate the significance of p-values with darker red being more significant and white being non-significant. The numbers are -log10 of p values. (E) Weekly body weight of control (LF), obese (HF), and formerly obese (HF-LF) mice from the long-term weight loss study (diet-switching for 28 weeks). Data are represented as mean ± SEM (n=4 or 5). (F) Hierarchical clustering of samples on obesity-related differentially expressed genes in aged mice. Ward's method was used with Pearson’s Dissimilarity calculated using standardized gene expression levels.

Figure S7

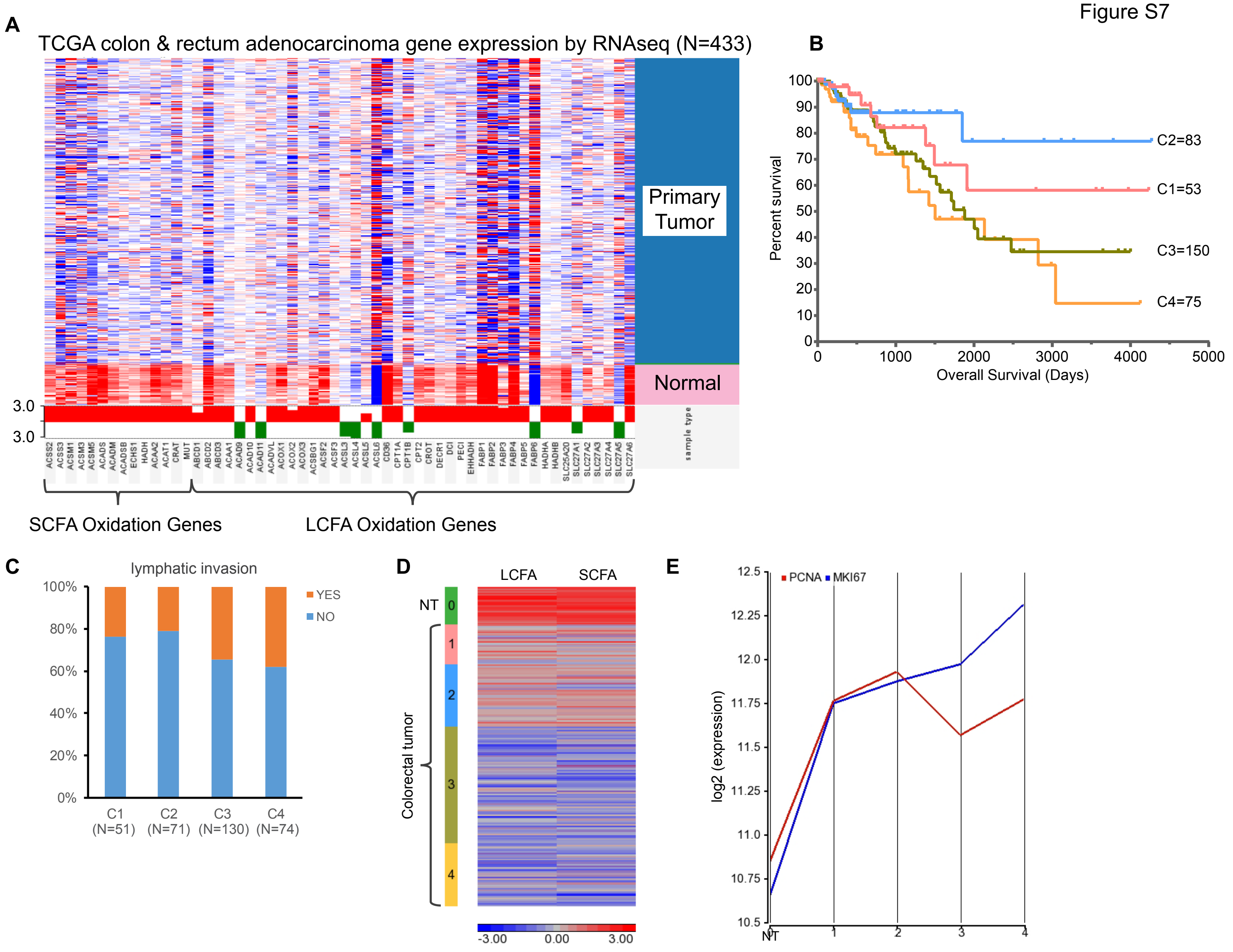

Figure S7, related to Figure 7. (A) Heatmap displays normalized expression of short- and long-chain fatty acid oxidation genes in primary colorectal cancer and adjacent normal tissue. Red correlates with higher expression. The statistical track, which is displayed under the gene heatmap, shows the -log (p-value) for each gene comparing primary colorectal cancer with adjacent normal tissue. (B) Kaplan-Meier plot depicts the overall survival of CRC patients stratified by the metabolic features of their tumor samples. (C) The percentages of CRC patients in the four defined groups harboring lymphatic invasion. (D) Heatmap displays normalized ssGSEA enrichment scores of gene sets specific for SCFA and LCFA metabolism. (E) Average expression levels of PCNA and MKI67 in CRC samples of each group.

Supplemental Experimental Procedures

Isolation of Colonic Epithelium

Colonic epithelium was isolated as previously described (Li et al., 2014). Briefly, the entire colon was removed from each mouse, flushed with PBS without calcium and magnesium, and then cut open longitudinally. After being washed two more times, the colon was placed in PBS with 5 mM EDTA to incubate at 37 °C for 15 min on a rotator. Finally, the colon tissue was removed and colonic epithelium was pelleted and washed twice with PBS. The cells were used immediately or snap frozen in liquid nitrogen and stored at -80 °C.

DNA and RNA Preparation

Genomic DNA and total RNA were isolated using the Qiagen AllPrep DNA/RNA/miRNA Universal Kit according to the manufacturer's instructions. The concentrations of DNA and RNA were measured using the NanoDrop spectrophotometer. The quality of DNA and RNA was assessed by agarose gel electrophoresis and Bioanalyzer, respectively.

Real-Time RT-PCR

cDNA was generated using the iScript™ cDNA Synthesis Kit (Biorad, catalog #170-8890). Relative expression levels of Fabp6, Pfkfb3, Pck1, and Scd1 were determined by real-time PCR assays using TaqMan® Gene Expression Assay kits (Applied Biosystems). Gene expression levels were normalized to that of Gapdh.

mRNA Sequencing and Data Analysis

mRNA sequencing libraries were prepared using the TruSeq Stranded mRNA Sample Prep Kit (Illumina, #RS-122-2103). Starting with 100 ng of total RNA, polyadenylated RNA (primarily mRNA) was selected and purified using oligo-dT conjugated magnetic beads. The mRNA was then chemically fragmented and converted into single-stranded cDNA using reverse transcriptase and random hexamer primers, with the addition of Actinomycin D to suppress DNA-dependent synthesis. Double-stranded cDNA was created by removing the RNA template and synthesizing the second strand in the presence of dUTP in place of dTTP. A single 'A' nucleotide was added to the 3' end of double-stranded cDNA to facilitate ligation of sequencing adapters, which contain a single 'T' nucleotide overhang. Adapter-ligated cDNA was amplified using polymerase chain reaction (PCR) to increase the amount of sequence-ready library. During PCR amplification, the polymerase stalls when it encounters a uracil, rendering the second strand a poor template. Accordingly, only the first strand was used as a template, thereby preserving the strand information. Final cDNA libraries were analyzed for size distribution using Agilent Bioanalyzer (DNA 1000 kit, Agilent # 5067-1504), quantitated by qPCR (KAPA Library Quant Kit, KAPA Biosystems # KK4824), and then normalized to 2 nM prior to sequencing on HiSeq 2000 (paired-end 50 bp).

General quality control checks were performed with FastQC v0.14.0 (http://www.bioinformatics.babraham.ac.uk/projects/fastqc/). Each dataset was filtered to retain only sequences for which both reads in a pair had an average base quality score of at least 20. Filtered datasets were mapped against the mm10 reference genome using TopHat (Trapnell et al., 2009) v2.0.4 (parameters --b2-sensitive --library-type fr-firststrand -g 10 --mate-inner-dist 40 --mate-std-dev 50). Mapped read counts per annotated gene were collected with HTSeq-Count (Anders et al., 2015); the gene models were generated from RefSeq annotations downloaded from the UCSC Genome Browser as of April 6, 2014. DESeq2 (Love et al., 2014) v1.2.10 was then used to identify differentially expressed genes ($|\text{fold change}| > 1.2$, $p < 0.01$, and adjusted p-value < 0.25).

Bisulfite PCR Deep Sequencing

Bisulfite PCR primers were designed using website: http://www.urogene.org/methprimer/. Genomic DNA was bisulfite converted using EZ DNA Methylation-Lightning™ Kit (Zymo Research Corporation) before PCR amplification. Amplicons from a single sample were pooled and individually indexed using TruSeq™ RNA Sample Preparation kit (Illumina) to create a multiplex library prior to sequencing (250 bp PE) on an Illumina MiSeq instrument using v3 chemistry.

WGBS Data Processing

General quality control checks were performed with FastQC (http://www.bioinformatics.babraham.ac.uk/projects/fastqc/). Filtered sequencing datasets were mapped to a reference genome via Bismark (Krueger and Andrews, 2011) v0.14.0 (parameters -X 10000 --non_bs_mm -n 2 -l 50 -e 70 --chunkmbs 1024), using Bowtie (Langmead et al., 2009) v0.12.8 as the underlying alignment tool. The reference genome contains the genome sequence of Enterobacteria phage λ in addition to all chromosomes of the mm10 assembly (GRCm38). Mappings for all datasets generated from the same library were merged, and duplicates were removed via the deduplication tool included in the Bismark package. Mapped reads were then separated by genome (mouse or phage λ) and by source strand (plus or minus). Any redundant mapped bases due to overlapping mates from the same read pair were trimmed to avoid bias in quantification of methylation status. Furthermore, read cycles where methylation bias was observed (typically at the 5' end and sometimes also at the 3' end of reads) were trimmed from each mapped hit. Precise boundaries of the trim positions were gleaned from the M-bias plot generated by Bismark's bismark_methylation_extractor tool; the M-bias plot for each sample/sequencing run combination was evaluated independently. Read pairs mapped to phage λ were used as a QC assessment to confirm that the observed bisulfite conversion rate was > 99%. Read pairs mapped to the mouse reference genome were used for downstream analysis.

DMR Detection

Pairwise comparison was first carried out on a single CpG level. For each CpG in the mouse genome (mm10), we obtained methylated and unmethylated cytosine counts from the aligned WGBS data and performed Rao Scott Likelihood Ratio Test to identify statistically significant differential methylation. This statistical test, typically used for association analysis in complex sampling survey analysis (Rao and Scott, 1981, 1984, 1987), is simply a design-corrected version of the standard likelihood ratio test used in multinomial contingency tables, but it allows design-based reweighting of the observed counts. Compared to alternative parametric techniques designed for over-dispersed data such as beta-binomial regression, this test is not computationally intensive, making it feasible to use with the millions of CpGs resulting from whole genome studies. The sampling weight for each replicate was computed using the total number of methylated/unmethylated base counts across all CpG dinucleotides. CpGs were considered differentially methylated only if RSLRT P-value < 0.05, absolute methylation difference > 10%, and total weighted coverage ≥ 10 in at least one cohort. Subsequently, differentially methylated CpGs within 5000 bp were merged into discrete regions. Next, differential methylation analysis was repeated on the region level using RSLRT. Differentially methylated regions were defined as having a Benjamini-Hochberg adjusted DMR level RSLRT P-value less than 0.01, absolute methylation difference more than 30% or fold change more than 5, and containing at least 2 differentially methylated CpGs.

BETA Analysis and functional annotation of DMRs

Binding and expression target analysis (BETA) (Wang et al., 2013) was run on the web server (http://cistrome.org/ap/) using default parameters. Overlap of DMRs with annotated genomic features and experimentally defined functional genomic elements was performed using EpiExplorer (Halachev et al., 2012), which is a web tool allowing users to explore large-scale genomic datasets in search of interesting functional associations with user-defined regions. When custom region set was uploaded, EpiExplorer automatically generated randomized control regions, simply by reshuffling the genomic positions of all regions in the user-uploaded dataset. The randomized control set was automatically included as a reference (in grey) in all bar charts to assess whether the association between custom region set and an annotation attribute is biologically relevant. Genomic coordinates of DMRs were converted from mm10 to mm9 by CrossMap (Zhao et al., 2014), since EpiExplorer currently supports only mm9. The selected overlap criterion is any overlap. The results did not change with at least 10% overlap or at least 50% overlap.

HOMER Motif Analysis

Each individual CpG site within DMRs was extracted and extended on both sides for 10bp; the resulting regions were subsequently merged, if there was any overlap. The HOMER (v4.9.1) motif analysis tool (Heinz et al., 2010) was used to identify known transcription factor motifs enriched at the query sequences relative to a size and GC-matched genomic background.

Untargeted Lipidomics Analysis

After 100 µL 1x PBS was added to the cell pellets, the mixture was sonicated in an ice/water bath for 15 min. Then 500 µL chloroform:methanol (2:1, V:V) was added and the mixture was incubated on dry ice for 30 min. After centrifuging at 14,000 RPM for 5 min, the lower phase was collected for lipidomics analysis. The lipidomics data were collected using a standard metabolic profiling MS method in the NW-MRC (Buas et al., 2016). LC-QTOF-MS experiments were performed using an Agilent 1200 SL LC system coupled online with an Agilent 6520 Q-TOF mass spectrometer (Agilent Technologies, Santa Clara, CA). Each prepared sample (8 µL for positive ESI ionization, 12 µL for negative ESI ionization) was injected onto an Agilent Zorbax 300 SB-C8 column (2.1× 50mm, 1.8-µm), which was heated to 50 °C. The flow rate was 0.4 mL/min. Mobile phase A was 5 mM ammonium acetate and 0.1% formic acid in water, and mobile phase B was 5% water in ACN containing 5 mM ammonium acetate and 0.1% formic acid. The mobile phase composition was kept isocratic at 35% B for 1 min, and was increased to 95% B in 19 min; after another 10 min at 95% B, the mobile phase composition was returned to 35% B. The ESI voltage was 3.8 kV. The Q-TOF MS spectrometer was calibrated prior to each batch run and a reference channel infusing the standard reference mixture (G1969-85001, Agilent Technologies, Santa Clara, CA) was used during the experiments to ensure mass accuracy. The mass scan range was 100–1600 Da, and the acquisition rate was 1.5 spectra/s. The Q-TOF data were extracted using Agilent MassHunter Qualitative Analysis (version B.07.00) and Mass Profiler Professional (MPP, version B.13.00) software. The absolute intensity threshold for the LC–Q-TOF data extraction was 1000, and the mass accuracy limit was set to 10 ppm.

Targeted Metabolomics Analysis

We used methanol:H_2O (8:2) to extract aqueous metabolites. The LC-MS/MS data were collected using a standard targeted metabolic profiling MS method developed in the NW-MRC that has been used in a growing number of studies (Carroll et al., 2015; Gu et al., 2015; Reyes et al., 2014; Sood et al., 2015; Sperber et al., 2015). Briefly, the LC-MS/MS experiments were performed on an Agilent 1260 LC (Agilent Technologies, Santa Clara, CA) AB Sciex QTrap 5500 MS (AB Sciex, Toronto, Canada) system. Each sample was injected twice, 15 µL for analysis using negative ionization mode and 5 µL for analysis using positive ionization mode. Both chromatographic separations were performed using hydrophilic interaction chromatography (HILIC) on the Waters XBridge BEH Amide column (150 x 2.1 mm, 2.5 µm particle size, Waters Corporation, Milford, MA). The flow rate was 0.3 mL/min. The mobile phase was composed of Solvents A (10 mM ammonium acetate in 90% H2O/ 5% acetonitrile/ 5% methanol + 0.3% acetic acid) and B (10 mM ammonium acetate in 85% acetonitrile/ 10% H2O / 5% methanol+ 0.3% acetic acid). After the initial 1.5 min isocratic elution of 90% B, the percentage of Solvent B was decreased to 45% at t=5 min. The composition of Solvent B was maintained at 45% for 5 min (t=10 min), and then the percentage of B was gradually increased to 90%, to prepare for the next injection. The metabolite identities were confirmed by spiking the pooled serum sample used for method development with mixtures of standard compounds. The extracted MRM peaks were integrated using MultiQuant 2.1 software (AB Sciex, Toronto, Canada).

Metabolomics Data Analysis

Targeted metabolomics and untargeted lipidomics datasets were first filtered by applying the 80% rule to keep only features present in most samples; the retained features were further filtered by variations between QC samples and among biological replicates (CV% of QCs <= 30% or CV% of biological replicates < 50%). Missing values were replaced with the median of the group. Finally, the data were log2 transformed and two-sample t-test was performed to detect differential metabolites.

Ribo-Zero RNA-Seq and Data Analysis

RNA sequencing libraries were prepared using the TruSeq Stranded Total RNA Library Prep Kit with Ribo-Zero H/M/R Gold, following the manufacturer's instructions. The libraries were sequenced as paired-end 76-mers on an Illumina NextSeq 500. Read pairs were filtered based on a mean base quality score more than 20, followed by adapter-trimming with CutAdapt (v1.2.1) (parameters –O 5 –q 0). Filtered and trimmed read pairs were mapped to the mm10 reference genome with STAR (Dobin et al., 2013) (v2.5) (parameters --outMultimapperOrder Random --outSAMattrIHstart 0 --outFilterType BySJout --alignSJoverhangMin 8 --limitBAMsortRAM 55000000000). Quantification of mapped hits per gene was calculated with Subread featureCounts v1.5.0-p1 (parameters -s2 -Sfr). Differentially expressed genes were identified by DESeq2 (Love et al., 2014) (v1.10.1) with a cutoff of padj<0.05 and |FC|>1.2. The RefSeq gene models downloaded from the UCSC Genome Browser on April 11, 2014, were used with gene Rn45s excluded for the analysis.

Functional Annotation of Obesity-Related Differentially Expressed Genes

VLAD (Visual Annotation Display; http://proto.informatics.jax.org/prototypes/vlad/) is a tool for visualizing GO annotations. VLAD (Version 1.5.1) was run with default parameters.

IPA core analysis was performed to interpret gene sets in the context of diseases and bio functions (molecular and cellular functions). To compare changes in biological states across conditions, IPA comparison analysis was performed on two core analyses, HF vs LF and HF-LF vs LF.

Reactome pathway enrichment analysis (Fabregat et al., 2016) was performed to determine which events (pathways and/or reactions) are statistically enriched in obesity-related differentially expressed genes. Reactome (http://reactome.org) is a free, open-source, curated and peer-reviewed knowledge-base of biomolecular pathways. Pathways in Reactome are organized hierarchically, grouping related detailed pathways into larger domains of biological function. This hierarchical organization largely follows that of the Gene Ontology (GO) biological process hierarchy. The results of the over-representation analysis are provided as a color-coded interactive list of events on the 'pathways overview' of the entire Reactome event hierarchy. Each event is colored according to the probability (from a hypergeometric test) of seeing a given number or more genes in this event by chance. The darker the color, the more significant of the over-representation for a given pathway.

Single-sample GSEA (ssGSEA)

ssGSEA (Barbie et al., 2009), an extension of Gene Set Enrichment Analysis (GSEA), calculates separate enrichment scores for each pairing of a sample and gene set. Each ssGSEA enrichment score represents the degree to which the genes in a particular gene set are coordinately up- or down-regulated within a sample. We used ssGSEAProjection (v7) in the GenePattern modules to calculate the enrichment scores of hallmark gene sets (Liberzon et al., 2015) (FATTY_ACID_METABOLISM and GLYCOLYSIS) for each sample in the TCGA Colon and Rectal Cancer (COADREAD) dataset, which was downloaded from UCSC Xena (http://xena.ucsc.edu/).

Supplemental References

Anders, S., Pyl, P.T., and Huber, W. (2015). HTSeq--a Python framework to work with high-throughput sequencing data. Bioinformatics *31*, 166-169.

Barbie, D.A., Tamayo, P., Boehm, J.S., Kim, S.Y., Moody, S.E., Dunn, I.F., Schinzel, A.C., Sandy, P., Meylan, E., Scholl, C., et al. (2009). Systematic RNA interference reveals that oncogenic KRAS-driven cancers require TBK1. Nature *462*, 108-112.

Buas, M.F., Gu, H., Djukovic, D., Zhu, J., Drescher, C.W., Urban, N., Raftery, D., and Li, C.I. (2016). Identification of novel candidate plasma metabolite biomarkers for distinguishing serous ovarian carcinoma and benign serous ovarian tumors. Gynecol Oncol *140*, 138-144.

Carroll, P.A., Diolaiti, D., McFerrin, L., Gu, H., Djukovic, D., Du, J., Cheng, P.F., Anderson, S., Ulrich, M., Hurley, J.B., et al. (2015). Deregulated Myc Requires MondoA/Mlx for Metabolic Reprogramming and Tumorigenesis. Cancer Cell *27*, 271-285.

Dobin, A., Davis, C.A., Schlesinger, F., Drenkow, J., Zaleski, C., Jha, S., Batut, P., Chaisson, M., and Gingeras, T.R. (2013). STAR: ultrafast universal RNA-seq aligner. Bioinformatics *29*, 15-21.

Fabregat, A., Sidiropoulos, K., Garapati, P., Gillespie, M., Hausmann, K., Haw, R., Jassal, B., Jupe, S., Korninger, F., McKay, S., et al. (2016). The Reactome pathway Knowledgebase. Nucleic Acids Res *44*, D481-487.

Gu, H., Zhang, P., Zhu, J., and Raftery, D. (2015). Globally Optimized Targeted Mass Spectrometry (GOT-MS): Reliable Metabolomics Analysis with Broad Coverage Anal. Chem. *87*, 12355-12362.

Halachev, K., Bast, H., Albrecht, F., Lengauer, T., and Bock, C. (2012). EpiExplorer: live exploration and global analysis of large epigenomic datasets. Genome Biol *13*, R96.

Heinz, S., Benner, C., Spann, N., Bertolino, E., Lin, Y.C., Laslo, P., Cheng, J.X., Murre, C., Singh, H., and Glass, C.K. (2010). Simple combinations of lineage-determining transcription factors prime cis-regulatory elements required for macrophage and B cell identities. Mol Cell *38*, 576-589.

Krueger, F., and Andrews, S.R. (2011). Bismark: a flexible aligner and methylation caller for Bisulfite-Seq applications. Bioinformatics *27*, 1571-1572.

Langmead, B., Trapnell, C., Pop, M., and Salzberg, S.L. (2009). Ultrafast and memory-efficient alignment of short DNA sequences to the human genome. Genome Biol *10*, R25.

Li, R., Grimm, S.A., Chrysovergis, K., Kosak, J., Wang, X., Du, Y., Burkholder, A., Janardhan, K., Mav, D., Shah, R., et al. (2014). Obesity, rather than diet, drives epigenomic alterations in colonic epithelium resembling cancer progression. Cell Metab *19*, 702-711.

Liberzon, A., Birger, C., Thorvaldsdottir, H., Ghandi, M., Mesirov, J.P., and Tamayo, P. (2015). The Molecular Signatures Database (MSigDB) hallmark gene set collection. Cell Syst *1*, 417-425.

Love, M.I., Huber, W., and Anders, S. (2014). Moderated estimation of fold change and dispersion for RNA-seq data with DESeq2. Genome Biol *15*, 550.

Rao, J.N.K., and Scott, A.J. (1981). The Analysis of Categorical-Data from Complex Sample-Surveys - Chi-Squared Tests for Goodness of Fit and Independence in 2-Way Tables. J Am Stat Assoc *76*, 221-230.

Rao, J.N.K., and Scott, A.J. (1984). On Chi-Squared Tests for Multiway Contingency-Tables with Cell Proportions Estimated from Survey Data. Ann Stat *12*, 46-60.

Rao, J.N.K., and Scott, A.J. (1987). On Simple Adjustments to Chi-Square Tests with Sample Survey Data. Ann Stat *15*, 385-397.

Reyes, N.L., Banks, G.B., Tsang, M., Margineantu, D., Gu, H., Djukovic, D., Chan, J., Torres, M., Liggitt, H.D., Hirenallur-S, D.K., et al. (2014). Fnip1 regulates skeletal muscle fiber type specification, fatigue resistance, and susceptibility to muscular dystrophy. Proc. Natl. Acad. Sci. U. S. A. *112*, 424-429.

Sood, R.F., Gu, H., Djukovic, D., Deng, L., Ga, M., Muffley, L.A., Raftery, D., and Hocking, A.M. (2015). Targeted metabolic profiling of wounds in diabetic and non-diabetic mice. Wound Repair Regen. *23*, 423-434.

Sperber, H., Mathieu, J., Wang, Y.L., Ferreccio, A., Hesson, J., Xu, Z.J., Fischer, K.A., Devi, A., Detraux, D., Gu, H.W., et al. (2015). The metabolome regulates the epigenetic landscape during naive-to-primed human embryonic stem cell transition. Nat. Cell Biol. *17*, 1523-1535.

Trapnell, C., Pachter, L., and Salzberg, S.L. (2009). TopHat: discovering splice junctions with RNA-Seq. Bioinformatics *25*, 1105-1111.

Wang, S., Sun, H., Ma, J., Zang, C., Wang, C., Wang, J., Tang, Q., Meyer, C.A., Zhang, Y., and Liu, X.S. (2013). Target analysis by integration of transcriptome and ChIP-seq data with BETA. Nat Protoc *8*, 2502-2515.

Zhao, H., Sun, Z., Wang, J., Huang, H., Kocher, J.P., and Wang, L. (2014). CrossMap: a versatile tool for coordinate conversion between genome assemblies. Bioinformatics *30*, 1006-1007.
