# Supplementary material for: Transcriptome and DNA Methylome Analysis in a Mouse Model of Diet-Induced Obesity Predicts Increased Risk of Colorectal Cancer
Source: Cell Rep. Author manuscript; Available in PMC 2018 Feb 1. (PMC5793878; doi:10.1016/j.celrep.2017.12.071)
Supplement: 8 [file NIHMS937389-supplement-8.pdf]

Cell Reports

Article

# Transcriptome and DNA Methylome Analysis in a Mouse Model of Diet-Induced Obesity Predicts Increased Risk of Colorectal Cancer

## Graphical Abstract

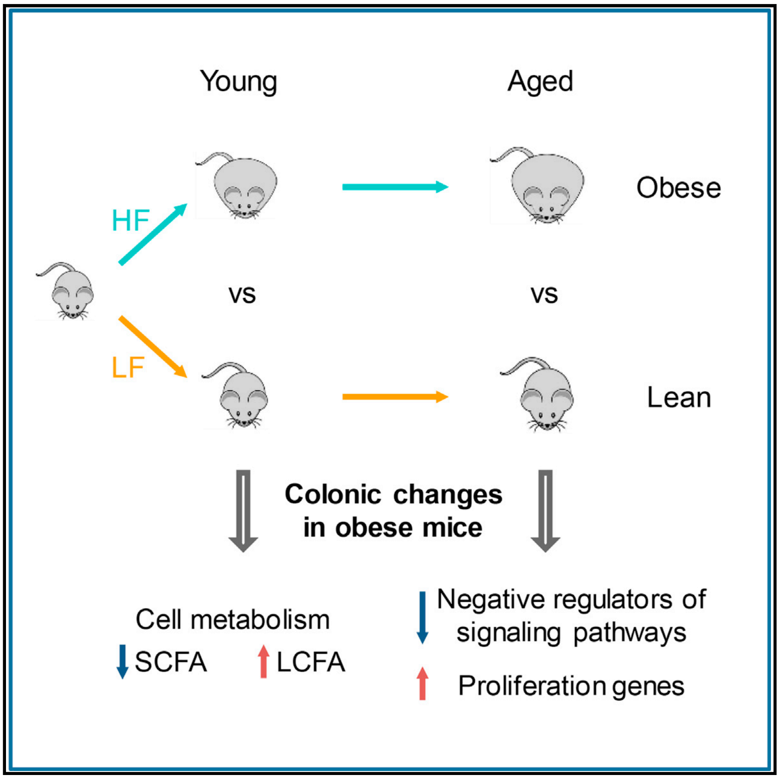


## Highlights

- Obesity reprograms mouse colonic DNA methylome, leading to gene expression changes
- Obesity triggers a metabolic switch favoring long-chain fatty acids at young age
- Obesity induces a tumor-prone gene signature in the colonic epithelium after aging
- Obesity-related changes are reversed after long-term, but not short-term, weight loss

## Authors

Ruifang Li, Sara A. Grimm, Deepak Mav, ..., B. Alex Merrick, Daniel Raftery, Paul A. Wade

## Correspondence

wadep2@niehs.nih.gov

## In Brief

Li et al. find that obesity-induced DNA methylation changes reprogram the colonic transcriptome, leading to a metabolic switch favoring long-chain fatty acid oxidation in young mice and a more tumor-prone gene signature after aging. Obesity-related changes are substantially preserved after short-term weight loss, but they are largely reversed after long-term weight loss.

## Data and Software Availability

GSE85731
GSE100276

Li et al., 2018, Cell Reports *22*, 624–637
January 16, 2018
https://doi.org/10.1016/j.celrep.2017.12.071

CellPress

OPEN ACCESS
CellPress

Cell Reports
Article

# Transcriptome and DNA Methylome Analysis in a Mouse Model of Diet-Induced Obesity Predicts Increased Risk of Colorectal Cancer

Ruifang Li,[1] Sara A. Grimm,[2] Deepak Mav,[3] Haiwei Gu,[5] Danijel Djukovic,[5] Ruchir Shah,[3] B. Alex Merrick,[4] Daniel Raftery,[5,6] and Paul A. Wade[1,7,*]
[1]Epigenetics and Stem Cell Biology Laboratory
[2]Integrative Bioinformatics
National Institute of Environmental Health Sciences, Research Triangle Park, NC 27709, USA
[3]Sciome, LLC, 2 Davis Drive, Research Triangle Park, NC 27709, USA
[4]Biomolecular Screening Branch, National Toxicology Program, National Institute of Environmental Health Sciences, Research Triangle Park, NC 27709, USA
[5]Northwest Metabolomics Research Center, Department of Anesthesiology and Pain Medicine, University of Washington, 850 Republican St., Seattle, WA 98109, USA
[6]Public Health Sciences Division, Fred Hutchinson Cancer Research Center, 1100 Fairview Ave. N., Seattle, WA 98109, USA
[7]Lead Contact
*Correspondence: wadep2@niehs.nih.gov
https://doi.org/10.1016/j.celrep.2017.12.071

## SUMMARY

**Colorectal cancer (CRC) tends to occur at older age; however, CRC incidence rates have been rising sharply among young age groups. The increasing prevalence of obesity is recognized as a major risk, yet the mechanistic underpinnings remain poorly understood. Using a diet-induced obesity mouse model, we identified obesity-associated molecular changes in the colonic epithelium of young and aged mice, and we further investigated whether the changes were reversed after weight loss. Transcriptome analysis indicated that obesity-related colonic cellular metabolic switch favoring long-chain fatty acid oxidation happened in young mice, while obesity-associated downregulation of negative feedback regulators of pro-proliferative signaling pathways occurred in older mice. Strikingly, colonic DNA methylome was pre-programmed by obesity at young age, priming for a tumor-prone gene signature after aging. Furthermore, obesity-related changes were substantially preserved after short-term weight loss, but they were largely reversed after long-term weight loss. We provided mechanistic insights into increased CRC risk in obesity.**

## INTRODUCTION

Colorectal cancer (CRC) is the third most common cancer worldwide (Ferlay et al., 2015). CRC tends to occur at a later age (>50 years old), however, CRC incidence rates have been increasing dramatically among young age groups (Siegel et al., 2017). The increasing prevalence of obesity is recognized as a major risk for CRC (Renehan et al., 2008), yet the molecular underpinnings of the link remain incompletely resolved, and even less is known about the molecular events that initiate the process. Mechanistic insights are urgently needed to pave the way for effective prevention of CRC development in obese humans. Weight loss seems to be a logical strategy to reduce CRC risk, but it is unclear whether obesity-associated changes in the colonic epithelium can be reversed after weight loss.

Epigenetic mechanisms are fundamental to phenotypic changes induced by environmental and lifestyle factors. Regarding epigenetic mechanisms underlying the link between obesity and CRC, DNA methylation is a prime candidate. First, aberrant DNA methylation is observed in virtually all CRCs (Lao and Grady, 2011); second, DNA methylation responds to obesity and weight loss leading to gene expression changes (Barres et al., 2013); and third, DNA methylation changes can persist even after the original stress/stimulus is gone (Chen et al., 2016).

To mimic human obesity, we employed a diet-induced obesity mouse model, which exhibits metabolic dysfunctions (Collins et al., 2004) and increased incidence of colon cancer (Tuominen et al., 2013) as in obese humans. To understand the mechanisms of how obesity and weight loss shape the predisposition to CRC in mice at physiologically relevant settings as in humans (Jackson et al., 2017), we examined molecular pathophysiologic changes in the colonic epithelium at two time points that are equivalent to human young (~30 years old) and older age (~50 years old). Six-week-old male C57BL/6J mice were fed a low-fat diet for 20 (or 43) weeks (LF: control mice), a high-fat diet for 20 (or 43) weeks (HF: obese mice), or a high-fat diet for 15 weeks and then switched to a low-fat diet for another 5 (or 28) weeks (HF-LF: formerly obese mice). To globally map obesity-associated changes in DNA methylation and gene expression, we performed whole-genome bisulfite sequencing (WGBS) and RNA sequencing (RNA-seq) analyses in the colonic epithelium from those mice (Table S1). We first compared young and aged obese mice with their age-matched control mice to gain insights into obesity-induced molecular pathophysiological changes in the colon at different stages of life, and then we


This is an open access article under the CC BY license (http://creativecommons.org/licenses/by/4.0/).

explored data from formerly obese mice to investigate whether those changes can be reversed after weight loss.

## RESULTS

### Dysregulation of Metabolic and Cancer-Related Genes in Colonic Epithelium of Young Obese Mice

Comparing young obese mice with age-matched control mice, 287 differentially expressed genes (DEGs) were identified using DESeq2 (Figure 1A; Table S2A). Gene expression changes were validated using real-time RT-PCR (Figure S1A). To understand the biological functions of obesity-associated DEGs, we performed Ingenuity Pathway Analysis (IPA) and gene ontology (GO) analysis. Obesity-associated DEGs were significantly enriched with genes involved in metabolic processes, such as lipid metabolism, carbohydrate metabolism, and energy production, and they were predominantly associated with cancer (249 of 287 DEGs) and gastrointestinal disease (220 of 287 DEGs) (Figure 1B; Table S2C). Since metabolic reprogramming is a hallmark of cancer (Hanahan and Weinberg, 2011), we asked whether dysregulated metabolic genes in young obese mice were related to cancer. Indeed, they largely overlapped with cancer-related DEGs (Figures 1C and S1B). We further examined whether those dysregulated metabolic genes were also differentially expressed in human CRC. The majority showed the same direction of change as in young obese mice in at least one dataset comparing human CRC with normal colon/rectum (Figure 1D). Taken together, we propose that obesity may predispose individuals to CRC via reprogramming cellular metabolism in the colonic epithelium.

### A Metabolic Switch Favoring Long-Chain Fatty Acid Oxidation in Colonic Epithelium of Young Obese Mice

Colonocytes normally use butyrate, a short-chain fatty acid derived from colonic fermentation of dietary fiber, as a major energy source (Roediger, 1982). Since fecal butyrate production was reduced in obese mice (data not shown), colonocytes in obese mice may switch to other energy sources. In concordance with the decrease in butyrate, we observed obesity-related downregulation of Acyl-CoA synthetases (*Acss1*, *Acss2*, and *Acsm3*) and Acyl-CoA dehydrogenase (*Acadsb*) that are specifically responsible for short- and medium-chain fatty acid activation and dehydrogenation during fatty acid oxidation (Table S2A). In contrast, we noticed obesity-related upregulation of *Slc27a2* and *Acaa1b* (Table S2A), which are engaged in long-chain fatty acid activation and thiolysis, respectively, implying an increase in long-chain fatty acid oxidation; consequently, the level of C12 acylcarnitine, an intermediate metabolite of long-chain fatty acid oxidation, increased in the colonic epithelium of young obese mice (Figure S1C). Consistent with the notion that long-chain fatty acid oxidation inhibits glucose utilization (Hue and Taegtmeyer, 2009), we saw concurrent downregulation of glucose metabolism genes, such as *Slc2a4* (glucose transporter), *Pfkm* (phosphofructokinase), and *Pdp1* (PDH phosphatase) (Table S2A). Collectively, our data indicate that obesity triggered a metabolic switch favoring long-chain fatty acid oxidation in the colonic epithelium. This likely reflects an adaptation to nutrient availability; nevertheless, it can boost colonic stem cell functions and promote colon tumorigenesis (Beyaz et al., 2016), as normal and cancer stem cells benefit from active fatty acid oxidation for their maintenance and function (Carracedo et al., 2013). Moreover, diminished utilization of butyrate and glucose in colonic epithelium of obese mice resembled the changes of cellular energy metabolism during colon tumorigenesis (Zhang et al., 1998).

### Altered Colonic Cellular Metabolic Profile in Young Obese Mice Resembles that of CRC

As a reflection of gene expression changes, the metabolome of colonic epithelium was also altered in young obese mice. In line with obesity-associated upregulation of *Slc7a9*, an amino acid transporter (Table S2A), targeted metabolomics analysis revealed significant increases in the levels of most amino acids in the colonic epithelium of young obese mice (Table S3A), reminiscent of a metabolic trait observed in human colon cancer (Hirayama et al., 2009). In addition, we detected a significant elevation in the level of acetylcholine (Figure S1D), which promotes colon cancer cell proliferation, migration, and invasion (Belo et al., 2011; Cheng et al., 2008). On the other hand, untargeted lipidomics analysis showed that phospholipids of specific molecular species were significantly reduced in the colonic epithelium from young obese mice (Table S3B), in keeping with obesity-associated dysregulation of phospholipid metabolism genes, such as *Lpcat1* and *Lpcat4* (Table S2A). Collectively, our data demonstrate that obesity-associated gene expression changes resulted in a cellular metabolic profile reminiscent of that of colon cancer in many aspects.

### Obesity-Related DNA Methylation Changes Occur at Distal Regulatory Regions

To explore the epigenetic basis of obesity-associated gene expression changes, we performed WGBS in the colonic epithelium (average coverage >25× and ~82.5% of CpGs with coverage ≥10× per group; Table S1A). Additionally, we carried out deep sequencing (>2,500× coverage) of bisulfite PCR amplicons (BSP-seq) containing 233 randomly selected CpG sites. Methylation levels of those CpG sites showed a strong correlation between WGBS and BSP-seq (Figure S2A), suggesting that the WGBS data coverage is sufficient. Since non-CpG methylation is rare in mouse colonic epithelium (data not shown), we studied only CpG methylation in detail. In all three experimental groups, the distribution of methylation level of single CpG sites was largely bimodal (Figure S2B), and promoters and CpG islands were lowly methylated while repetitive elements (LINE, SINE, and LTR) were highly methylated (Figure S2C), indicating that no global differences in DNA methylation were observed comparing the three groups.

Next, we used the Rao Scott Likelihood Ratio Test to identify differentially methylated regions (DMRs); 4,123 regions gained DNA methylation (hyper-DMRs) and 4,076 regions lost DNA methylation (hypo-DMRs) in obese mice compared with control mice (Figure 2A; Table S4A). Although most DMRs are outside of known gene promoters (Figure 2C), the underlying DNA sequences of obesity-associated DMRs are conserved across placental mammals (Figure 2B). We next examined whether obesity-associated DMRs overlapped with annotated

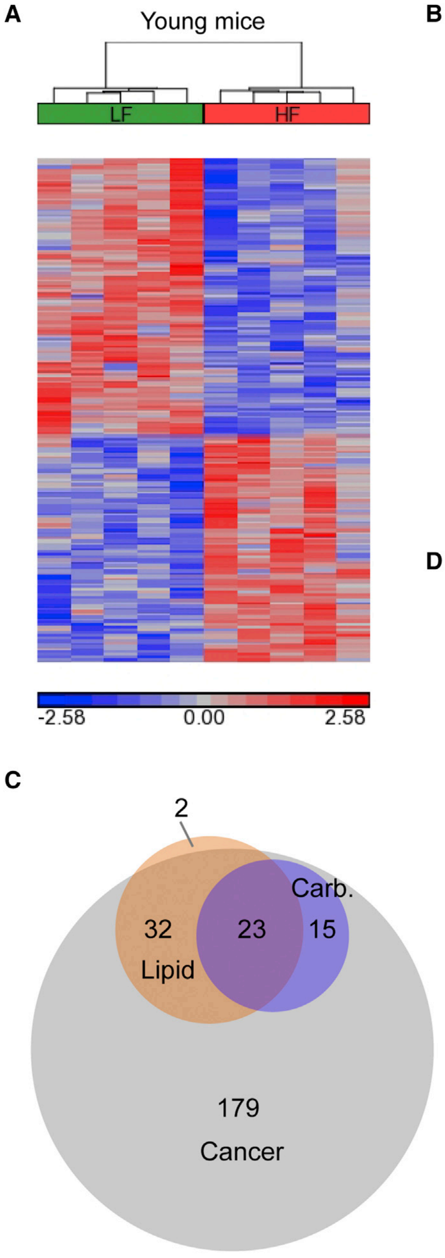


**B**

| Name | p-value | #Molecules |
|---|---|---|
| Diseases and Disorders | | |
| Cancer | 1.29E-02 - 7.57E-07 | 249 |
| Organismal Injury and Abnormalities | 1.29E-02 - 7.57E-07 | 253 |
| Gastrointestinal Disease | 1.29E-02 - 1.24E-06 | 220 |
| Metabolic Disease | 1.29E-02 - 2.32E-05 | 65 |
| Nutritional Disease | 1.61E-03 - 1.03E-04 | 10 |
| Molecular and Cellular Functions | | |
| Lipid Metabolism | 1.29E-02 - 1.61E-08 | 57 |
| Small Molecule Biochemistry | 1.29E-02 - 1.61E-08 | 72 |
| Carbohydrate Metabolism | 1.29E-02 - 1.47E-07 | 38 |
| Molecular Transport | 1.29E-02 - 6.11E-07 | 64 |
| Energy Production | 1.29E-02 - 3.39E-06 | 14 |

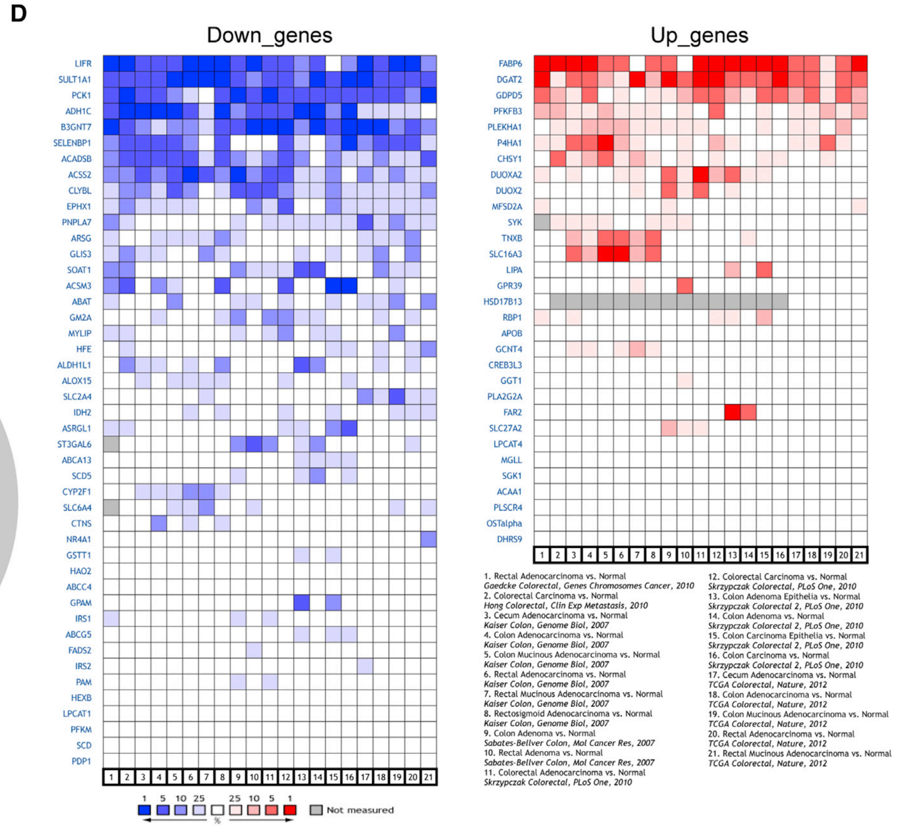


**Figure 1. Obesity-Related DEGs in Young Mice Are Significantly Enriched with Metabolic and Cancer-Related Genes**

(A) The heatmap depicts the standardized expression levels of DEGs in young obese mice (HF) relative to control mice (LF). Red indicates overexpression and blue indicates underexpression.

(B) IPA of obesity-related DEGs in young mice. The top five scoring hits in each functional category are shown, together with p values and the numbers of dysregulated genes in the enriched terms.

(C) Overlap of dysregulated lipid and carbohydrate metabolism genes with dysregulated cancer-related genes in young obese mice.

(D) The expression changes (blue, underexpression; red, overexpression) of lipid, carbohydrate, and amino acid metabolism genes that were dysregulated in young obese mice, in human CRC compared with normal colon/rectum. Left and right panels exhibit obesity-related down- and upregulated genes, respectively. See also Figure S1 and Tables S2 and S3.

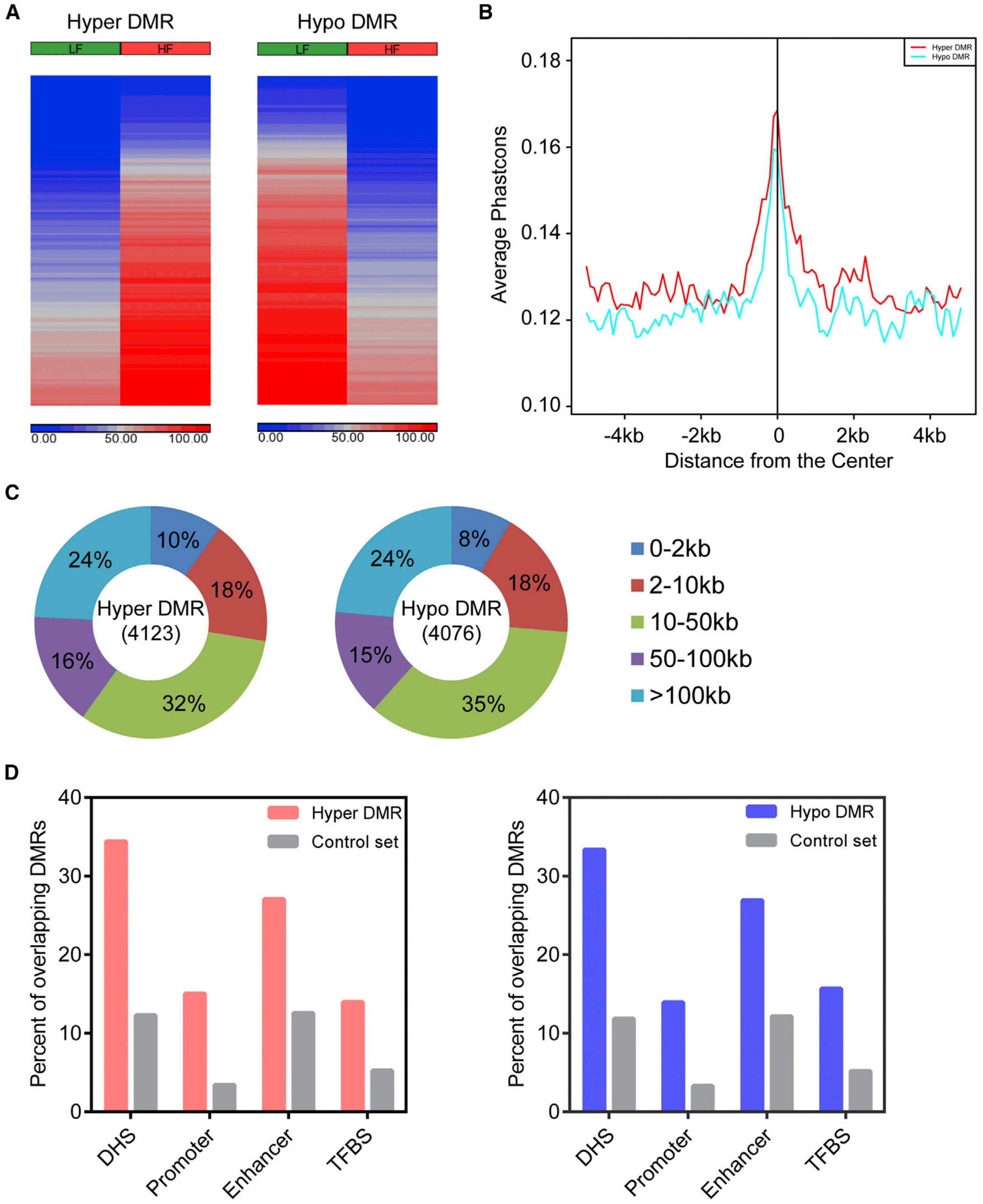


**Figure 2. Obesity-Related DNA Methylation Changes Occur at Distal Regulatory Regions**

(A) The heatmaps depict DNA methylation levels of DMRs in young obese (HF) and control (LF) mice. Blue indicates unmethylated and red indicates fully methylated.

(B) The underlying DNA sequences of DMRs are conserved across placental mammals. The average placental PhastCons score was plotted for a 10-kb window centered at the midpoint of DMRs.

(C) Distance of DMRs to the nearest RefSeq gene transcription start site (TSS).

(D) The percentages of DMRs or matched control regions overlapping with *cis*-regulatory regions defined using mouse ENCODE data.

See also Figure S2 and Tables S1 and S4.

genomic features and experimentally defined functional genomic elements. Higher percentages of DMRs than control regions coincided with regions marked by H3K4me1, H3K4me3, and DNase-hypersensitive sites (DHSs), whereas DMRs rarely occurred at CpG islands (Figure S2D). Furthermore, obesity-associated DMRs significantly overlapped with *cis*-regulatory regions (DHSs, promoters, enhancers, and TFBS) defined using mouse Encyclopedia of DNA Elements (ENCODE) data from over 100 mouse cell types and tissues (Figure 2D) (Yue et al., 2014). Notably, a larger number of DMRs overlapped with enhancers and H3K4me1-marked regions than with promoters and H3K4me3-marked regions (Figures 2D and S2D). Taken together, obesity-associated DNA methylation changes in the colonic epithelium mainly occurred at distal regulatory regions.

## Obesity-Related DMRs Are Significantly Associated with DEGs

Given that DMRs largely overlapped with regulatory regions in the genome, we hypothesized that obesity-related DNA methylation changes may affect gene expression. To test this hypothesis, we employed binding and expression target analysis (BETA) (Wang et al., 2013). BETA integrates differentially bound/modified regions (user-defined regions) with differential gene expression data to determine whether user-defined regions have overall activating and/or repressing functions and to detect their target genes. We ran BETA with hyper-DMRs and hypo-DMRs separately. Both types of DMRs were significantly enriched near both up- and downregulated genes, indicating that DNA methylation at distal regulatory regions may have both activating and repressing functions (Spruijt and Vermeulen, 2014); however, hyper-DMRs and hypo-DMRs were more significantly associated with downregulated and upregulated genes, respectively (Figure 3A). With a rank product (RP, equivalent to p value) cutoff of less than 0.001, BETA found 321 DMR target genes. A subset of them (108) were significantly differentially expressed (Figure 3B), while the rest exhibited a clear trend toward differential expression, although they did not reach statistical significance (Figures S3A and S3B), suggesting that obesity-associated DNA methylation changes are complementary to, but not exclusively overlapping with, changes in gene expression. Other than directly regulating transcription, altered DNA methylation may reflect historical changes or prime for future changes in gene expression.

To infer the biological impacts of obesity-associated DNA methylation changes, we performed IPA of DMR target genes. They were predominantly involved in cancer (267 of 321) and gastrointestinal disease (229 of 321), and they were significantly enriched with lipid and carbohydrate metabolism genes (Figure 3C). Correlations between DNA methylation and gene expression were shown at several metabolic genes (Figures 3D and S3C). Overall, obesity-associated DNA methylation changes were significantly associated with gene expression changes in the colonic epithelium, especially at metabolic genes related to cancer, in keeping with the notion that aberrant DNA methylation at distal regulatory regions regulates gene expression and modulates the predisposition to cancer (Aran and Hellman, 2013; Aran et al., 2013).

## Obesity-Related DNA Methylation Changes Also Prime for Future Changes in Gene Expression

To test whether obesity-related DNA methylation changes prime for future gene expression changes at a later stage of life, we ran BETA with obesity-related DMRs from young mice and differential gene expression data from aged mice. We reasoned that, if that is true, those DMRs should also show significant association with obesity-related DEGs from aged mice. In fact, those DMRs exhibited even more significant associations with DEGs from aged mice, especially with downregulated genes, than with DEGs from young mice (Figure 4).

Next, we explored the mechanistic explanation for that. Since a substantial number of DMRs overlapped with regulatory regions (Figures 2D and S2D) and DNA methylation changes may affect transcription factor binding at those sites, we thus searched for transcription factor motifs enriched at obesity-associated DMRs using Hypergeometric Optimization of Motif Enrichment (HOMER) motif analysis (Heinz et al., 2010) (Table S4C). We then determined upstream regulators of obesity-related DEGs from aged mice using Ingenuity upstream regulator analysis (Table S5B). We found that obesity-associated DMRs from young mice are enriched with motifs of transcription factors that are upstream regulators of obesity-related DEGs from aged mice (Tables S4C and S5B). For example, DMRs are enriched with binding sites of ELK1, ELK4, and E2F transcription factors (Figure S4A). E2F family members play important roles in cell cycle progression, while ELK1 and ELK4 are cofactors of serum response factor (SRF) (Buchwalter et al., 2004), which regulates many immediate early genes (Schratt et al., 2001). Concordantly, we observed differential expression of their target genes, such as immediate early genes and genes related to cell cycle, in aged obese mice compared with age-matched control mice (Figures S4B and S4C). Altogether, our data suggest that obesity may imprint regulatory regions at young age, priming for future changes in gene expression at a later stage of life.

## A Tumor-Prone Gene Signature in the Colonic Epithelium of Aged Obese Mice

Next, we explored the biological functions of dysregulated genes in obese mice at a later stage of life, which is equivalent to the age of a human when CRC incidence rate dramatically increases. Obesity-associated downregulated genes in aged mice are mainly related to signal transduction and transcriptional regulation (Figure 5B; Tables S5A, S5D, S5E, and S5G). We observed decreased expression of negative regulators of several signaling pathways (Table S5A), including the EGFR/RTK-RAS-ERK/MAPK cascade (*Errfi1*, *Spry1*, *Rasa4*, *Dusp1*, and *Dusp5*) (Ferby et al., 2006; Hanafusa et al., 2002; Lockyer et al., 2001; Mandl et al., 2005; Slack et al., 2001), nuclear factor κB (NF-κB) signaling (*Nfkbia*, *Nfkbiz*, and *Tnfaip3*) (Renner and Schmitz, 2009; Shembade et al., 2010), transforming growth factor β (TGF-β) signaling (*Smad6*, *Smad7*, and *Spsb1*) (Imamura et al., 1997; Liu et al., 2015; Nakao et al., 1997), JAK/STAT signaling (*Cish*) (Yoshimura, 1998), and mTORC1 (*Mark4*) (Li and Guan, 2013). These signaling pathways control cell proliferation, differentiation, migration, and survival; hence their dysregulations play important roles in tumor development and progression.

A

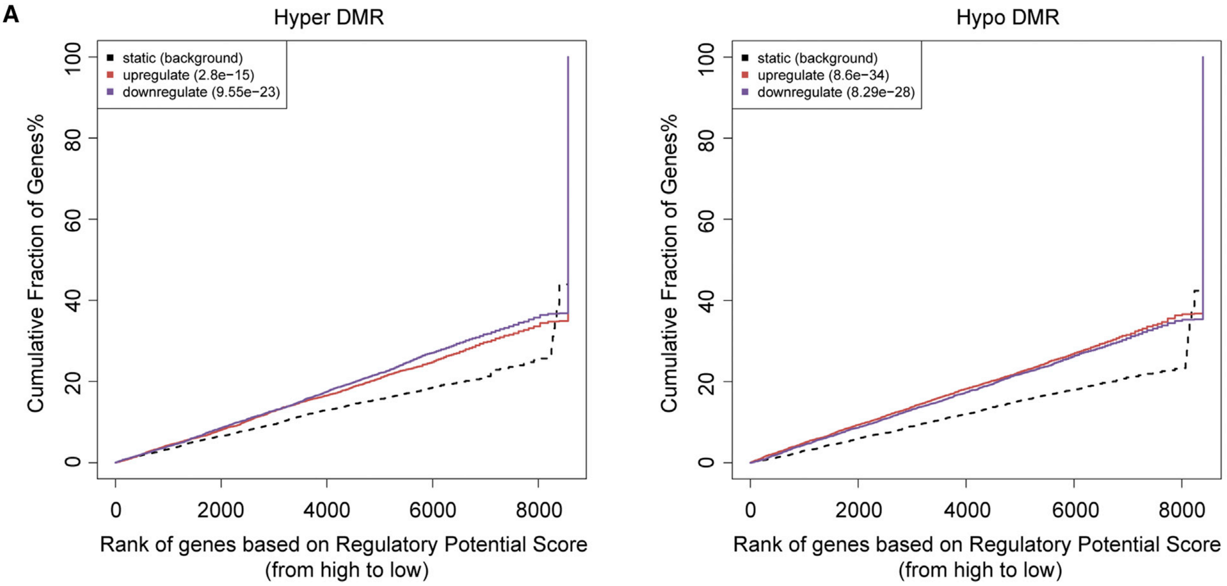


B

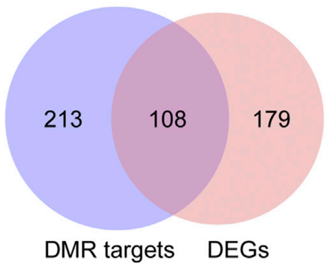


C

| Name | p-value | #Molecules |
|---|---|---|
| Diseases and Disorders | | |
| Cancer | 1.43E-02 - 5.74E-06 | 267 |
| Organismal Injury and Abnormalities | 1.43E-02 - 5.74E-06 | 273 |
| Reproductive System Disease | 1.43E-02 - 8.01E-05 | 113 |
| Gastrointestinal Disease | 1.43E-02 - 1.51E-04 | 229 |
| Connective Tissue Disorders | 1.43E-02 - 2.28E-04 | 13 |
| Molecular and Cellular Functions | | |
| Cell Death and Survival | 1.43E-02 - 1.09E-07 | 109 |
| Lipid Metabolism | 1.43E-02 - 2.35E-06 | 47 |
| Molecular Transport | 1.43E-02 - 2.35E-06 | 53 |
| Small Molecule Biochemistry | 1.43E-02 - 2.35E-06 | 58 |
| Carbohydrate Metabolism | 1.43E-02 - 6.97E-06 | 33 |

D

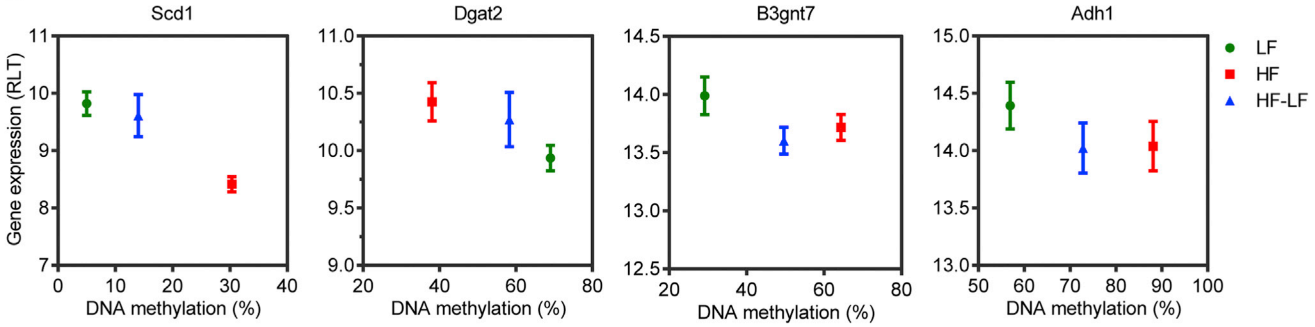


**Figure 3. Obesity-Related DMRs Are Significantly Associated with DEGs**

(A) BETA with hyper-DMRs (left panel) or hypo-DMRs (right panel) and differential gene expression data (obese versus control) from young mice. The dotted line represents background genes not differentially expressed, whereas the red and the blue lines represent up- and downregulated genes in obese mice, respectively. The y axis indicates the proportion of genes in a category that are ranked at or better than the x axis value, which represents the rank on the basis of the regulatory potential score from high to low. The p values listed on the top left represent the significance of the UP or DOWN group relative to the NON group, as determined by Kolmogorov-Smirnov test.

(B) Overlap of DMR target genes with obesity-related DEGs from young mice.

(legend continued on next page)

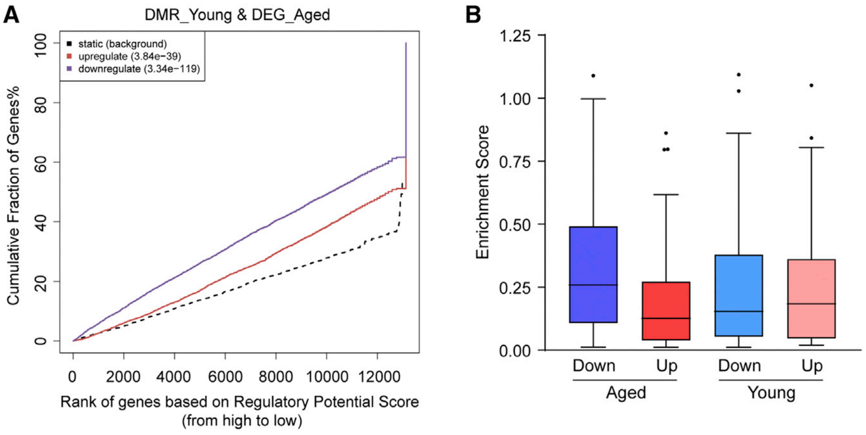


**Figure 4. Obesity-Related DNA Methylation Changes at Young Age Prime for Future Gene Expression Changes after Aging**

(A) BETA with obesity-related DMRs from young mice and differential gene expression data from aged mice.

(B) The enrichment scores of DMRs within 100 kb of obesity-related up- or downregulated genes from young and aged mice. In the box and whisker plot, the box indicates the 25th to 75th percentile, whiskers indicate 1.5 times the inter-quartile distance.

See also Figure S4 and Table S4.

Normally, intracellular signal transduction is kept under tight control via negative feedback loops to ensure physiologically appropriate signaling outcomes. With downregulation of negative feedback regulators (Table S5A), the braking system of those signaling pathways were dampened in aged obese mice, potentially leading to unrestrained signaling. In parallel with this, the Jun N-terminal kinase (JNK) and p38 MAPK pathways, which are also called stress-activated protein kinase (SAPK) pathways, were likely attenuated in aged obese mice due to reduced expression of key mediators of SAPK pathways (*Map4k2*, *Map4k5*, and *Mapk12*) (Table S5A) (Chin et al., 1999; Pombo et al., 1995; Shi et al., 1999). SAPK pathways are activated by various types of environmental and pathophysiological stresses with anti-proliferative and pro-apoptotic effects. Given that the colonic epithelium is under more cell-intrinsic and -extrinsic stresses in obese mice than in control mice (Gulhane et al., 2016), the attenuation of SAPK pathways in aged obese mice might be a result of cell-autonomous adaptation to sustained stresses. Nevertheless, the insensitivity to anti-growth signals likely provides an advantage for cells to survive and proliferate under stressful conditions. In addition, downregulated genes in aged obese mice were significantly enriched with transcription factors (Table S5E), some of which are encoded by primary response genes. A total of 35 primary response genes were downregulated in aged obese mice (Figure 5C), including both immediate early genes and delayed early genes (Amit et al., 2007; Tullai et al., 2007); the majority were also downregulated in human CRC compared with normal colorectal tissue or colorectal adenoma (Figures S5A and S5B), presumably reflecting independence of mitogenic signals—a hallmark of cancer. Since delayed early genes are potential tumor suppressors (Amit et al., 2007), we investigated whether downregulated genes are enriched for tumor suppressor genes (Zhao et al., 2016). A substantial number of tumor suppressor genes were repressed in aged obese mice (Figure 5D). Collectively, downregulation of those genes in aged obese mice likely weakens proliferation barriers, thus favoring cell-autonomous growth.

Consistently, obesity-associated upregulated genes in aged mice are significantly associated with cell cycle, DNA replication, DNA repair, and chromatin organization (Figure 5A; Tables S5A, S5C, and S5F), including genes with functions in nucleosome assembly, centromeres, kinetochore organization, spindle assembly checkpoint, and chromosome condensation and segregation (Table S5C). In addition, we observed increased expression of genes involved in amino acid biosynthetic process, aminoacyl-tRNA biosynthesis, and deoxyribonucleotide biosynthetic process in aged obese mice (Tables S5A and S5C), presumably fulfilling the enhanced requirements for protein and DNA synthesis during cell proliferation. Accordingly, subunits of complex I of the respiratory chain (*Ndufa3*, *Ndufa4*, *Ndufa8*, and *Ndufa12*) and a subunit of mitochondrial ATP synthase (*Atp5g1*) were also upregulated (Table S5A), likely generating more energy to fuel the enhanced biosynthetic reactions and cell division. Collectively, upregulation of those genes may reflect increased cell proliferation of the colonic epithelium in aged obese mice compared with age-matched controls (Beyaz et al., 2016), even though no colon tumors were present in those mice.

## Reversibility of Obesity-Related Colonic Changes after Short- and Long-Term Weight Loss

To investigate whether obesity-related changes in the colonic epithelium can be reversed after weight loss, we put mice on a high-fat diet for 15 weeks to induce obesity, and then we switched them to a low-fat diet for 5 weeks to trigger weight loss. Body weights of these mice returned to normal by the end of the study (Figure 6A). We then analyzed DNA methylation and gene expression data from these formerly obese mice. We clustered obesity-related DMRs into 6 groups (C1–C6) based on their methylation levels in formerly obese mice; C1 retained methylation changes, C2 lost methylation changes, while C3 and C4 exhibited intermediate methylation levels after weight loss (Figure S6A). Among obesity-related DMRs, 1,215

(C) IPA of DMR target genes. The top five scoring hits in each functional category are shown, together with p values and the number of DMR target genes in the enriched terms.

(D) Correlation between DNA methylation and gene expression at several metabolic genes.

Error bars indicate SD. See also Figure S3.

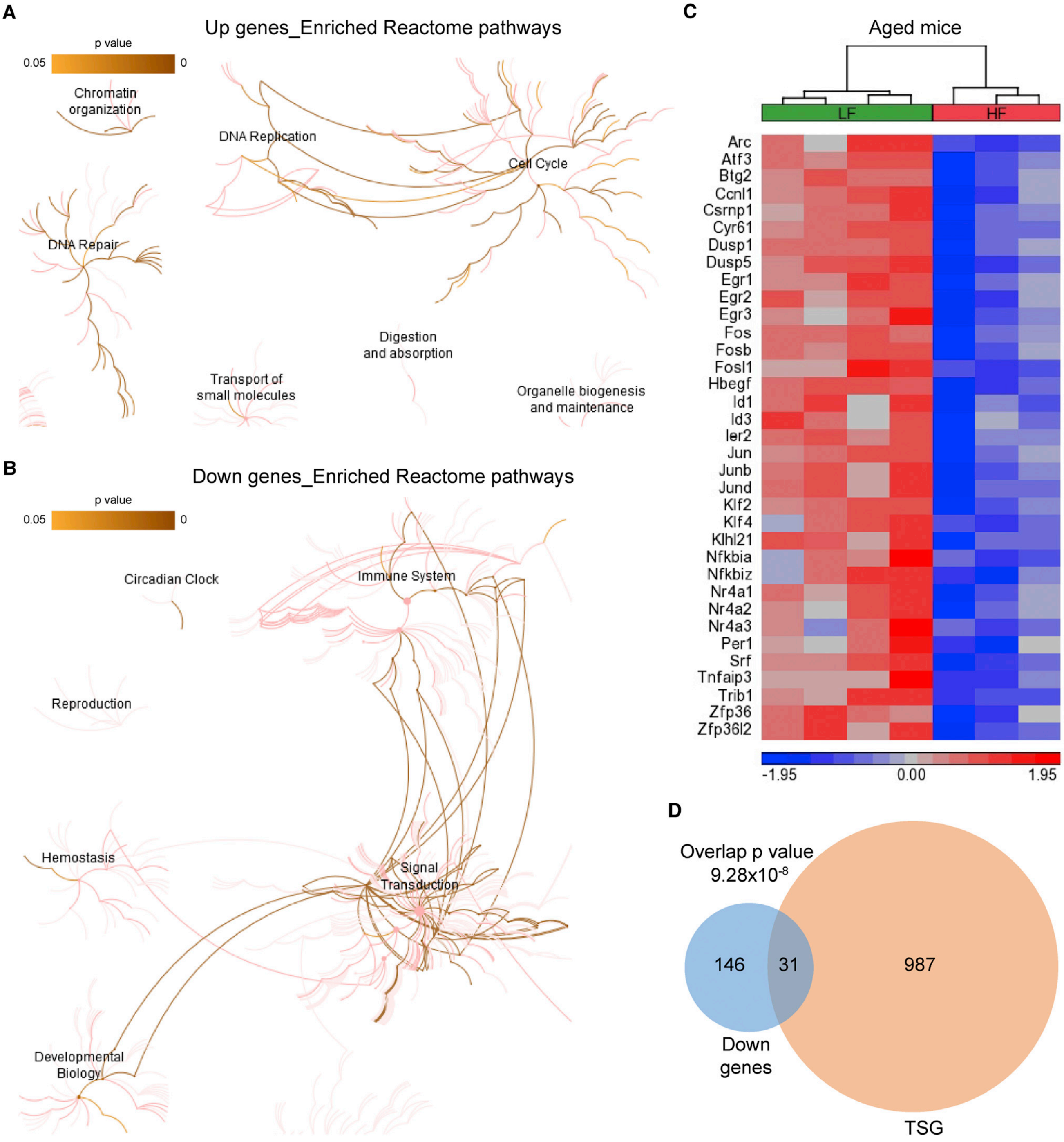


**Figure 5. A Tumor-Prone Gene Signature in the Colonic Epithelium of Aged Obese Mice**

(A and B) The results of Reactome pathway enrichment analysis are shown for up- (A) and downregulated genes (B) in aged obese mice. The Reactome hierarchical pathway structure is shown with color corresponding to the significance of p values. The darker color is more significant.

(C) The heatmap depicts the standardized expression levels of the 35 primary response genes in aged obese and control mice.

(D) Overlap of tumor suppressor genes (TSGs) with downregulated genes in aged obese mice. Overlap p value was calculated using hypergeometric test.

See also Figure S5 and Table S5.

hyper-DMRs and 1,040 hypo-DMRs retained methylation changes after weight loss (Figure 6B; Table S4B). Retained DMRs were associated with genes primarily involved in cancer and cellular functions pertinent to cancer, such as cell death and survival, cell-to-cell signaling and interaction, cellular development, and cellular growth and proliferation (Figure 6C). Two

examples (*Hoxb13* and *Spry1*) with persistent changes after weight loss were shown (Figure S3C). Altogether, our data revealed an epigenetic memory of the previous obese state in the colonic epithelium after short-term weight loss.

We used multiple approaches to assess the similarity of gene expression changes in formerly obese mice and obese mice. First, using overlap analysis, we found that 107 obesity-associated DEGs (51 upregulated and 56 downregulated) were also significantly dysregulated in formerly obese mice in the same direction as in obese mice (Figure S6B). These commonly dysregulated genes were predominantly associated with cancer (102 of 107) and gastrointestinal disease (96 of 107), and they were significantly enriched for lipid and carbohydrate metabolism genes (Figure S6C; Table S2E). Second, we performed hierarchical clustering of samples on obesity-associated dysregulated metabolic genes. Formerly obese mice were clustered together with obese mice instead of control mice (Figure 6D), indicating that the expression levels of those genes in formerly obese mice were more close to those in obese mice than in control mice. Lastly, comparing enriched diseases and biological functions in formerly obese mice with those in obese mice, we noted that the two groups were significantly enriched with the same terms, mainly related to intestinal tumor and lipid and carbohydrate metabolism (Figures 6E and S6D; Tables S2C and S2D). Taken together, persistent changes in gene expression were observed after short-term weight loss, primarily at genes closely related to colon cancer.

Considering that those formerly obese mice just returned to normal weight after 5 weeks of diet-switching, to investigate whether obesity-related dysregulation of gene expression can be completely reversed after long-term weight loss, we performed RNA-seq in the colonic epithelium of mice with diet-switching for 28 weeks. Body weights of these mice returned to normal after 5 weeks of diet-switching, and they stayed at the same level as control mice since then (Figure S6E). Using hierarchical cluster analysis based on expression levels of obesity-related DEGs, we found that, after long-term weight loss, formerly obese mice were clustered together with control mice instead of obese mice (Figure S6F), indicating that obesity-related gene expression changes were also largely reversed.

Collectively, obesity-related changes in DNA methylation and gene expression were substantially preserved after short-term weight loss, but gene expression changes mostly went back to normal after long-term weight loss.

### Fatty Acid Metabolism Is Correlated with Clinical Outcomes of CRC Patients

The genes altered in obese mice at both life stages are involved in fatty acid metabolism (Figures S5C and S5D). The best-known metabolic perturbation in cancer cells is the Warburg effect, increased glycolysis even in the presence of oxygen, while alterations in fatty acid metabolism in cancer cells are less well studied. Therefore, we examined the changes in fatty acid metabolism in CRC. Most genes involved in short- and long-chain fatty acid oxidation exhibited significantly lower expression in CRCs than in adjacent normal tissues (Figure S7A). The heterogeneity of fatty acid metabolism among CRCs (Figure S7A) prompted us to investigate whether metabolic features of CRCs are related to clinical outcomes of CRC patients.

We characterized metabolic features of CRCs based on their changes in fatty acid metabolism and glycolysis. Single-sample gene set enrichment analysis (ssGSEA) (Barbie et al., 2009) was used to assess gene set enrichment score, which represents activation level of the corresponding biological process, for 380 primary CRCs along with 50 adjacent normal tissues from The Cancer Genome Atlas (TCGA) database. CRC samples were then stratified into four groups based on the degrees of downregulation of fatty acid metabolism and upregulation of glycolysis (Figures 7A and S7D). Decreased fatty acid metabolism was significantly associated with poor overall survival of CRC patients; however, increased glycolysis had no significant impact on patient overall survival (Figures 7C, 7D, and S7B). Moreover, CRC samples with decreased fatty acid metabolism showed more lymphatic invasion (Figures 7B and S7C). Although it is the first and most common metabolic abnormality observed in cancer, the Warburg effect might just reflect a metabolic adaptation associated with rapid cell proliferation (Figure S7E) (Shyh-Chang et al., 2013). In contrast, since tumor cells generally retain substantially the metabolic feature of corresponding normal tissue (Hu et al., 2013), the degree of fatty acid metabolism decline in CRC likely represents the extent of deviation from normal colon, thus showing better clinical relevance.

## DISCUSSION

Cellular metabolism was once thought to be a mere consequence of cellular state; it is now recognized as a key player in cell fate determination (Shyh-Chang et al., 2013). Colonic gene expression changes in young obese mice suggest a metabolic switch favoring long-chain fatty acid oxidation in the colonic epithelium (Table S2A; Figure S1C). Active fatty acid oxidation is important for the maintenance and function of normal and cancer stem cells (Carracedo et al., 2013). As butyrate, the major energy source of differentiated colonocytes, inhibits intestinal stem/progenitor cell proliferation (Kaiko et al., 2016), we reasoned that long-chain fatty acid oxidation may be essential to intestinal stem cells. Indeed, Beyaz et al. (2016) recently reported that long-chain fatty acid treatment led to the increased number and enhanced function of intestinal stem cells through the induction of PPAR-δ target genes that are involved in long-chain fatty acid oxidation. Since enforced PPAR-δ activation also augmented stemness of intestinal progenitor cells and boosted their capacity to initiate tumors (Beyaz et al., 2016), we postulate that long-chain fatty acid oxidation may play important roles in intestinal cell fate determination and intestinal tumor initiation. Therefore, our data suggest that obesity-associated colonic cellular metabolic reprogramming at young age can be an initiating event, which promotes colon tumor development by increasing the number of cells with tumorigenic potential. In addition, fatty acid metabolism was associated with clinical outcomes of CRC patients (Figures 7B and 7C). Future research is needed to elucidate the relationship between obesity and cellular metabolism in CRC.

The epigenetic machinery is highly responsive to metabolic cues, because it relies on intermediate metabolites as substrates

OPEN
ACCESS
CellPress

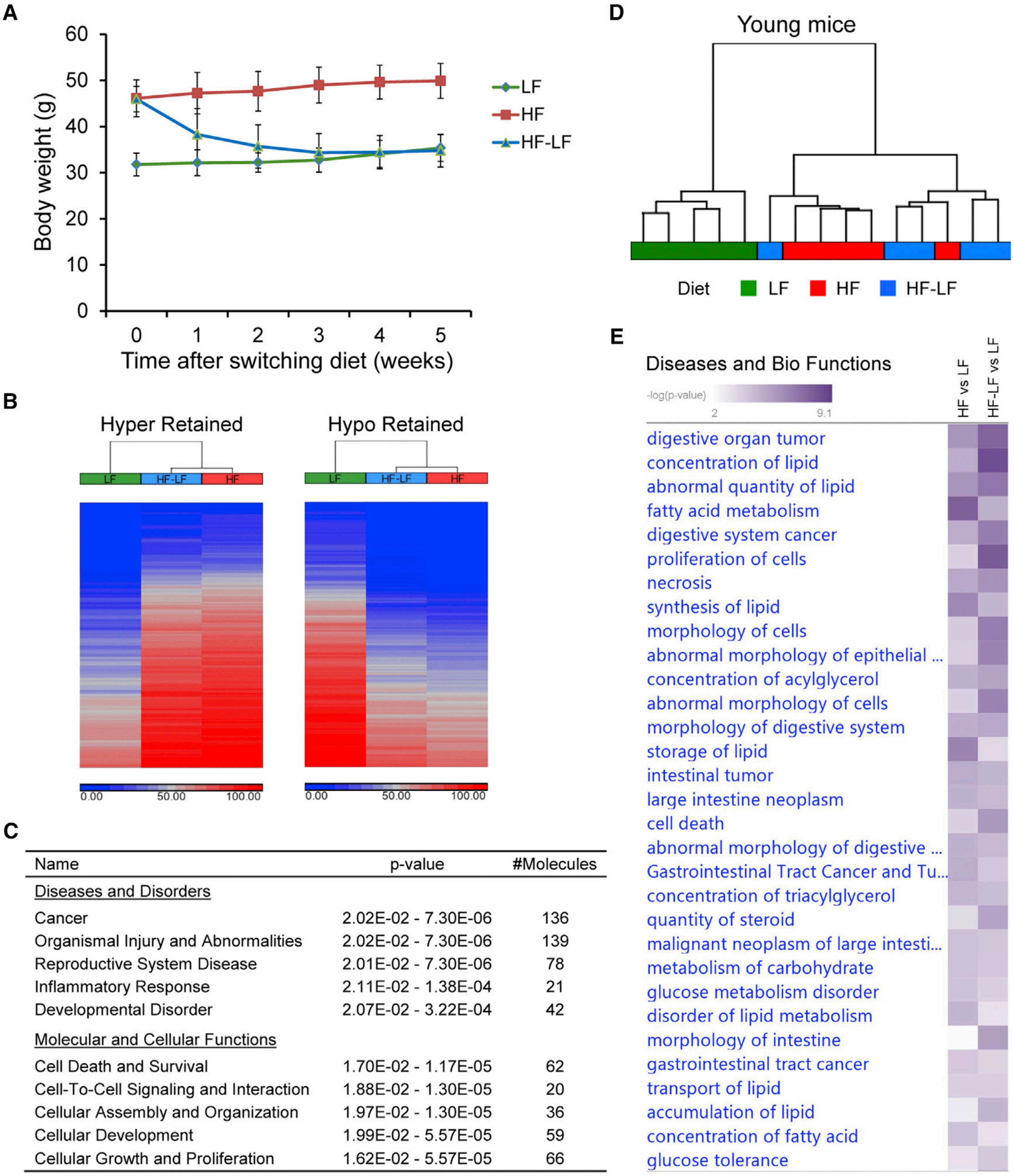


**Figure 6. Persistent Changes in DNA Methylation and Gene Expression after Short-Term Weight Loss**

(A) Weekly body weight of control mice (LF), obese mice (HF), and formerly obese mice (HF-LF) after diet-switching. Data are represented as mean ± SEM (n = 5).

(B) The heatmaps depict DNA methylation levels of retained DMRs in each group. Blue and red indicate unmethylated and fully methylated, respectively.

(C) IPA of genes associated with retained DMRs. The top five scoring hits in each functional category are shown, together with p values and the number of retained DMR-associated genes in the enriched terms.

(D) Hierarchical clustering of samples at obesity-associated dysregulated metabolic genes. Ward's method was used with Euclidean distance calculated using standardized gene expression levels.

(E) IPA comparison analysis showing the similarity between formerly obese mice and obese mice regarding the enriched diseases and biological functions.

See also Figure S6 and Table S2.

or cofactors (Sharma and Rando, 2017). Obesity-related dysregulation of colonic cellular metabolism was accompanied by comprehensive DNA methylation changes, which were enriched at regulatory regions (Figure 2). Strikingly, these DNA methylation changes were significantly associated with future gene expression changes (Figure 4). Epigenetic priming at enhancers

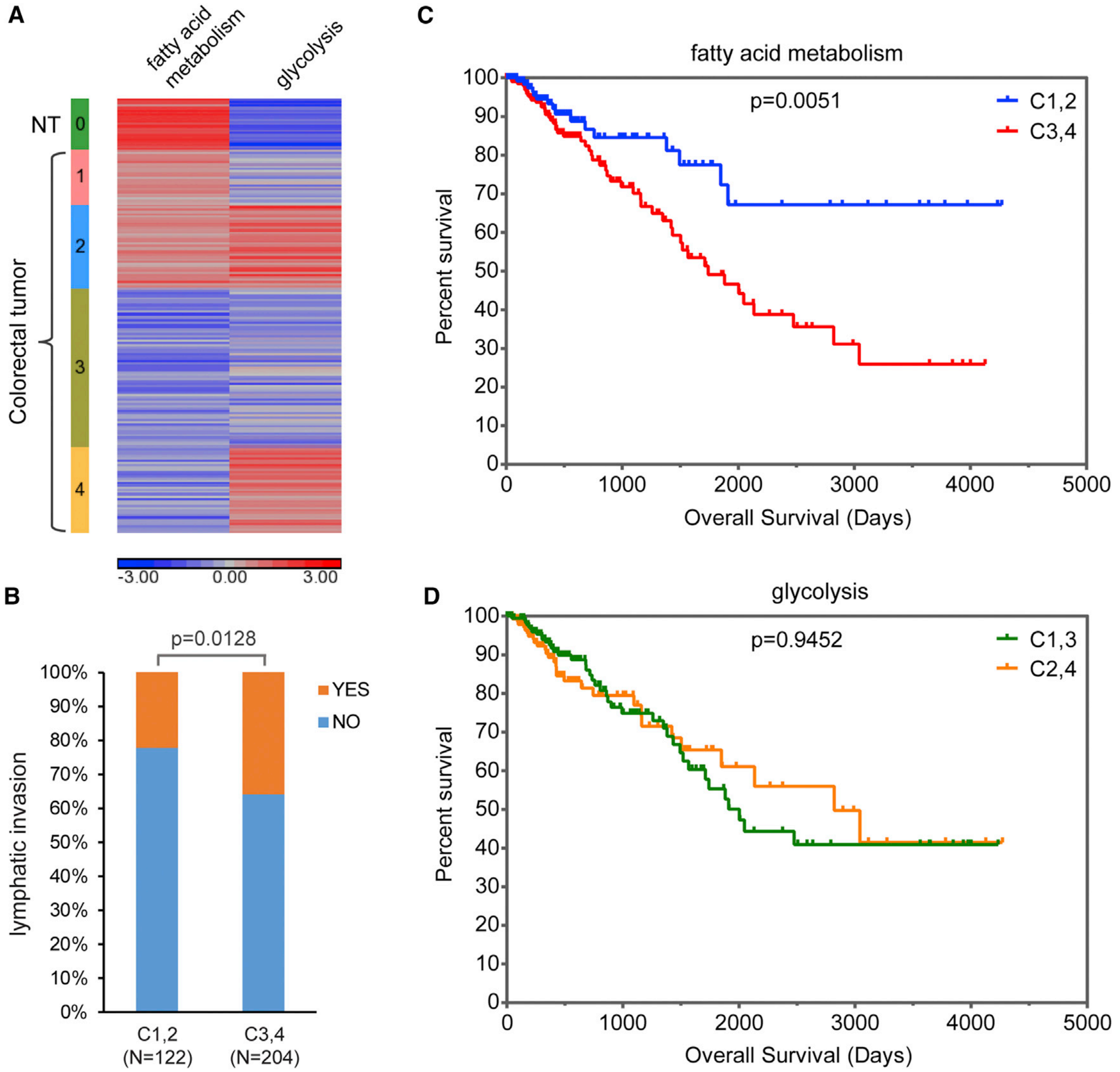


**Figure 7. Fatty Acid Metabolism Is Associated with the Clinical Outcomes of CRC Patients**

(A) Stratification of CRC samples based on ssGSEA enrichment scores of two hallmark gene sets, FATTY_ACID_METABOLISM and GLYCOLYSIS.

(B) The percentages of CRC patients with lymphatic invasion in the two groups defined by the fatty acid metabolism signature of their tumor samples.

(C and D) Kaplan-Meier plots depict the overall survival of CRC patients stratified by either fatty acid metabolism (C) or glycolysis (D) in their tumor samples. See also Figure S7.

was observed during developmental processes (Wang et al., 2015). Presumably, it also plays roles in obesity-related pathologic processes. Hence, our data imply that, in addition to immediate effects, obesity may have latent deleterious effects, which are pre-programmed in the DNA methylome and would manifest over time, if the individual is continuously obese. Persistent obesity led to the attenuation of proliferation barriers after aging.

A striking feature of colonic gene expression changes, in aged obese mice compared with age-matched controls, is the downregulation of integral components of SAPK pathways and the downregulation of negative feedback regulators of pro-survival and pro-proliferative signaling pathways, including the EGFR/RTK-RAS-ERK/MAPK cascade, NF-κB signaling, TGF-β signaling, JAK/STAT signaling, and mTORC1 (Table S5A). These comprehensive dysregulations of signaling networks could lead to extensive changes in vital cellular processes, especially in cell proliferation and survival. In supporting this hypothesis, intestinal crypts from aged obese mice (~1 year old) exhibited niche-independent growth and better survival and regeneration after irradiation (Beyaz et al., 2016). The attenuation of feedback inhibition at multiple tiers of pro-proliferative cascades and the diminishment of anti-proliferative signaling break the balance for homeostatic regulation, rendering the cells hypersensitive to otherwise limiting amounts of growth factors and probably even capable of cell-autonomous proliferation independent of exogenous mitogenic signals, and meanwhile resistant to stress signals. Hence, we propose that cell-intrinsic rewiring of signal transduction

networks in response to long-term obesity may facilitate malignant transformation of colonic epithelial cells.

Remarkably, obesity-related changes in DNA methylation and gene expression were substantially preserved after short-term weight loss, but gene expression changes were largely reversed after long-term weight loss (Figures 6 and S6), suggesting that obesity-related pathophysiological process in the colonic epithelium can be prevented with long-term weight loss. Thus, it is important for obese individuals to lose weight early.

In summary, we profiled colonic DNA methylome, transcriptome, and metabolome to identify obesity-related molecular pathophysiological changes in the colon. Those changes were not due to alterations of cell composition in the colonic epithelium, since no significant expression changes of cell-type-specific marker genes were observed in obese mice (Table S6). We provided novel mechanistic insights into how obesity increases CRC risk at different stages of life. At young age, obesity was associated with a colonic cellular metabolic switch favoring long-chain fatty acids, which were previously demonstrated to boost the numbers of intestinal stem/stem-like cells (Beyaz et al., 2016). Consequently, the chance is increased for an obese individual to gain oncogenic mutations in colonic stem cells. After aging, obesity was associated with decreased expression of tumor suppressor genes and negative feedback regulators of pro-survival and pro-proliferation signaling pathways. These changes prime for unrestrained signaling to accelerate the initiation and progression of colon tumorigenesis once oncogenic events occur. It should be noted that, without additional oncogenic driving force, obesity-related colonic molecular changes unlikely lead to colon cancer. Since the mice in our study were raised in a specific pathogen-free environment, no colon tumors were observed at the end of the study. However, obesity-related colonic molecular changes increase the odds of colon cancer development and progression, if they co-occur with oncogenic insults. Therefore, the lifetime risk of CRC increases in the obese.

Although colon tumors occur less frequently than small intestine tumors in mice unlike humans (Newmark et al., 2009), we used mouse colon as a proxy of human colon due to their similarities in tissue structure, cell composition, and physiological functions. One limitation of our study is that only male mice were used. Since female obese mice exhibited similar patterns of changes as male obese mice in colonic enhancer landscape and intestinal stem/progenitor cell functions (Beyaz et al., 2016; Li et al., 2014), we predict that it is also the case for DNA methylation and gene expression changes.

## EXPERIMENTAL PROCEDURES

### Mice

Six-week-old male C57BL/6J mice were fed either a low-fat diet (10% fat diet, D12450B; Research Diets) or a high-fat diet (60% fat diet, D12492; Research Diets). The source of fat in the diets is lard. Specifically, five mice were put on each dietary regimen as follows: (1) low-fat diet for 20 (or 43) weeks; (2) high-fat diet for 20 (or 43) weeks; and (3) high-fat diet for an initial 15 weeks and then switched to a low-fat diet for another 5 (or 28) weeks. The mice were housed in a specific pathogen-free facility. Body weight was measured weekly after diet-switching. At the end of the study, the mice were humanely euthanized and the colons were collected for further experiments. All animal experiments were approved by the NIEHS Animal Care and Use Committee, and they were performed according to NIH guidelines for care and use of laboratory animals.

### Isolation of the Colonic Epithelium and DNA/RNA Preparation

The colonic epithelium was isolated as previously described (Li et al., 2014). Genomic DNA and total RNA were extracted from the cells using QIAGEN AllPrep DNA/RNA/miRNA Universal Kit. See also the Supplemental Experimental Procedures.

### mRNA Sequencing and Data Analysis

mRNA sequencing libraries were prepared using the TruSeq Stranded mRNA Sample Prep Kit (Illumina) and sequenced on HiSeq 2000 (paired-end 50 bp). Sequencing reads were mapped against mm10 reference genome using TopHat (Trapnell et al., 2009). Mapped read counts per annotated gene were collected with HTSeq-Count (Anders et al., 2015). DESeq2 (Love et al., 2014) was then used to identify DEGs ($|\text{fold change}| > 1.2$, $p < 0.01$, and adjusted p value $< 0.25$). See also the Supplemental Experimental Procedures.

### WGBS and Data Processing

Genomic DNA was sonicated to an average size of 200 bp using a Covaris S220 instrument. DNA fragments were end-repaired, adenylated, and ligated to Illumina-compatible adaptors using BIOO NEXTflex Bisulfite-Seq Kit. Bisulfite conversion was performed using EZ DNA Methylation-Lightning Kit (Zymo Research). PCR was then carried out to enrich bisulfite-converted and adaptor-ligated fragments. The libraries were sequenced on Nextseq 500 (paired-end 75 bp). According to the coverage recommendations for WGBS (Ziller et al., 2015), we sequenced five biological replicates per group and achieved an average coverage $>5\times$ per sample.

Sequencing reads were mapped to mm10 reference genome via Bismark (Krueger and Andrews, 2011) with Bowtie as the underlying alignment tool. Duplicates were removed, and any redundant mapped bases due to overlapping mates from the same read pair were trimmed. Furthermore, read cycles showing methylation bias on M-bias plot were trimmed from each mapped hit. The observed bisulfite conversion rate was >99%. See also the Supplemental Experimental Procedures.

### DMR Detection

Rao Scott Likelihood Ratio Test (Rao and Scott, 1981, 1987) was used to identify differentially methylated CpG sites. CpGs were considered differentially methylated only if they had an RSLRT p value $< 0.05$, absolute methylation difference >10%, and total weighted coverage $\geq 10$. Next, differentially methylated CpGs within 5,000 bp were merged into discrete regions. Finally, differential methylation analysis was repeated on the region level using RSLRT. Differentially methylated regions were defined as having a Benjamini-Hochberg adjusted DMR level RSLRT p value $< 0.01$, absolute methylation difference >30% or fold change >5, and containing at least 2 differentially methylated CpGs. See also the Supplemental Experimental Procedures.

### Ribo-Zero RNA-Seq and Data Analysis

RNA-seq libraries were prepared using TruSeq Stranded Total RNA Library Prep Kit with Ribo-Zero H/M/R Gold (Illumina) and sequenced on Illumina NextSeq 500 (paired-end 76 bp). Read pairs were filtered by a mean base quality score >20, followed by adaptor-trimming with CutAdapt, and then they were mapped to mm10 reference genome with spliced transcripts alignment to a reference (STAR) (Dobin et al., 2013). Mapped hits per gene was calculated with Subread featureCounts. DEGs were identified by DESeq2 (Love et al., 2014) with a cutoff of padj $< 0.05$ and $|FC| > 1.2$. See also the Supplemental Experimental Procedures.

### Statistics

Data analysis was performed using Partek and GraphPad Prism. The heatmaps were prepared using Partek. Overlap significance was calculated using hypergeometric test. Body weights and gene expression data were represented as mean ± SEM.

## DATA AND SOFTWARE AVAILABILITY

The accession numbers for the sequencing data reported in this paper are GEO: GSE85731 and GSE100276.

## SUPPLEMENTAL INFORMATION

Supplemental Information includes Supplemental Experimental Procedures, seven figures, and six tables and can be found with this article online at https://doi.org/10.1016/j.celrep.2017.12.071.

## ACKNOWLEDGMENTS

We thank Dr. John Roberts for helpful discussions and critical reading of the manuscript. We thank Epigenomics Core Laboratory at NIEHS for next-generation sequencing. This work was supported by the Intramural Research Program of the NIH, National Institute of Environmental Health Sciences (ES101965 to P.A.W.).

## AUTHOR CONTRIBUTIONS

R.L. and P.A.W. designed this study. R.L., H.G., and D.D. performed experiments. R.L., S.A.G., D.M., and R.S. performed bioinformatics analyses. R.L., P.A.W., B.A.M., and D.R. designed metabolomics experiments. R.L. analyzed metabolomics data. All authors participated in preparation of the manuscript.

## DECLARATION OF INTERESTS

The authors declare no competing interests.

Received: September 6, 2017
Revised: November 16, 2017
Accepted: December 20, 2017
Published: January 16, 2018

## REFERENCES

Amit, I., Citri, A., Shay, T., Lu, Y., Katz, M., Zhang, F., Tarcic, G., Siwak, D., Lahad, J., Jacob-Hirsch, J., et al. (2007). A module of negative feedback regulators defines growth factor signaling. Nat. Genet. *39*, 503–512.

Anders, S., Pyl, P.T., and Huber, W. (2015). HTSeq–a Python framework to work with high-throughput sequencing data. Bioinformatics *31*, 166–169.

Aran, D., and Hellman, A. (2013). DNA methylation of transcriptional enhancers and cancer predisposition. Cell *154*, 11–13.

Aran, D., Sabato, S., and Hellman, A. (2013). DNA methylation of distal regulatory sites characterizes dysregulation of cancer genes. Genome Biol. *14*, R21.

Barbie, D.A., Tamayo, P., Boehm, J.S., Kim, S.Y., Moody, S.E., Dunn, I.F., Schinzel, A.C., Sandy, P., Meylan, E., Scholl, C., et al. (2009). Systematic RNA interference reveals that oncogenic KRAS-driven cancers require TBK1. Nature *462*, 108–112.

Barres, R., Kirchner, H., Rasmussen, M., Yan, J., Kantor, F.R., Krook, A., Näslund, E., and Zierath, J.R. (2013). Weight loss after gastric bypass surgery in human obesity remodels promoter methylation. Cell Rep. *3*, 1020–1027.

Belo, A., Cheng, K., Chahdi, A., Shant, J., Xie, G., Khurana, S., and Raufman, J.P. (2011). Muscarinic receptor agonists stimulate human colon cancer cell migration and invasion. Am. J. Physiol. Gastrointest. Liver Physiol. *300*, G749–G760.

Beyaz, S., Mana, M.D., Roper, J., Kedrin, D., Saadatpour, A., Hong, S.J., Bauer-Rowe, K.E., Xifaras, M.E., Akkad, A., Arias, E., et al. (2016). High-fat diet enhances stemness and tumorigenicity of intestinal progenitors. Nature *531*, 53–58.

Buchwalter, G., Gross, C., and Wasylyk, B. (2004). Ets ternary complex transcription factors. Gene *324*, 1–14.

Carracedo, A., Cantley, L.C., and Pandolfi, P.P. (2013). Cancer metabolism: fatty acid oxidation in the limelight. Nat. Rev. Cancer *13*, 227–232.

Chen, Z., Miao, F., Paterson, A.D., Lachin, J.M., Zhang, L., Schones, D.E., Wu, X., Wang, J., Tompkins, J.D., Genuth, S., et al.; DCCT/EDIC Research Group (2016). Epigenomic profiling reveals an association between persistence of DNA methylation and metabolic memory in the DCCT/EDIC type 1 diabetes cohort. Proc. Natl. Acad. Sci. USA *113*, E3002–E3011.

Cheng, K., Samimi, R., Xie, G., Shant, J., Drachenberg, C., Wade, M., Davis, R.J., Nomikos, G., and Raufman, J.P. (2008). Acetylcholine release by human colon cancer cells mediates autocrine stimulation of cell proliferation. Am. J. Physiol. Gastrointest. Liver Physiol. *295*, G591–G597.

Chin, A.I., Shu, J., Shan Shi, C., Yao, Z., Kehrl, J.H., and Cheng, G. (1999). TANK potentiates tumor necrosis factor receptor-associated factor-mediated c-Jun N-terminal kinase/stress-activated protein kinase activation through the germinal center kinase pathway. Mol. Cell. Biol. *19*, 6665–6672.

Collins, S., Martin, T.L., Surwit, R.S., and Robidoux, J. (2004). Genetic vulnerability to diet-induced obesity in the C57BL/6J mouse: physiological and molecular characteristics. Physiol. Behav. *81*, 243–248.

Dobin, A., Davis, C.A., Schlesinger, F., Drenkow, J., Zaleski, C., Jha, S., Batut, P., Chaisson, M., and Gingeras, T.R. (2013). STAR: ultrafast universal RNA-seq aligner. Bioinformatics *29*, 15–21.

Ferby, I., Reschke, M., Kudlacek, O., Knyazev, P., Pantè, G., Amann, K., Sommergruber, W., Kraut, N., Ullrich, A., Fässler, R., and Klein, R. (2006). Mig6 is a negative regulator of EGF receptor-mediated skin morphogenesis and tumor formation. Nat. Med. *12*, 568–573.

Ferlay, J., Soerjomataram, I., Dikshit, R., Eser, S., Mathers, C., Rebelo, M., Parkin, D.M., Forman, D., and Bray, F. (2015). Cancer incidence and mortality worldwide: sources, methods and major patterns in GLOBOCAN 2012. Int. J. Cancer *136*, E359–E386.

Gulhane, M., Murray, L., Lourie, R., Tong, H., Sheng, Y.H., Wang, R., Kang, A., Schreiber, V., Wong, K.Y., Magor, G., et al. (2016). High Fat Diets Induce Colonic Epithelial Cell Stress and Inflammation that is Reversed by IL-22. Sci. Rep. *6*, 28990.

Hanafusa, H., Torii, S., Yasunaga, T., and Nishida, E. (2002). Sprouty1 and Sprouty2 provide a control mechanism for the Ras/MAPK signalling pathway. Nat. Cell Biol. *4*, 850–858.

Hanahan, D., and Weinberg, R.A. (2011). Hallmarks of cancer: the next generation. Cell *144*, 646–674.

Heinz, S., Benner, C., Spann, N., Bertolino, E., Lin, Y.C., Laslo, P., Cheng, J.X., Murre, C., Singh, H., and Glass, C.K. (2010). Simple combinations of lineage-determining transcription factors prime cis-regulatory elements required for macrophage and B cell identities. Mol. Cell *38*, 576–589.

Hirayama, A., Kami, K., Sugimoto, M., Sugawara, M., Toki, N., Onozuka, H., Kinoshita, T., Saito, N., Ochiai, A., Tomita, M., et al. (2009). Quantitative metabolome profiling of colon and stomach cancer microenvironment by capillary electrophoresis time-of-flight mass spectrometry. Cancer Res. *69*, 4918–4925.

Hu, J., Locasale, J.W., Bielas, J.H., O'Sullivan, J., Sheahan, K., Cantley, L.C., Vander Heiden, M.G., and Vitkup, D. (2013). Heterogeneity of tumor-induced gene expression changes in the human metabolic network. Nat. Biotechnol. *31*, 522–529.

Hue, L., and Taegtmeyer, H. (2009). The Randle cycle revisited: a new head for an old hat. Am. J. Physiol. Endocrinol. Metab. *297*, E578–E591.

Imamura, T., Takase, M., Nishihara, A., Oeda, E., Hanai, J., Kawabata, M., and Miyazono, K. (1997). Smad6 inhibits signalling by the TGF-beta superfamily. Nature *389*, 622–626.

Jackson, S.J., Andrews, N., Ball, D., Bellantuono, I., Gray, J., Hachoumi, L., Holmes, A., Latcham, J., Petrie, A., Potter, P., et al. (2017). Does age matter? The impact of rodent age on study outcomes. Lab. Anim. *51*, 160–169.

Kaiko, G.E., Ryu, S.H., Koues, O.I., Collins, P.L., Solnica-Krezel, L., Pearce, E.J., Pearce, E.L., Oltz, E.M., and Stappenbeck, T.S. (2016). The Colonic Crypt Protects Stem Cells from Microbiota-Derived Metabolites. Cell *165*, 1708–1720.

Krueger, F., and Andrews, S.R. (2011). Bismark: a flexible aligner and methylation caller for Bisulfite-Seq applications. Bioinformatics *27*, 1571–1572.

Lao, V.V., and Grady, W.M. (2011). Epigenetics and colorectal cancer. Nat. Rev. Gastroenterol. Hepatol. *8*, 686–700.

Li, L., and Guan, K.L. (2013). Microtubule-associated protein/microtubule affinity-regulating kinase 4 (MARK4) is a negative regulator of the mammalian target of rapamycin complex 1 (mTORC1). J. Biol. Chem. *288*, 703–708.

Li, R., Grimm, S.A., Chrysovergis, K., Kosak, J., Wang, X., Du, Y., Burkholder, A., Janardhan, K., Mav, D., Shah, R., et al. (2014). Obesity, rather than diet, drives epigenomic alterations in colonic epithelium resembling cancer progression. Cell Metab. *19*, 702–711.

Liu, S., Nheu, T., Luwor, R., Nicholson, S.E., and Zhu, H.J. (2015). SPSB1, a Novel Negative Regulator of the Transforming Growth Factor-β Signaling Pathway Targeting the Type II Receptor. J. Biol. Chem. *290*, 17894–17908.

Lockyer, P.J., Kupzig, S., and Cullen, P.J. (2001). CAPRI regulates Ca(2+)-dependent inactivation of the Ras-MAPK pathway. Curr. Biol. *11*, 981–986.

Love, M.I., Huber, W., and Anders, S. (2014). Moderated estimation of fold change and dispersion for RNA-seq data with DESeq2. Genome Biol. *15*, 550.

Mandl, M., Slack, D.N., and Keyse, S.M. (2005). Specific inactivation and nuclear anchoring of extracellular signal-regulated kinase 2 by the inducible dual-specificity protein phosphatase DUSP5. Mol. Cell. Biol. *25*, 1830–1845.

Nakao, A., Afrakhte, M., Morén, A., Nakayama, T., Christian, J.L., Heuchel, R., Itoh, S., Kawabata, M., Heldin, N.E., Heldin, C.H., and ten Dijke, P. (1997). Identification of Smad7, a TGFbeta-inducible antagonist of TGF-beta signalling. Nature *389*, 631–635.

Newmark, H.L., Yang, K., Kurihara, N., Fan, K., Augenlicht, L.H., and Lipkin, M. (2009). Western-style diet-induced colonic tumors and their modulation by calcium and vitamin D in C57Bl/6 mice: a preclinical model for human sporadic colon cancer. Carcinogenesis *30*, 88–92.

Pombo, C.M., Kehrl, J.H., Sánchez, I., Katz, P., Avruch, J., Zon, L.I., Woodgett, J.R., Force, T., and Kyriakis, J.M. (1995). Activation of the SAPK pathway by the human STE20 homologue germinal centre kinase. Nature *377*, 750–754.

Rao, J.N.K., and Scott, A.J. (1981). The Analysis of Categorical Data from Complex Sample Surveys: Chi-Squared Tests for Goodness of Fit and Independence in Two-Way Tables. J. Am. Stat. Assoc. *76*, 221–230.

Rao, J.N.K., and Scott, A.J. (1987). On Simple Adjustments to Chi-Square Tests with Sample Survey Data. Ann. Stat. *15*, 385–397.

Renehan, A.G., Tyson, M., Egger, M., Heller, R.F., and Zwahlen, M. (2008). Body-mass index and incidence of cancer: a systematic review and meta-analysis of prospective observational studies. Lancet *371*, 569–578.

Renner, F., and Schmitz, M.L. (2009). Autoregulatory feedback loops terminating the NF-kappaB response. Trends Biochem. Sci. *34*, 128–135.

Roediger, W.E. (1982). Utilization of nutrients by isolated epithelial cells of the rat colon. Gastroenterology *83*, 424–429.

Schratt, G., Weinhold, B., Lundberg, A.S., Schuck, S., Berger, J., Schwarz, H., Weinberg, R.A., Rüther, U., and Nordheim, A. (2001). Serum response factor is required for immediate-early gene activation yet is dispensable for proliferation of embryonic stem cells. Mol. Cell. Biol. *21*, 2933–2943.

Sharma, U., and Rando, O.J. (2017). Metabolic Inputs into the Epigenome. Cell Metab. *25*, 544–558.

Shembade, N., Ma, A., and Harhaj, E.W. (2010). Inhibition of NF-kappaB signaling by A20 through disruption of ubiquitin enzyme complexes. Science *327*, 1135–1139.

Shi, C.S., Tuscano, J.M., Witte, O.N., and Kehrl, J.H. (1999). GCKR links the Bcr-Abl oncogene and Ras to the stress-activated protein kinase pathway. Blood *93*, 1338–1345.

Shyh-Chang, N., Daley, G.Q., and Cantley, L.C. (2013). Stem cell metabolism in tissue development and aging. Development *140*, 2535–2547.

Siegel, R.L., Fedewa, S.A., Anderson, W.F., Miller, K.D., Ma, J., Rosenberg, P.S., and Jemal, A. (2017). Colorectal Cancer Incidence Patterns in the United States, 1974-2013. J. Natl. Cancer Inst. *109*, djw322.

Slack, D.N., Seternes, O.M., Gabrielsen, M., and Keyse, S.M. (2001). Distinct binding determinants for ERK2/p38alpha and JNK map kinases mediate catalytic activation and substrate selectivity of map kinase phosphatase-1. J. Biol. Chem. *276*, 16491–16500.

Spruijt, C.G., and Vermeulen, M. (2014). DNA methylation: old dog, new tricks? Nat. Struct. Mol. Biol. *21*, 949–954.

Trapnell, C., Pachter, L., and Salzberg, S.L. (2009). TopHat: discovering splice junctions with RNA-Seq. Bioinformatics *25*, 1105–1111.

Tullai, J.W., Schaffer, M.E., Mullenbrock, S., Sholder, G., Kasif, S., and Cooper, G.M. (2007). Immediate-early and delayed primary response genes are distinct in function and genomic architecture. J. Biol. Chem. *282*, 23981–23995.

Tuominen, I., Al-Rabadi, L., Stavrakis, D., Karagiannides, I., Pothoulakis, C., and Bugni, J.M. (2013). Diet-induced obesity promotes colon tumor development in azoxymethane-treated mice. PLoS ONE *8*, e60939.

Wang, S., Sun, H., Ma, J., Zang, C., Wang, C., Wang, J., Tang, Q., Meyer, C.A., Zhang, Y., and Liu, X.S. (2013). Target analysis by integration of transcriptome and ChIP-seq data with BETA. Nat. Protoc. *8*, 2502–2515.

Wang, A., Yue, F., Li, Y., Xie, R., Harper, T., Patel, N.A., Muth, K., Palmer, J., Qiu, Y., Wang, J., et al. (2015). Epigenetic priming of enhancers predicts developmental competence of hESC-derived endodermal lineage intermediates. Cell Stem Cell *16*, 386–399.

Yoshimura, A. (1998). The CIS/JAB family: novel negative regulators of JAK signaling pathways. Leukemia *12*, 1851–1857.

Yue, F., Cheng, Y., Breschi, A., Vierstra, J., Wu, W., Ryba, T., Sandstrom, R., Ma, Z., Davis, C., Pope, B.D., et al.; Mouse ENCODE Consortium (2014). A comparative encyclopedia of DNA elements in the mouse genome. Nature *515*, 355–364.

Zhang, J., Wu, G., Chapkin, R.S., and Lupton, J.R. (1998). Energy metabolism of rat colonocytes changes during the tumorigenic process and is dependent on diet and carcinogen. J. Nutr. *128*, 1262–1269.

Zhao, M., Kim, P., Mitra, R., Zhao, J., and Zhao, Z. (2016). TSGene 2.0: an updated literature-based knowledgebase for tumor suppressor genes. Nucleic Acids Res. *44* (*D1*), D1023–D1031.

Ziller, M.J., Hansen, K.D., Meissner, A., and Aryee, M.J. (2015). Coverage recommendations for methylation analysis by whole-genome bisulfite sequencing. Nat. Methods *12*, 230–232.

Cell Reports, Volume *22*

## Supplemental Information

# Transcriptome and DNA Methylome Analysis in a Mouse Model of Diet-Induced Obesity Predicts Increased Risk of Colorectal Cancer

**Ruifang Li, Sara A. Grimm, Deepak Mav, Haiwei Gu, Danijel Djukovic, Ruchir Shah, B. Alex Merrick, Daniel Raftery, and Paul A. Wade**

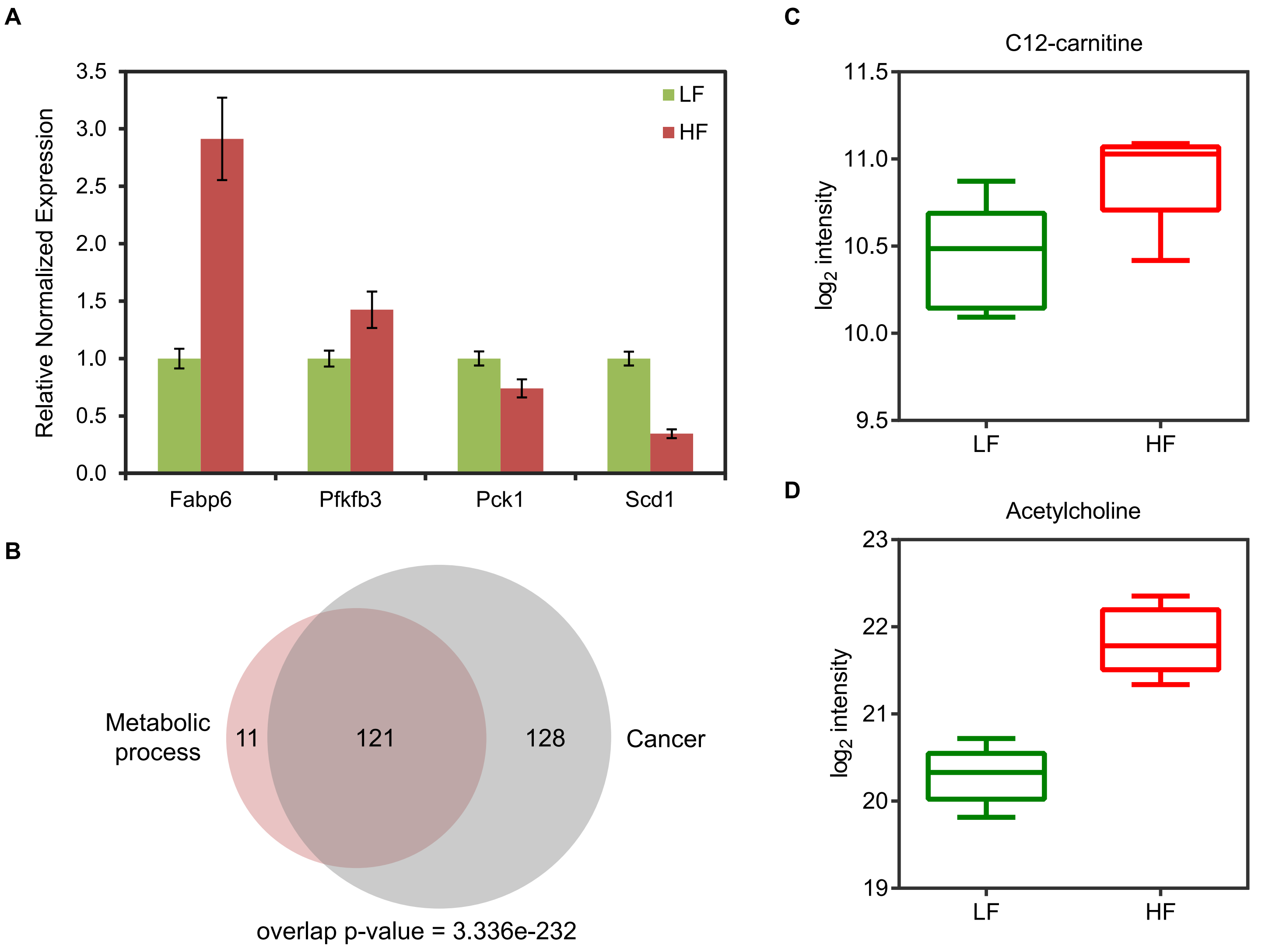


Figure S1. Differentially expressed metabolic genes and altered metabolites in colonic epithelium of young obese mice, related to Figure 1. (A) Validation of RNA-Seq data using real-time RT-PCR. Gene expression levels were normalized to that of Gapdh. Data are represented as mean ± SEM (n=5). (B) Overlap of dysregulated genes involved in metabolic processes with dysregulated cancer-related genes in young obese mice. Overlap p-value was calculated using hypergeometric test. (C and D) Levels of C12-carnitine and acetylcholine in colonic epithelium from young obese and control mice. Y-axis indicates the area of peak from targeted metabolome analysis.

Figure S2

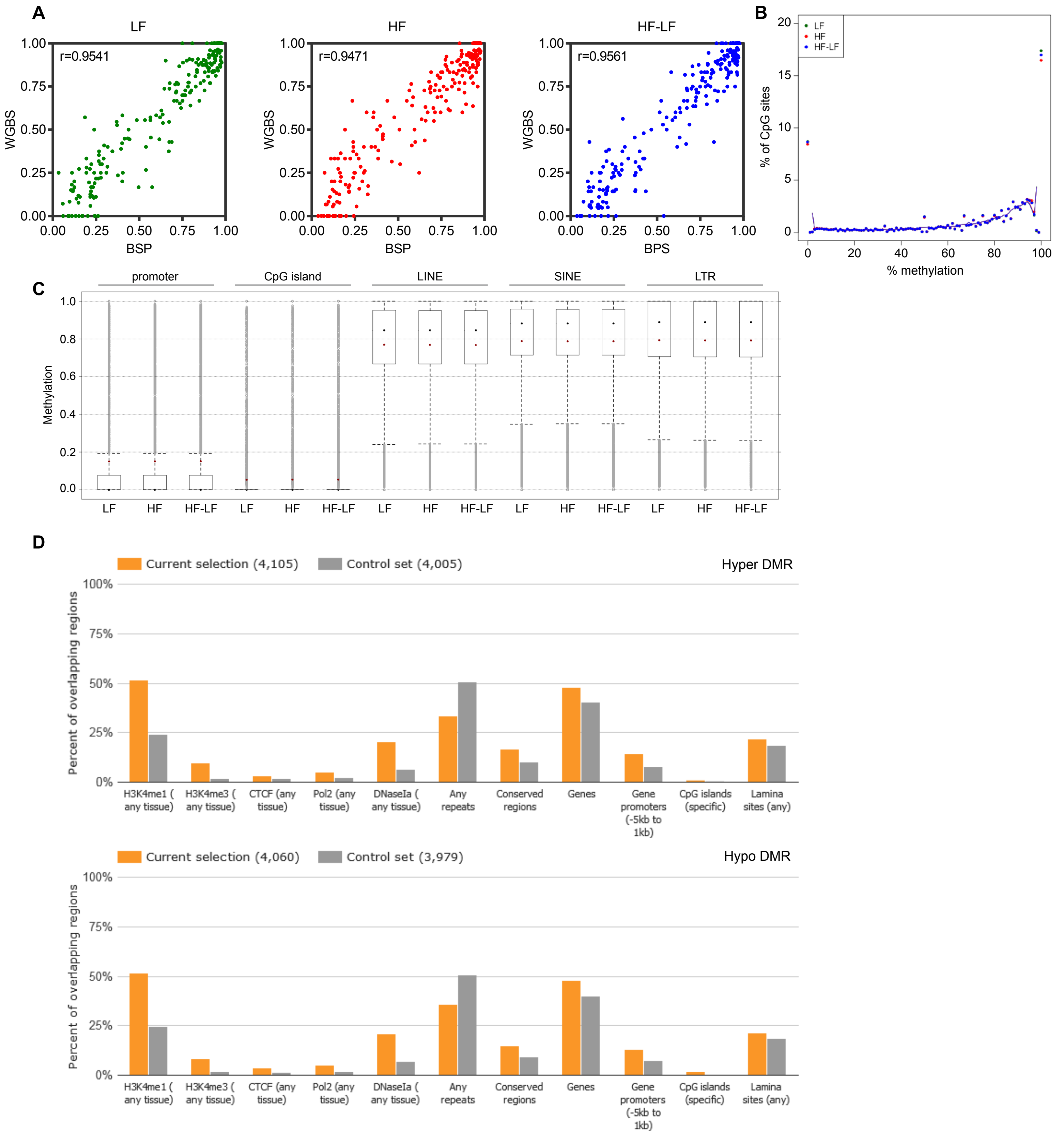


Figure S2, related to Figure 2. (A) WGBS and BSP-Seq showed strong correlation of the methylation levels of 233 randomly selected CpG sites. (B) Bimodal distribution of DNA methylation at individual CpG sites. (C) Global methylation levels of promoters, CpG islands, and repetitive elements (LINE, SINE, and LTR). The blue and red dots represent the median and the mean, respectively. (D) Percentage of DMRs or matched control regions overlapping with annotated genomic features and experimentally defined functional genomic elements. Upper panel: hyper DMRs; lower panel: hypo DMRs.

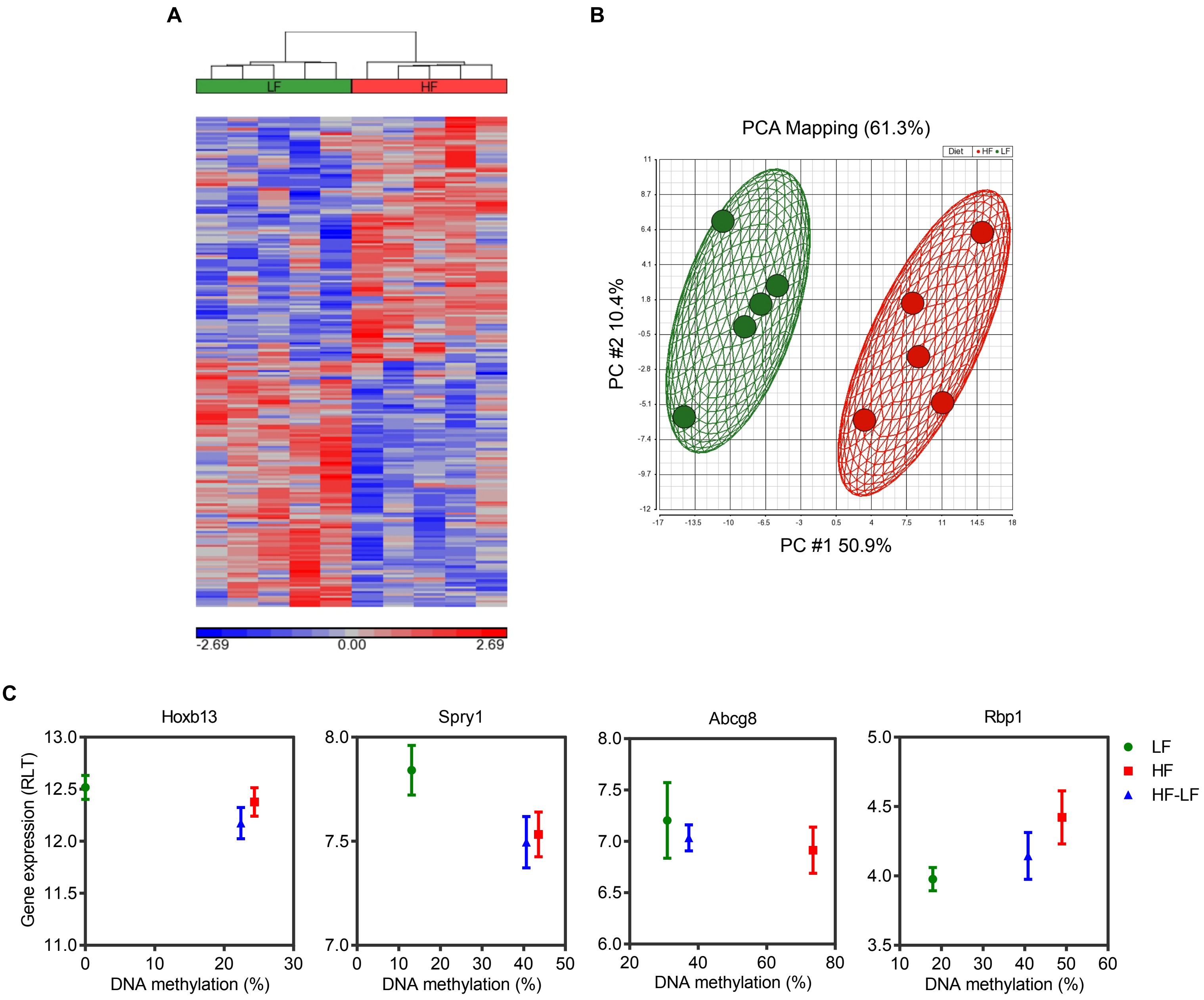


Figure S3. DMR-target genes without significant differential expression, related to Figure 3. (A) Heatmap depicts the standardized expression levels of 213 non-DEG-overlapping DMR-target genes in control mice (LF) and obese mice (HF). (B) Obese mice and control mice were well separated by Principal Components Analysis (PCA), based on expression levels of 213 non-DEG-overlapping DMR-target genes. (C) Examples of correlation between DNA methylation and gene expression.

Figure S4

**A**

| | Consensus binding sites | p-value (DMR motif enrichment) | p-value (DEG upstream regulator) |
|---|---|---|---|
| E2F4 | GGCGGGAAAH | 1.00E-08 | 2.14E-19 |
| E2f | TTSGCGCGAAAA | 1.00E-03 | 2.23E-08 |
| ELK4 | NRYTTCCGGY | 1.00E-13 | 2.62E-10 |
| ELK1 | HACTTCCGGY | 1.00E-06 | 1.62E-05 |

**B**

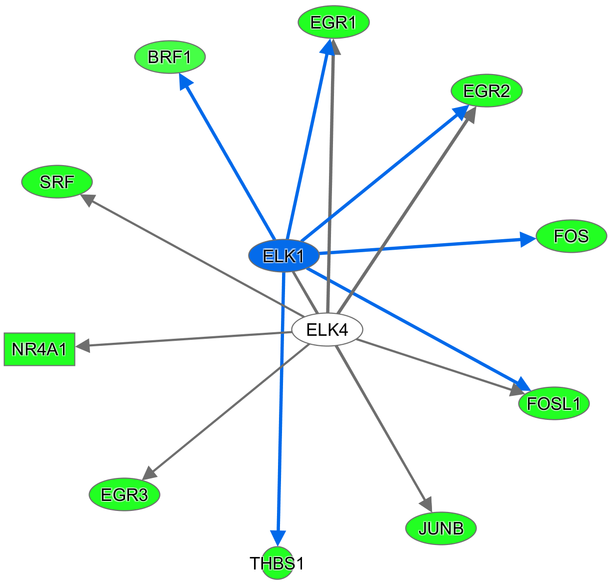


**C**

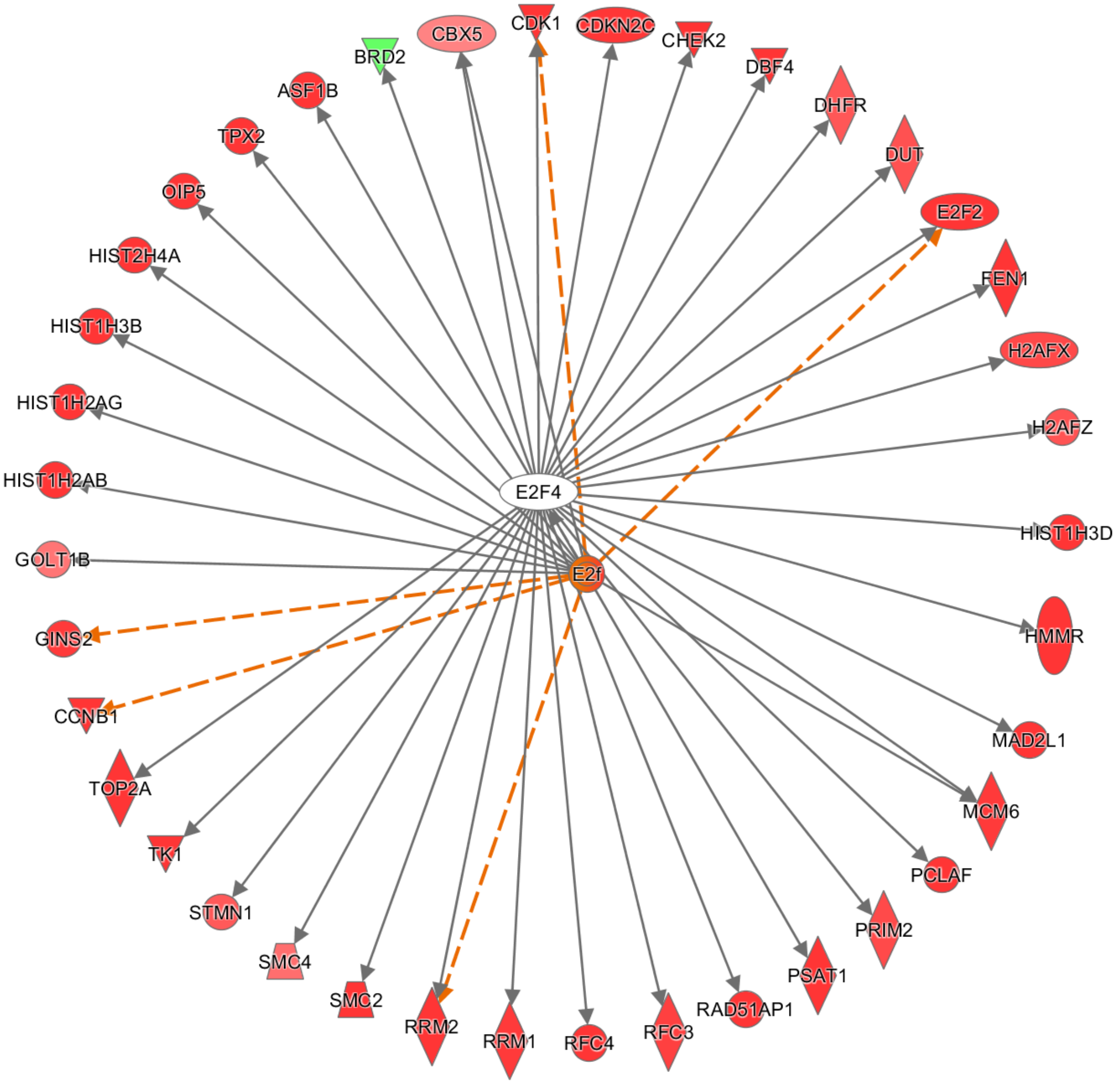


Figure S4. Examples of transcription factors with binding motifs enriched at DMRs and also serving as upstream regulators of DEGs in aged obese mice, related to Figure 4. (A) The p-values from HOMER motif enrichment analysis and IPA upstream regulator analysis were shown for the following transcription factors: E2F, ELK1, and ELK4. (B and C) Differential expression of the target genes of E2F, ELK1 and ELK4. Red indicates up-regulation while Green indicates down-regulation of genes in aged obese mice relative to age-matched controls.

Figure S5

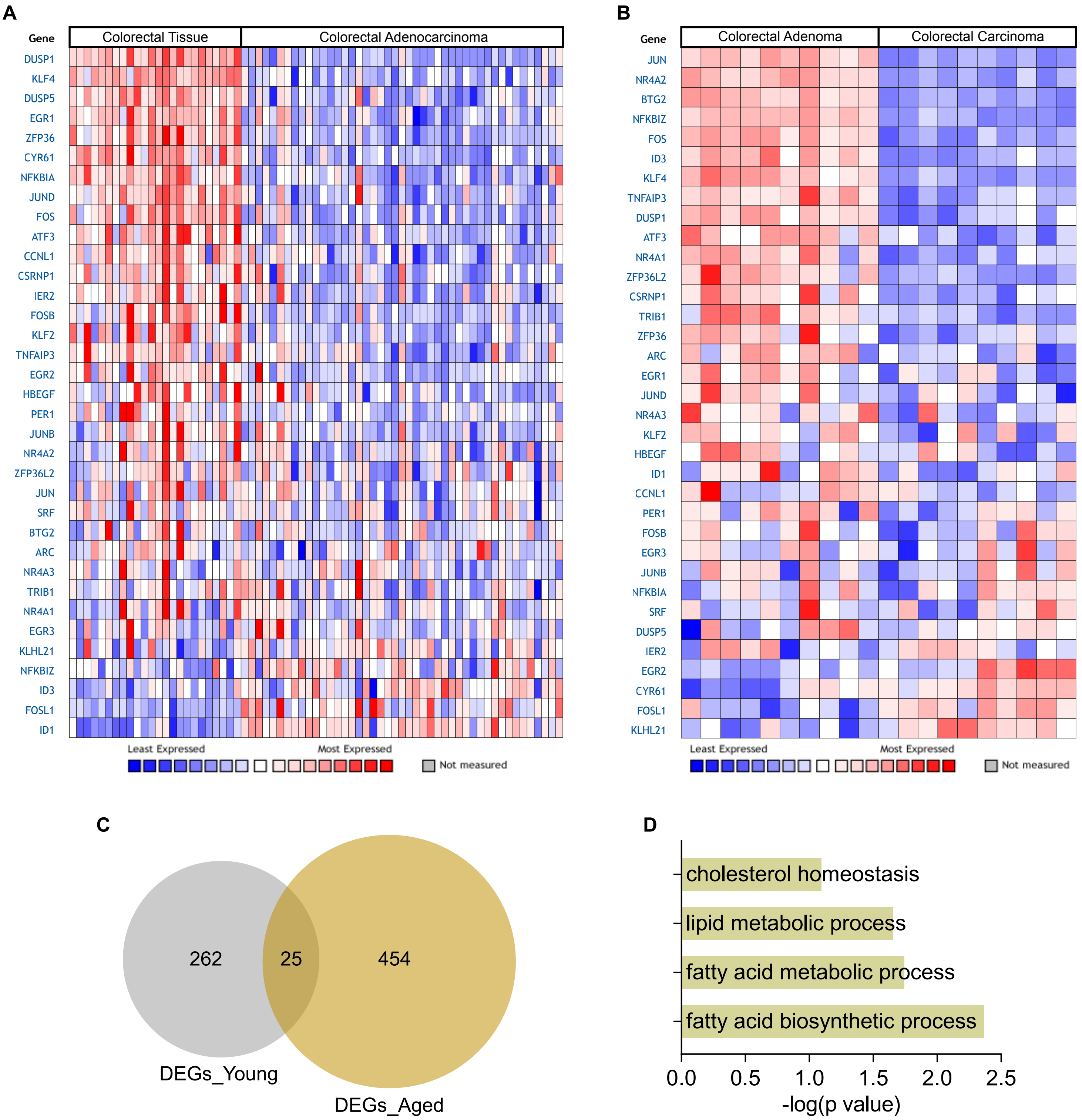


Figure S5, related to Figure 5. (A and B) Heatmaps display relative expression of the 35 primary response genes in human colorectal cancer compared with normal colorectal tissue (A) and benign colorectal tumor (B). (C) Overlap of obesity-related DEGs in young and aged mice. (D) GO_BP terms enriched with consistently altered genes in both young and aged obese mice.

Figure S6

A

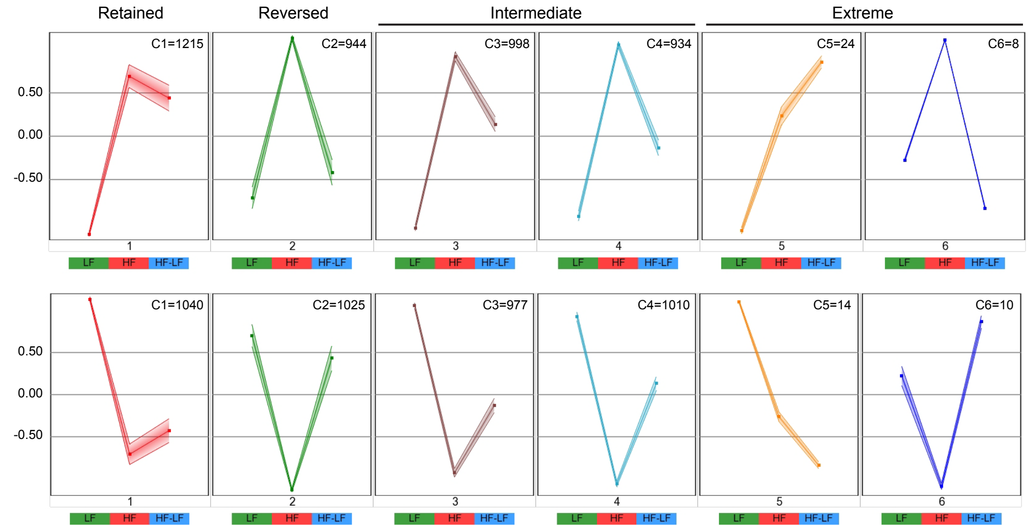


B

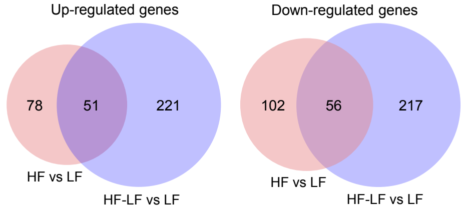


C

| Name | p-value | #Molecules |
|---|---|---|
| Diseases and Disorders | | |
| Cancer | 1.45E-02 - 2.76E-09 | 102 |
| Gastrointestinal Disease | 1.50E-02 - 2.76E-09 | 96 |
| Organismal Injury and Abnormalities | 1.50E-02 - 2.76E-09 | 102 |
| Developmental Disorder | 1.50E-02 - 2.51E-05 | 15 |
| Hereditary Disorder | 1.50E-02 - 2.51E-05 | 27 |
| Molecular and Cellular Functions | | |
| Lipid Metabolism | 1.50E-02 - 1.01E-06 | 31 |
| Small Molecule Biochemistry | 1.50E-02 - 1.01E-06 | 38 |
| Molecular Transport | 1.50E-02 - 1.72E-04 | 29 |
| Carbohydrate Metabolism | 1.50E-02 - 2.33E-04 | 17 |
| Cell Morphology | 1.50E-02 - 4.97E-04 | 29 |

D

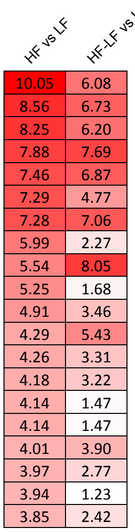

| HF vs LF | HF-LF vs LF | |
|---|---|---|
| 10.05 | 6.08 | monocarboxylic acid metabolic process |
| 8.56 | 6.73 | lipid metabolic process |
| 8.25 | 6.20 | carboxylic acid metabolic process |
| 7.88 | 7.69 | cellular lipid metabolic process |
| 7.46 | 6.87 | oxoacid metabolic process |
| 7.29 | 4.77 | fatty acid metabolic process |
| 7.28 | 7.06 | organic acid metabolic process |
| 5.99 | 2.27 | monocarboxylic acid biosynthetic process |
| 5.54 | 8.05 | small molecule metabolic process |
| 5.25 | 1.68 | organic hydroxy compound metabolic process |
| 4.91 | 3.46 | lipid biosynthetic process |
| 4.29 | 5.43 | hormone metabolic process |
| 4.26 | 3.31 | acylglycerol metabolic process |
| 4.18 | 3.22 | neutral lipid metabolic process |
| 4.14 | 1.47 | carboxylic acid biosynthetic process |
| 4.14 | 1.47 | organic acid biosynthetic process |
| 4.01 | 3.90 | lipid localization |
| 3.97 | 2.77 | unsaturated fatty acid metabolic process |
| 3.94 | 1.23 | fatty acid biosynthetic process |
| 3.85 | 2.42 | triglyceride metabolic process |

-log10(p-value)

E

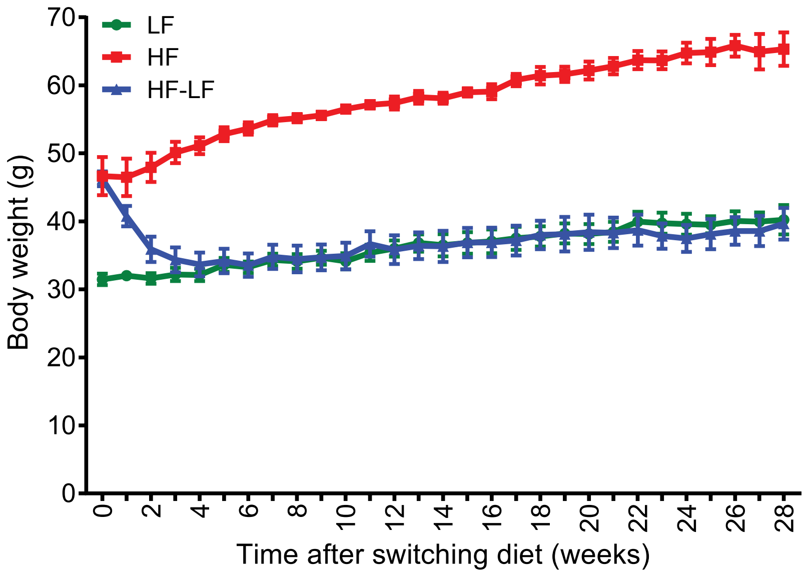


F

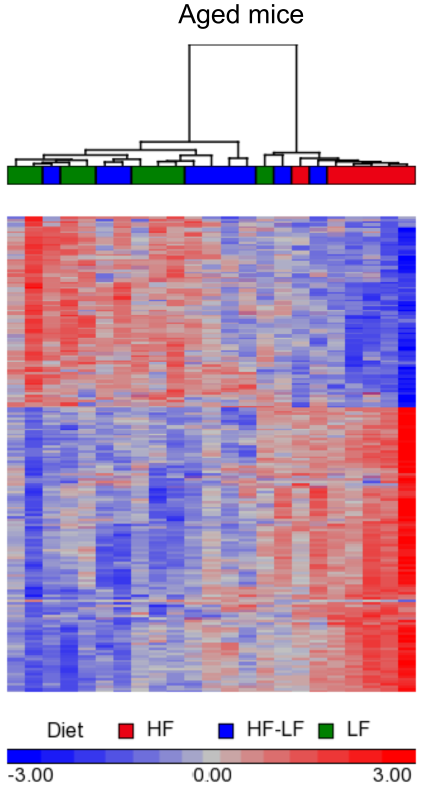


Figure S6. Obesity-related changes in DNA methylation and gene expression were substantially preserved after short-term weight loss but were largely reversed after long-term weight loss, related to Figure 6. (A) Obesity-associated DMRs were clustered into 6 groups based on their methylation levels in formerly obese mice (short-term). The number of DMRs in each group was shown in the upper-right corner. (B) Overlap of DEGs in formerly obese mice (short-term) with DEGs in obese mice. (C) Ingenuity Pathway Analysis of commonly dysregulated genes. The top five scoring hits in each functional category are shown, together with p-values and the number of commonly dysregulated genes in enriched terms. (D) Comparison of enriched GO terms in formerly obese mice (short-term) with that in obese mice. Colors indicate the significance of p-values with darker red being more significant and white being non-significant. The numbers are -log10 of p values. (E) Weekly body weight of control (LF), obese (HF), and formerly obese (HF-LF) mice from the long-term weight loss study (diet-switching for 28 weeks). Data are represented as mean ± SEM (n=4 or 5). (F) Hierarchical clustering of samples on obesity-related differentially expressed genes in aged mice. Ward's method was used with Pearson's Dissimilarity calculated using standardized gene expression levels.

Figure S7

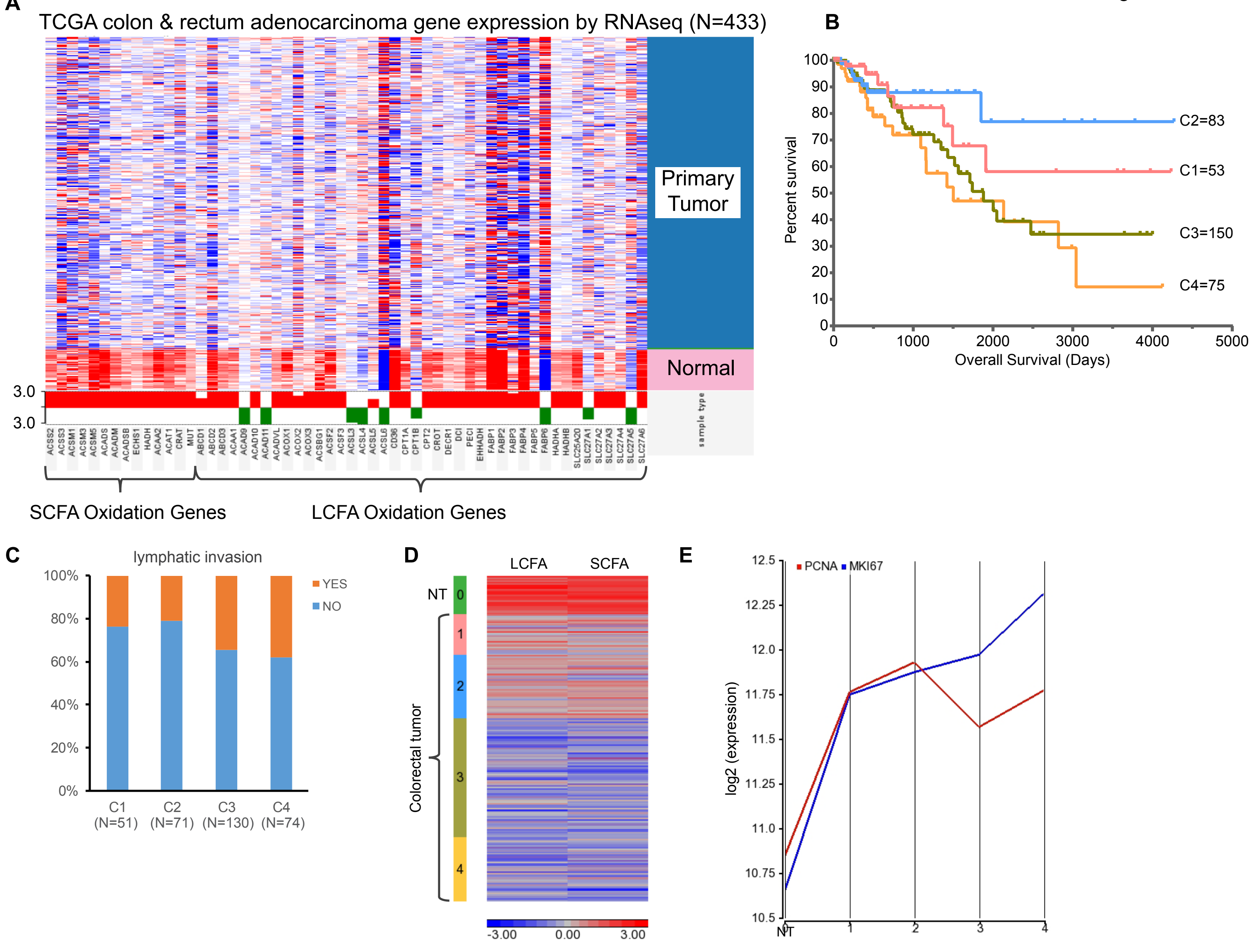


Figure S7, related to Figure 7. (A) Heatmap displays normalized expression of short- and long-chain fatty acid oxidation genes in primary colorectal cancer and adjacent normal tissue. Red correlates with higher expression. The statistical track, which is displayed under the gene heatmap, shows the -log (p-value) for each gene comparing primary colorectal cancer with adjacent normal tissue. (B) Kaplan-Meier plot depicts the overall survival of CRC patients stratified by the metabolic features of their tumor samples. (C) The percentages of CRC patients in the four defined groups harboring lymphatic invasion. (D) Heatmap displays normalized ssGSEA enrichment scores of gene sets specific for SCFA and LCFA metabolism. (E) Average expression levels of PCNA and MKI67 in CRC samples of each group.

## Supplemental Experimental Procedures

### Isolation of Colonic Epithelium

Colonic epithelium was isolated as previously described (Li et al., 2014). Briefly, the entire colon was removed from each mouse, flushed with PBS without calcium and magnesium, and then cut open longitudinally. After being washed two more times, the colon was placed in PBS with 5 mM EDTA to incubate at 37 °C for 15 min on a rotator. Finally, the colon tissue was removed and colonic epithelium was pelleted and washed twice with PBS. The cells were used immediately or snap frozen in liquid nitrogen and stored at -80 °C.

### DNA and RNA Preparation

Genomic DNA and total RNA were isolated using the Qiagen AllPrep DNA/RNA/miRNA Universal Kit according to the manufacturer's instructions. The concentrations of DNA and RNA were measured using the NanoDrop spectrophotometer. The quality of DNA and RNA was assessed by agarose gel electrophoresis and Bioanalyzer, respectively.

### Real-Time RT-PCR

cDNA was generated using the iScript™ cDNA Synthesis Kit (Biorad, catalog #170-8890). Relative expression levels of Fabp6, Pfkfb3, Pck1, and Scd1 were determined by real-time PCR assays using TaqMan® Gene Expression Assay kits (Applied Biosystems). Gene expression levels were normalized to that of Gapdh.

### mRNA Sequencing and Data Analysis

mRNA sequencing libraries were prepared using the TruSeq Stranded mRNA Sample Prep Kit (Illumina, #RS-122-2103). Starting with 100 ng of total RNA, polyadenylated RNA (primarily mRNA) was selected and purified using oligo-dT conjugated magnetic beads. The mRNA was then chemically fragmented and converted into single-stranded cDNA using reverse transcriptase and random hexamer primers, with the addition of Actinomycin D to suppress DNA-dependent synthesis. Double-stranded cDNA was created by removing the RNA template and synthesizing the second strand in the presence of dUTP in place of dTTP. A single 'A' nucleotide was added to the 3' end of double-stranded cDNA to facilitate ligation of sequencing adapters, which contain a single 'T' nucleotide overhang. Adapter-ligated cDNA was amplified using polymerase chain reaction (PCR) to increase the amount of sequence-ready library. During PCR amplification, the polymerase stalls when it encounters a uracil, rendering the second strand a poor template. Accordingly, only the first strand was used as a template, thereby preserving the strand information. Final cDNA libraries were analyzed for size distribution using Agilent Bioanalyzer (DNA 1000 kit, Agilent # 5067-1504), quantitated by qPCR (KAPA Library Quant Kit, KAPA Biosystems # KK4824), and then normalized to 2 nM prior to sequencing on HiSeq 2000 (paired-end 50 bp).

General quality control checks were performed with FastQC v0.14.0 (http://www.bioinformatics.babraham.ac.uk/projects/fastqc/). Each dataset was filtered to retain only sequences for which both reads in a pair had an average base quality score of at least 20. Filtered datasets were mapped against the mm10 reference genome using TopHat (Trapnell et al., 2009) v2.0.4 (parameters --b2-sensitive --library-type fr-firststrand -g 10 --mate-inner-dist 40 --mate-std-dev 50). Mapped read counts per annotated gene were collected with HTSeq-Count (Anders et al., 2015); the gene models were generated from RefSeq annotations downloaded from the UCSC Genome Browser as of April 6, 2014. DESeq2 (Love et al., 2014) v1.2.10 was then used to identify differentially expressed genes ($|\text{fold change}| > 1.2$, $p < 0.01$, and adjusted p-value $< 0.25$).

### Bisulfite PCR Deep Sequencing

Bisulfite PCR primers were designed using website: http://www.urogene.org/methprimer/. Genomic DNA was bisulfite converted using EZ DNA Methylation-Lightning™ Kit (Zymo Research Corporation) before PCR amplification. Amplicons from a single sample were pooled and individually indexed using TruSeq™ RNA Sample Preparation kit (Illumina) to create a multiplex library prior to sequencing (250 bp PE) on an Illumina MiSeq instrument using v3 chemistry.

### WGBS Data Processing

General quality control checks were performed with FastQC (http://www.bioinformatics.babraham.ac.uk/projects/fastqc/). Filtered sequencing datasets were mapped to a reference genome via Bismark (Krueger and Andrews, 2011) v0.14.0 (parameters -X 10000 --non_bs_mm -n 2 -l 50 -e 70 --chunkmbs 1024), using Bowtie (Langmead et al., 2009) v0.12.8 as the underlying alignment tool. The reference genome contains the genome sequence of Enterobacteria phage λ in addition to all chromosomes of the mm10 assembly (GRCm38). Mappings for all datasets generated from the same library were merged, and duplicates were removed via the deduplication tool included in the Bismark package. Mapped reads were then separated by genome (mouse or phage λ) and by source strand (plus or minus). Any redundant mapped bases due to overlapping mates from the same read pair were trimmed to avoid bias in quantification of methylation status. Furthermore, read cycles where methylation bias was observed (typically at the 5' end and sometimes also at the 3' end of reads) were trimmed from each mapped hit. Precise boundaries of the trim positions were gleaned from the M-bias plot generated by Bismark's bismark_methylation_extractor tool; the M-bias plot for each sample/sequencing run combination was evaluated independently. Read pairs mapped to phage λ were used as a QC assessment to confirm that the observed bisulfite conversion rate was > 99%. Read pairs mapped to the mouse reference genome were used for downstream analysis.

## DMR Detection

Pairwise comparison was first carried out on a single CpG level. For each CpG in the mouse genome (mm10), we obtained methylated and unmethylated cytosine counts from the aligned WGBS data and performed Rao Scott Likelihood Ratio Test to identify statistically significant differential methylation. This statistical test, typically used for association analysis in complex sampling survey analysis (Rao and Scott, 1981, 1984, 1987), is simply a design-corrected version of the standard likelihood ratio test used in multinomial contingency tables, but it allows design-based reweighting of the observed counts. Compared to alternative parametric techniques designed for over-dispersed data such as beta-binomial regression, this test is not computationally intensive, making it feasible to use with the millions of CpGs resulting from whole genome studies. The sampling weight for each replicate was computed using the total number of methylated/unmethylated base counts across all CpG dinucleotides. CpGs were considered differentially methylated only if RSLRT P-value < 0.05, absolute methylation difference > 10%, and total weighted coverage ≥ 10 in at least one cohort. Subsequently, differentially methylated CpGs within 5000 bp were merged into discrete regions. Next, differential methylation analysis was repeated on the region level using RSLRT. Differentially methylated regions were defined as having a Benjamini-Hochberg adjusted DMR level RSLRT P-value less than 0.01, absolute methylation difference more than 30% or fold change more than 5, and containing at least 2 differentially methylated CpGs.

## BETA Analysis and functional annotation of DMRs

Binding and expression target analysis (BETA) (Wang et al., 2013) was run on the web server (http://cistrome.org/ap/) using default parameters. Overlap of DMRs with annotated genomic features and experimentally defined functional genomic elements was performed using EpiExplorer (Halachev et al., 2012), which is a web tool allowing users to explore large-scale genomic datasets in search of interesting functional associations with user-defined regions. When custom region set was uploaded, EpiExplorer automatically generated randomized control regions, simply by reshuffling the genomic positions of all regions in the user-uploaded dataset. The randomized control set was automatically included as a reference (in grey) in all bar charts to assess whether the association between custom region set and an annotation attribute is biologically relevant. Genomic coordinates of DMRs were converted from mm10 to mm9 by CrossMap (Zhao et al., 2014), since EpiExplorer currently supports only mm9. The selected overlap criterion is any overlap. The results did not change with at least 10% overlap or at least 50% overlap.

## HOMER Motif Analysis

Each individual CpG site within DMRs was extracted and extended on both sides for 10bp; the resulting regions were subsequently merged, if there was any overlap. The HOMER (v4.9.1) motif analysis tool (Heinz et al., 2010) was used to identify known transcription factor motifs enriched at the query sequences relative to a size and GC-matched genomic background.

## Untargeted Lipidomics Analysis

After 100 µL 1x PBS was added to the cell pellets, the mixture was sonicated in an ice/water bath for 15 min. Then 500 µL chloroform:methanol (2:1, V:V) was added and the mixture was incubated on dry ice for 30 min. After centrifuging at 14,000 RPM for 5 min, the lower phase was collected for lipidomics analysis. The lipidomics data were collected using a standard metabolic profiling MS method in the NW-MRC (Buas et al., 2016). LC-QTOF-MS experiments were performed using an Agilent 1200 SL LC system coupled online with an Agilent 6520 Q-TOF mass spectrometer (Agilent Technologies, Santa Clara, CA). Each prepared sample (8 µL for positive ESI ionization, 12 µL for negative ESI ionization) was injected onto an Agilent Zorbax 300 SB-C8 column (2.1× 50mm, 1.8-µm), which was heated to 50 °C. The flow rate was 0.4 mL/min. Mobile phase A was 5 mM ammonium acetate and 0.1% formic acid in water, and mobile phase B was 5% water in ACN containing 5 mM ammonium acetate and 0.1% formic acid. The mobile phase composition was kept isocratic at 35% B for 1 min, and was increased to 95% B in 19 min; after another 10 min at 95% B, the mobile phase composition was returned to 35% B. The ESI voltage was 3.8 kV. The Q-TOF MS spectrometer was calibrated prior to each batch run and a reference channel infusing the standard reference mixture (G1969-85001, Agilent Technologies, Santa Clara, CA) was used during the experiments to ensure mass accuracy. The mass scan range was 100–1600 Da, and the acquisition rate was 1.5 spectra/s. The Q-TOF data were extracted using Agilent MassHunter Qualitative Analysis (version B.07.00) and Mass Profiler Professional (MPP, version B.13.00) software. The absolute intensity threshold for the LC–Q-TOF data extraction was 1000, and the mass accuracy limit was set to 10 ppm.

**Targeted Metabolomics Analysis**

We used methanol:$H_2O$ (8:2) to extract aqueous metabolites. The LC-MS/MS data were collected using a standard targeted metabolic profiling MS method developed in the NW-MRC that has been used in a growing number of studies (Carroll et al., 2015; Gu et al., 2015; Reyes et al., 2014; Sood et al., 2015; Sperber et al., 2015). Briefly, the LC-MS/MS experiments were performed on an Agilent 1260 LC (Agilent Technologies, Santa Clara, CA) AB Sciex QTrap 5500 MS (AB Sciex, Toronto, Canada) system. Each sample was injected twice, 15 µL for analysis using negative ionization mode and 5 µL for analysis using positive ionization mode. Both chromatographic separations were performed using hydrophilic interaction chromatography (HILIC) on the Waters XBridge BEH Amide column (150 x 2.1 mm, 2.5 µm particle size, Waters Corporation, Milford, MA). The flow rate was 0.3 mL/min. The mobile phase was composed of Solvents A (10 mM ammonium acetate in 90% H2O/ 5% acetonitrile/ 5% methanol + 0.3% acetic acid) and B (10 mM ammonium acetate in 85% acetonitrile/ 10% H2O / 5% methanol+ 0.3% acetic acid). After the initial 1.5 min isocratic elution of 90% B, the percentage of Solvent B was decreased to 45% at t=5 min. The composition of Solvent B was maintained at 45% for 5 min (t=10 min), and then the percentage of B was gradually increased to 90%, to prepare for the next injection. The metabolite identities were confirmed by spiking the pooled serum sample used for method development with mixtures of standard compounds. The extracted MRM peaks were integrated using MultiQuant 2.1 software (AB Sciex, Toronto, Canada).

**Metabolomics Data Analysis**

Targeted metabolomics and untargeted lipidomics datasets were first filtered by applying the 80% rule to keep only features present in most samples; the retained features were further filtered by variations between QC samples and among biological replicates (CV% of QCs <= 30% or CV% of biological replicates < 50%). Missing values were replaced with the median of the group. Finally, the data were log2 transformed and two-sample t-test was performed to detect differential metabolites.

**Ribo-Zero RNA-Seq and Data Analysis**

RNA sequencing libraries were prepared using the TruSeq Stranded Total RNA Library Prep Kit with Ribo-Zero H/M/R Gold, following the manufacturer's instructions. The libraries were sequenced as paired-end 76-mers on an Illumina NextSeq 500. Read pairs were filtered based on a mean base quality score more than 20, followed by adapter-trimming with CutAdapt (v1.2.1) (parameters –O 5 –q 0). Filtered and trimmed read pairs were mapped to the mm10 reference genome with STAR (Dobin et al., 2013) (v2.5) (parameters --outMultimapperOrder Random --outSAMattrIHstart 0 --outFilterType BySJout --alignSJoverhangMin 8 --limitBAMsortRAM 55000000000). Quantification of mapped hits per gene was calculated with Subread featureCounts v1.5.0-p1 (parameters -s2 -Sfr). Differentially expressed genes were identified by DESeq2 (Love et al., 2014) (v1.10.1) with a cutoff of padj<0.05 and |FC|>1.2. The RefSeq gene models downloaded from the UCSC Genome Browser on April 11, 2014, were used with gene Rn45s excluded for the analysis.

**Functional Annotation of Obesity-Related Differentially Expressed Genes**

VLAD (Visual Annotation Display; http://proto.informatics.jax.org/prototypes/vlad/) is a tool for visualizing GO annotations. VLAD (Version 1.5.1) was run with default parameters.

IPA core analysis was performed to interpret gene sets in the context of diseases and bio functions (molecular and cellular functions). To compare changes in biological states across conditions, IPA comparison analysis was performed on two core analyses, HF vs LF and HF-LF vs LF.

Reactome pathway enrichment analysis (Fabregat et al., 2016) was performed to determine which events (pathways and/or reactions) are statistically enriched in obesity-related differentially expressed genes. Reactome (http://reactome.org) is a free, open-source, curated and peer-reviewed knowledge-base of biomolecular pathways. Pathways in Reactome are organized hierarchically, grouping related detailed pathways into larger domains of biological function. This hierarchical organization largely follows that of the Gene Ontology (GO) biological process hierarchy. The results of the over-representation analysis are provided as a color-coded interactive list of events on the 'pathways overview' of the entire Reactome event hierarchy. Each event is colored according to the probability (from a hypergeometric test) of seeing a given number or more genes in this event by chance. The darker the color, the more significant of the over-representation for a given pathway.

**Single-sample GSEA (ssGSEA)**

ssGSEA (Barbie et al., 2009), an extension of Gene Set Enrichment Analysis (GSEA), calculates separate enrichment scores for each pairing of a sample and gene set. Each ssGSEA enrichment score represents the degree to which the genes in a particular gene set are coordinately up- or down-regulated within a sample. We used ssGSEAProjection (v7) in the GenePattern modules to calculate the enrichment scores of hallmark gene sets (Liberzon et al., 2015) (FATTY_ACID_METABOLISM and GLYCOLYSIS) for each sample in the TCGA Colon and Rectal Cancer (COADREAD) dataset, which was downloaded from UCSC Xena (http://xena.ucsc.edu/).

**Supplemental References**


Anders, S., Pyl, P.T., and Huber, W. (2015). HTSeq--a Python framework to work with high-throughput sequencing data. Bioinformatics *31*, 166-169.

Barbie, D.A., Tamayo, P., Boehm, J.S., Kim, S.Y., Moody, S.E., Dunn, I.F., Schinzel, A.C., Sandy, P., Meylan, E., Scholl, C., et al. (2009). Systematic RNA interference reveals that oncogenic KRAS-driven cancers require TBK1. Nature *462*, 108-112.

Buas, M.F., Gu, H., Djukovic, D., Zhu, J., Drescher, C.W., Urban, N., Raftery, D., and Li, C.I. (2016). Identification of novel candidate plasma metabolite biomarkers for distinguishing serous ovarian carcinoma and benign serous ovarian tumors. Gynecol Oncol *140*, 138-144.

Carroll, P.A., Diolaiti, D., McFerrin, L., Gu, H., Djukovic, D., Du, J., Cheng, P.F., Anderson, S., Ulrich, M., Hurley, J.B., et al. (2015). Deregulated Myc Requires MondoA/Mlx for Metabolic Reprogramming and Tumorigenesis. Cancer Cell *27*, 271-285.

Dobin, A., Davis, C.A., Schlesinger, F., Drenkow, J., Zaleski, C., Jha, S., Batut, P., Chaisson, M., and Gingeras, T.R. (2013). STAR: ultrafast universal RNA-seq aligner. Bioinformatics *29*, 15-21.

Fabregat, A., Sidiropoulos, K., Garapati, P., Gillespie, M., Hausmann, K., Haw, R., Jassal, B., Jupe, S., Korninger, F., McKay, S., et al. (2016). The Reactome pathway Knowledgebase. Nucleic Acids Res *44*, D481-487.

Gu, H., Zhang, P., Zhu, J., and Raftery, D. (2015). Globally Optimized Targeted Mass Spectrometry (GOT-MS): Reliable Metabolomics Analysis with Broad Coverage Anal. Chem. *87*, 12355-12362.

Halachev, K., Bast, H., Albrecht, F., Lengauer, T., and Bock, C. (2012). EpiExplorer: live exploration and global analysis of large epigenomic datasets. Genome Biol *13*, R96.

Heinz, S., Benner, C., Spann, N., Bertolino, E., Lin, Y.C., Laslo, P., Cheng, J.X., Murre, C., Singh, H., and Glass, C.K. (2010). Simple combinations of lineage-determining transcription factors prime cis-regulatory elements required for macrophage and B cell identities. Mol Cell *38*, 576-589.

Krueger, F., and Andrews, S.R. (2011). Bismark: a flexible aligner and methylation caller for Bisulfite-Seq applications. Bioinformatics *27*, 1571-1572.

Langmead, B., Trapnell, C., Pop, M., and Salzberg, S.L. (2009). Ultrafast and memory-efficient alignment of short DNA sequences to the human genome. Genome Biol *10*, R25.

Li, R., Grimm, S.A., Chrysovergis, K., Kosak, J., Wang, X., Du, Y., Burkholder, A., Janardhan, K., Mav, D., Shah, R., et al. (2014). Obesity, rather than diet, drives epigenomic alterations in colonic epithelium resembling cancer progression. Cell Metab *19*, 702-711.

Liberzon, A., Birger, C., Thorvaldsdottir, H., Ghandi, M., Mesirov, J.P., and Tamayo, P. (2015). The Molecular Signatures Database (MSigDB) hallmark gene set collection. Cell Syst *1*, 417-425.

Love, M.I., Huber, W., and Anders, S. (2014). Moderated estimation of fold change and dispersion for RNA-seq data with DESeq2. Genome Biol *15*, 550.

Rao, J.N.K., and Scott, A.J. (1981). The Analysis of Categorical-Data from Complex Sample-Surveys - Chi-Squared Tests for Goodness of Fit and Independence in 2-Way Tables. J Am Stat Assoc *76*, 221-230.

Rao, J.N.K., and Scott, A.J. (1984). On Chi-Squared Tests for Multiway Contingency-Tables with Cell Proportions Estimated from Survey Data. Ann Stat *12*, 46-60.

Rao, J.N.K., and Scott, A.J. (1987). On Simple Adjustments to Chi-Square Tests with Sample Survey Data. Ann Stat *15*, 385-397.

Reyes, N.L., Banks, G.B., Tsang, M., Margineantu, D., Gu, H., Djukovic, D., Chan, J., Torres, M., Liggitt, H.D., Hirenallur-S, D.K., et al. (2014). Fnip1 regulates skeletal muscle fiber type specification, fatigue resistance, and susceptibility to muscular dystrophy. Proc. Natl. Acad. Sci. U. S. A. *112*, 424-429.

Sood, R.F., Gu, H., Djukovic, D., Deng, L., Ga, M., Muffley, L.A., Raftery, D., and Hocking, A.M. (2015). Targeted metabolic profiling of wounds in diabetic and non-diabetic mice. Wound Repair Regen. *23*, 423-434.

Sperber, H., Mathieu, J., Wang, Y.L., Ferreccio, A., Hesson, J., Xu, Z.J., Fischer, K.A., Devi, A., Detraux, D., Gu, H.W., et al. (2015). The metabolome regulates the epigenetic landscape during naive-to-primed human embryonic stem cell transition. Nat. Cell Biol. *17*, 1523-1535.

Trapnell, C., Pachter, L., and Salzberg, S.L. (2009). TopHat: discovering splice junctions with RNA-Seq. Bioinformatics *25*, 1105-1111.

Wang, S., Sun, H., Ma, J., Zang, C., Wang, C., Wang, J., Tang, Q., Meyer, C.A., Zhang, Y., and Liu, X.S. (2013). Target analysis by integration of transcriptome and ChIP-seq data with BETA. Nat Protoc *8*, 2502-2515.

Zhao, H., Sun, Z., Wang, J., Huang, H., Kocher, J.P., and Wang, L. (2014). CrossMap: a versatile tool for coordinate conversion between genome assemblies. Bioinformatics *30*, 1006-1007.
